# Computational identification of B- and T-cell epitopes: a unified task taxonomy and review of databases, datasets, predictive pipelines, and gaps

**DOI:** 10.3389/fimmu.2026.1873135

**Published:** 2026-09-17

**Authors:** Muhammad Ali Ibrahim, Arooj Zaib, Tayyaba Asif, Andreas Dengel, Jens Posma, Wolfram Ruf, Muhammad Nabeel Asim

**Affiliations:** 1 Department of Computer Science, Rhineland-Palatinate Technical University of Kaiserslautern-Landau, Kaiserslautern, Germany; 2 Smart Data & Knowledge Services, German Research Center for Artificial Intelligence, Kaiserslautern, Germany; 3 Center for Thrombosis and Hemostasis, University Medical Center of the Johannes Gutenberg University, Mainz, Germany; 4 Department of Immunology and Microbiology, Scripps Research, La Jolla, CA, United States; 5 German Center for Cardiovascular Research (DZHK), Partner Site Rhein-Main, Mainz, Germany; 6 Graduate School of Informatics, Osaka Metropolitan University, Sakai, Japan

**Keywords:** antigen epitope recognition, artificial intelligence, B-cell epitopes, computational immunology, dataset benchmarking, epitope databases, T-cell immunoinformatics, deep learning and protein language models

## Abstract

The immune system leverages B and T cells to recognize specific molecular patterns, known as epitopes, on pathogens and cancer cells to effectively combat infections and diseases. The critical success of immunotherapies in cancer treatment and COVID-19 vaccine development has established precise epitope identification as a central and rapidly growing priority in therapeutic design. Because traditional wet-lab based identification of B- and T-cell epitopes is expensive and timeconsuming, our systematic literature search (2015–2026) identified 148 Artificial Intelligence (AI) based linear B-cell, conformational B-cell, and T-cell epitope prediction models within the stated search scope. However, the true potential of these models remains unclear due to fragmented evaluation practices, with models rarely tested across a comprehensive range of datasets, insufficient comparison with existing predictors, systematic under-utilization of available public databases, and other methodological inconsistencies across five different stages of the predictive pipeline. Moreover, the 7 existing review papers fail to adequately highlight these research gaps or to support the development of robust AI models. This review paper consolidates the computational landscape of B-cell and T-cell epitope recognition and introduces a unified taxonomy that organises the field into twelve prediction tasks: linear and conformational B-cell prediction together with ten T-cell subtasks (T1–T10) that span the antigen-recognition cascade from human leukocyte antigen (HLA) typing to vaccine design. It analyses these twelve tasks across five major stages of a shared predictive pipeline. Within this taxonomy, the recognition sub-tasks (T1–T7) apply to the epitopes of any antigen, whereas neoantigen identification (T8) and tumor T-cell antigen (TTCA) classification (T9) are oncology-specific translational applications and multi-epitope vaccine design (T10) spans infectious-disease and cancer targets. It systematically examines 155 studies published from 2015 to 2026 to perform comprehensive categorization of 43 dedicated epitope/immunology databases, 144 benchmark datasets, 272 representation learning approaches, 148 classifiers, 54 evaluation and optimization approaches, and 148 predictive models accessibility status across all twelve prediction tasks. Additionally, it highlights persistent challenges across each stage of the predictive pipeline and offers key directions for improvement. This comprehensive analysis provides actionable recommendations for developing more robust, generalizable epitope predictors, and it ultimately accelerates the translation of computational predictions into effective immunotherapies against diverse diseases.

## 1 Introduction

The human immune system is a complex network of cells, tissues, and organs that work together to defend the body against foreign invaders such as pathogens^1^ (1, 2). It is commonly divided into two main branches: the innate immune system, which provides an immediate, non-specific response, and the adaptive immune system, which generates antigen-specific defense mechanisms and immunological memory. B and T cells are critical components of the adaptive immune system as they provide specific and long-term protection (3–7). Specifically, B-cells, also known as B lymphocytes, play a vital role in humoral immunity by producing antibodies (immunoglobulins) (2) in four different steps shown in the **Figure 1**. These antibodies recognize and bind to specific regions on antigens, called B-cell epitopes (BCEs) (8–12). This binding can neutralize pathogens or mark them for destruction by other immune cells (1, 13). B-cell epitopes can be either linear, which consist of a contiguous sequence of amino acids, or conformational, which comprise amino acids that are discontinuous in the sequence but are brought together by the protein’s three-dimensional folding (2, 4, 6–8, 10, 12, 14, 15). B-cells target both kinds of antigen BCEs and stimulate the immune system to fight against infections caused by different pathogens (5). On the other hand, T-cells, or T lymphocytes directly attack infected, cancerous, or foreign cells without employing any antibodies (2, 16–21). It is evident in the **Figure 1** that, unlike B-cells, which recognize epitopes on free or surface-bound antigens, T-cells recognize short peptide fragments of antigens presented on the surface of cells by major histocompatibility complex (MHC) molecules (11, 16–18, 20, 22–24). There are two main types of T-cells: CD4^+^ helper T-cells and CD8^+^ cytotoxic T-cells. Helper T-cells secrete cytokines that help activate other immune cells, including B-cells, to produce high-affinity antibodies (11, 16). In contrast, cytotoxic T-cells directly kill infected or cancerous cells by recognizing specific T-cell epitopes presented by MHC class I molecules (11, 17).

**Figure 1:**
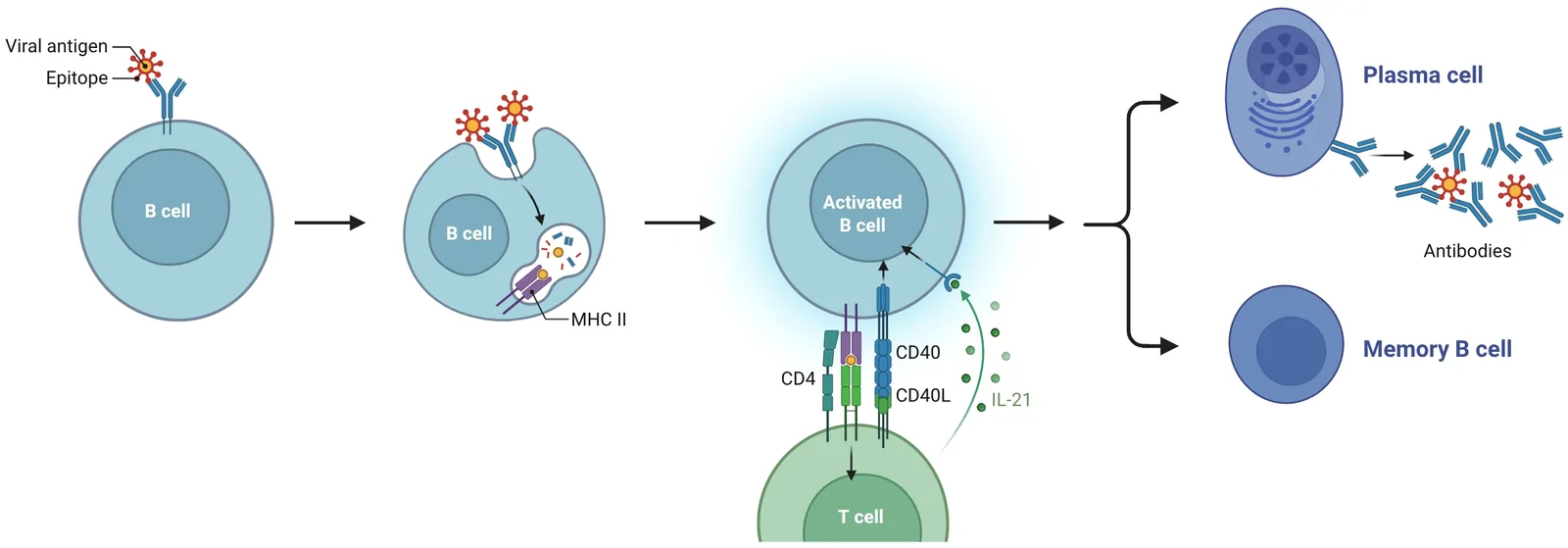
Activation of B cells and T cells and two-way interactions between these cells. Left to right panel: B cells internalize and process antigens only when their B-cell receptor (BCR) recognizes specific epitopes on viral coat proteins, followed by MHC II presentation. Antigen recognition induces B-cell proliferation and differentiation into antibody-secreting plasma cells and long-lived memory B cells. MHC, major histocompatibility complex; IL-21, interleukin-21.

The interaction between B and T cells is critical for a coordinated and effective immune response (25). As is shown in **Figure 1**, CD4^+^ helper T-cells play a significant role in the activation and maturation of B-cells. When a B-cell binds its specific epitope on an antigen and internalizes it, the antigen is processed into peptide fragments, which are presented on the B-cell surface bound to MHC class II molecules. CD4^+^ helper T-cells that recognize these peptide-MHC complexes, can bind to the Bcell and provide stimulatory signals through direct cell-cell contact and cytokine secretion. These signals are necessary for B-cell proliferation, differentiation into antibody-secreting plasma cells, and affinity maturation to produce high-affinity antibodies (11, 16). This interaction ensures that antibody production is tailored to the specific antigen to generate a robust and long-lasting humoral immune response (1, 4–7, 16). This highlights the interconnected roles of CD4^+^ helper T-cells and B-cells in responding to pathogens. The intricate relationship between the adaptive immune system’s B and T cells, driven by their specific recognition of epitopes, forms the foundation for rational vaccine design, immunodiagnostics, and the development of immunotherapies (11).

To harness this relationship, modern immunology focuses on accurately identifying B and T cells epitopes for the rapid development of highly targeted vaccines and diagnostic tools. However, the identification of these immune targets presents a complex challenge due to significant variations in their size, structure, and capacity to elicit an immune response (immunogenicity) (1, 16). For instance, linear B-cell epitopes typically range from 5 to 20 amino acids in length as continuous stretches, whereas conformational B-cell epitopes are defined by spatial clusters of amino acids that may be discontinuous in sequence but come together in the 3D structure, and they typically involve clusters of about 20 to 70 residues that participate in antibody binding. While linear B-cell epitopes are amenable to analysis through sequence-based modeling, conformational B-cell epitopes necessitate computational modeling of protein folding (1, 2). B-cell epitopes must be surface accessible on the antigen to be recognized by circulating antibodies or B-cell receptors. Their immunogenicity depends on factors such as their affinity for the B-cell receptor, their location on the antigen, and the overall context of the antigen (1, 2). On the other hand, T-cell epitopes are generally short peptide fragments, typically 8–11 amino acids for MHC class I and 13–25 for MHC class II presentation. These peptide fragments are generated through intracellular antigen processing and must be presented on the surface of antigen-presenting cells (APCs) bound to major histocompatibility complex (MHC) molecules for recognition by T-cell receptors (TCRs) (16–18). The TCR interacts with both the peptide and the MHC molecule. Therefore, the recognition structure of a T-cell epitope is the complex of the peptide and the MHC molecule. For T-cell epitopes, immunogenicity is influenced by the affinity of the peptide for the MHC molecule (MHC binding affinity), the stability of the peptide-MHC complex, and the ability of the TCR repertoire to recognize the presented peptide (16–18).

Taking the challenges of B-cell and T-cell epitope identification into account, to date, researchers have employed two main approaches: wet lab experimental methods, and Artificial Intelligence based methods. The traditional wet-lab methods for epitope identification, such as X-ray crystallography, nuclear magnetic resonance (NMR), peptide arrays, and MHC tetramer assays, are often tedious, expensive, and time-consuming (1, 16). The need to screen large numbers of potential epitopes further exacerbates these limitations. To overcome these constraints, computational immunoinformatics has emerged as a powerful and cost-effective approach. By leveraging machine learning, deep learning, and structural bioinformatics, these in silico tools aim to accurately predict potential B-cell and T-cell epitopes based on sequence and structural features. This approach reduces the pool of candidate epitopes for experimental validation and accelerates the development of vaccines, diagnostics, and immunotherapies. To date, researchers have developed 45 AI based B-cell epitope predictors and 103 T-cell epitope predictors spanning ten prediction sub-tasks (T1– T10) using diverse approaches at various stages of the predictive pipeline and evaluated them on 144 datasets encompassing multiple pathogen families. However, the epitope prediction literature remains highly fragmented, with individual studies often focusing on specific epitope types. Furthermore, existing studies fail to conduct a comprehensive evaluation using all available datasets under varied experimental settings. Moreover, they fail to conduct a fair performance comparison with all existing epitope specific predictors. As a result, there is limited clarity regarding the true potential of these models in terms of their predictive powers, generalizability, and ability to adapt to different epitope types. This lack of standardization and cohesion hampers meaningful comparisons between models and limits their applicability to real-world therapeutic design. It sharply hinders the ability of both immunologists and computational scientists to select appropriate models and build upon existing work. Therefore, there is a critical need to unify the epitope prediction literature through structured synthesis, comparative analysis, and pipeline-level mapping of existing models.

To address this critical need and accelerate the development of robust, generalizable epitope prediction pipelines, we present the first comprehensive, multi-dimensional analysis that systematically unifies the fragmented landscape of computational epitope identification. We provide an authoritative resource for both computational scientists and immunologists by offering a comprehensive and structured overview of AI-based approaches for the identification of linear b-cell epitopes, conformational b-cell epitopes, and t-cell epitopes. The overall organization of this review is summarized in **Figure 2**, which outlines the progression from the research methodology and prior-review analysis through databases, benchmark datasets, predictive pipelines, performance analysis, and key research gaps toward future directions. Specifically, the key contributions of this review are as follows:

**Figure 2:**
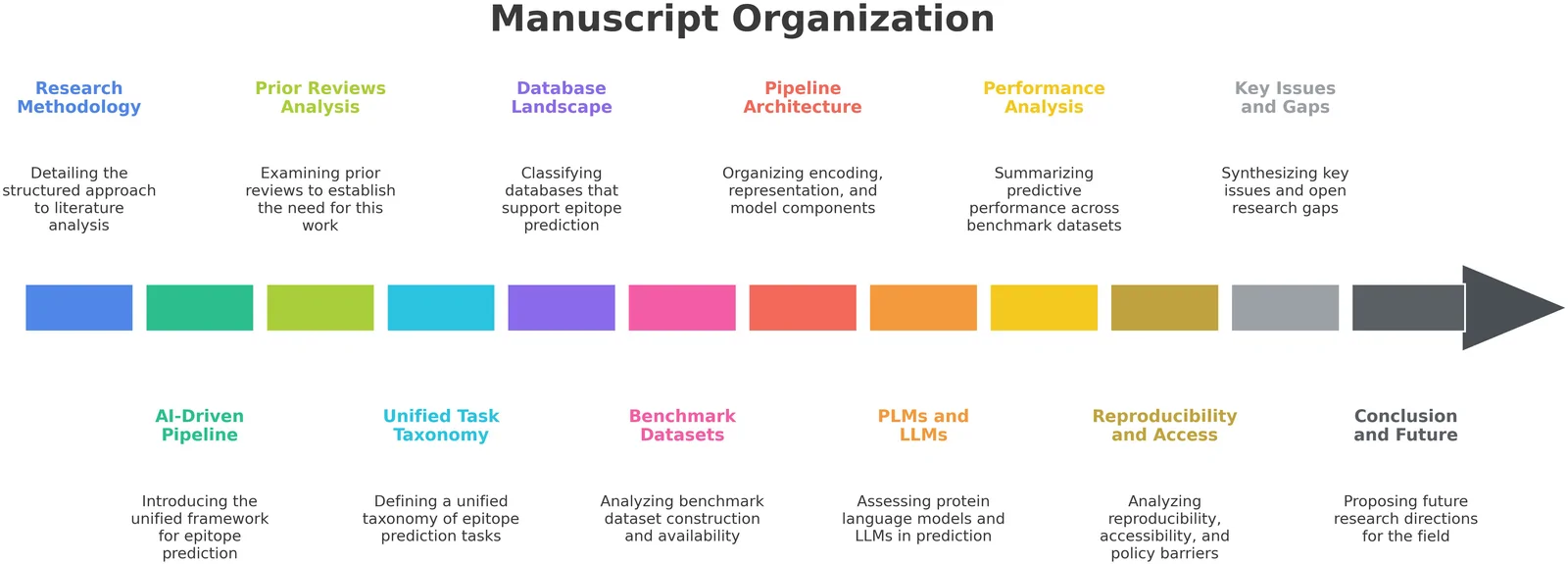
An overview of manuscript organization.

- It introduces a unified taxonomy that decomposes computational epitope prediction into twelve tasks across the antigen-recognition cascade, namely linear and conformational B-cell prediction together with ten T-cell sub-tasks (T1–T10) that range from human leukocyte antigen (HLA) typing and antigen processing through MHC class I and class II binding, immunogenicity, and TCR specificity to neoantigen identification, tumor T-cell antigen classification, and multi-epitope vaccine design. The first seven T-cell sub-tasks (T1–T7) constitute a general recognition cascade that applies to the epitopes of any antigen. Neoantigen identification (T8) and tumor T-cell antigen classification (T9) are translational applications specific to the oncology setting, whereas multi-epitope vaccine design (T10) applies across infectious-disease and cancer targets. This taxonomy provides the unifying framework that organises the entire review.
- It systematically consolidates and analyzes 43 dedicated epitope/immunology databases, distinguished from 5 supporting resources (3 general-purpose sequence databases and 2 structural repositories), that support linear B-cell, conformational B-cell, and T-cell epitope prediction research. This analysis provides a five-dimensional overview including their classification (primary, specialized, supplementary, structural repository, general-purpose), scope, scale, accessibility and maintenance status, and usage patterns (widely adopted, underutilized, niche applications).
- It comprehensively examines 144 benchmark datasets used for training predictive models, and it details their construction methodologies (single-database and multi-database integration strategies), availability landscape (public, private, accessible), species coverage, and compositional diversity across all twelve epitope prediction tasks.
- It identifies and categorizes 272 unique representation learning approaches into 13 distinct categories and provides key trends and critical remarks on the effectiveness and limitations of these approaches across all twelve epitope prediction tasks. It highlights striking patterns of methodological specialization and identifies significant opportunities for cross-task knowledge transfer.
- It identifies and categorizes 148 distinct classification methodologies into five categories (Statistical Models, Machine Learning, Deep Learning, Ensemble, Hybrid) and facilitates key trends and crucial insights on the effectiveness and limitations of these methodologies across the twelve epitope prediction tasks. It reveals distinct preferences and specialization patterns across different epitope prediction tasks. It highlights which methodologies are fastest-growing and being extensively adopted across all prediction tasks.
- It identifies 25 distinct evaluation measures, and 29 optimization (feature selection and hyperparameter optimization) strategies employed with their task-specific utilization and highlights the extensively adopted approaches across all studies. It comprehensively identifies and organizes current research gaps and limitations related to evaluation, such as the lack of comprehensive evaluation, direct comparison, cross-task generalization. Overall, it organizes several research gaps and limitations across five major stages of a unified predictive pipeline.
- It provides a detailed performance analysis of predictive models across epitope types for benchmark datasets under the hood of different expxerimental settings and evaluation measures. It reveals key issues like systematic overfitting, stagnation in performance improvements despite methodological innovations, and a critical lack of external validation beyond core benchmark datasets.
- It analyzes the landscape of deployment and accessibility of existing epitope prediction tools across 148 studies, including code availability, web server deployment, and platform preferences. It reveals significant disparities and critical gaps that impede research reproducibility and practical application adoption.
- It proposes promising future research directions for developing more accurate, generalizable, adaptable, and interpretable epitope predictors, including calls for improved data curation (e.g., with detailed metadata), advanced representation learning (e.g., protein language models, transfer learning), expanded deep learning architectures (e.g., graph neural networks, attention mechanisms), comprehensive and consistent evaluations (including cross-task generalization), increased model deployment and accessibility, and multi-omics integration.

## 2 Systematic research methodology

Computational epitope prediction has evolved into distinct research streams for different epitope types, including linear B-cell, conformational B-cell, and T-cell epitope identification to develop type-specific predictive approaches. The maturation of the field brings forth an urgent requirement to transcend epitope-type specific approaches and establish a comprehensive understanding of methodological trends, evaluation practices, shared challenges, and key research gaps across all epitope prediction tasks. To fulfil this requirement, we have employed a structured research methodology illustrated in **Figure 3** to systematically identify, analyze, and unify relevant computational epitope prediction literature. Designed research methodology follows a two-stage process: (1) article identification and retrieval, and (2) article screening and selection, necessary details of which are provided in following subsections.

**Figure 3:**
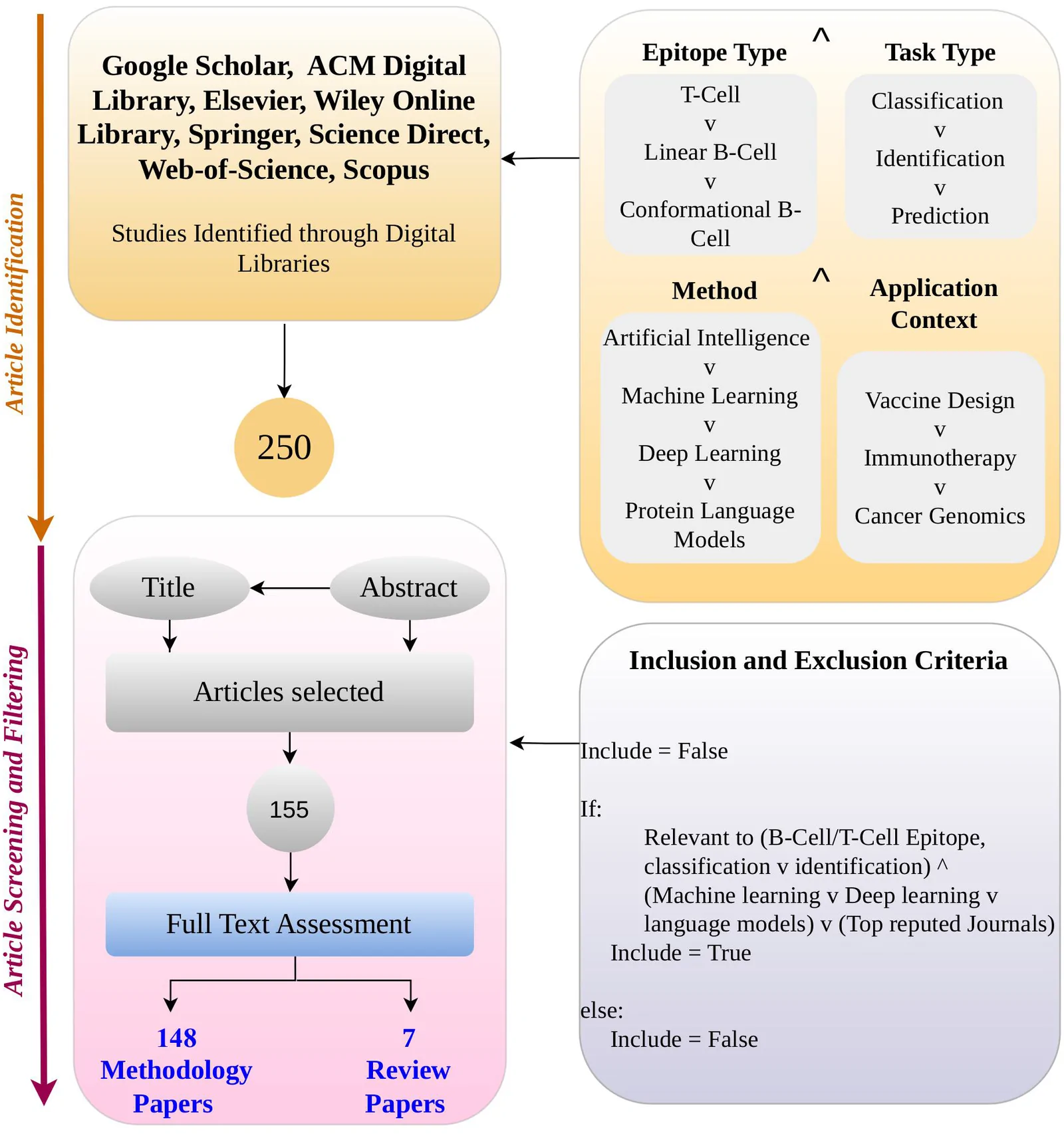
A two-stage strategy for systematic literature review.

### 2.1 Article identification and retrieval

With an aim to identify scholarly articles relevant to the twelve epitope prediction tasks, we first formulated extensive structured search queries using three conceptual keyword groups: epitope prediction tasks (e.g., “linear B-cell epitope”, “conformational B-cell epitope”, “T-cell epitope”), computational techniques (e.g., “machine learning,” “deep learning,” “protein language models”), and application contexts (e.g., “vaccine design,” “cancer immunotherapy”). Within each group, keywords are combined using the OR (∨) operator, and different groups are combined using the AND (∧) operator. These queries are executed across multiple academic search platforms, including Google Scholar, ACM Digital Library, Elsevier, Wiley Online Library, Springer, Science Direct, Scopus, and Web of Science covering publications from 2015 to 2026. Apart from direct search, snowballing techniques are also applied to reference lists and citation networks to expand search coverage and identify more articles. Execution of search queries across different academic databases has helped us to obtain 250 research articles which are screened in second stage.

### 2.2 Article screening and filtering

The initial search retrieved 250 articles spanning 7 different application contexts of epitope prediction. These application contexts shown in **Figure 4** include (1) vaccine development for rapid pandemic response, (2) cancer immunotherapy for neoantigen identification, (3) infectious disease research targeting pathogens like SARS-CoV-2, (4) allergy research for hypoallergenic vaccine design, (5) antibody engineering for therapeutic development, (6) autoimmune disease therapies, and (7) diagnostic tool development for improved immunodiagnostics (26). Many studies have merely applied existing epitope prediction methods within these contexts without contributing methodological innovations. To maintain focus on computational advancements in epitope prediction, we have implemented a two-phase screening process.

**Figure 4:**
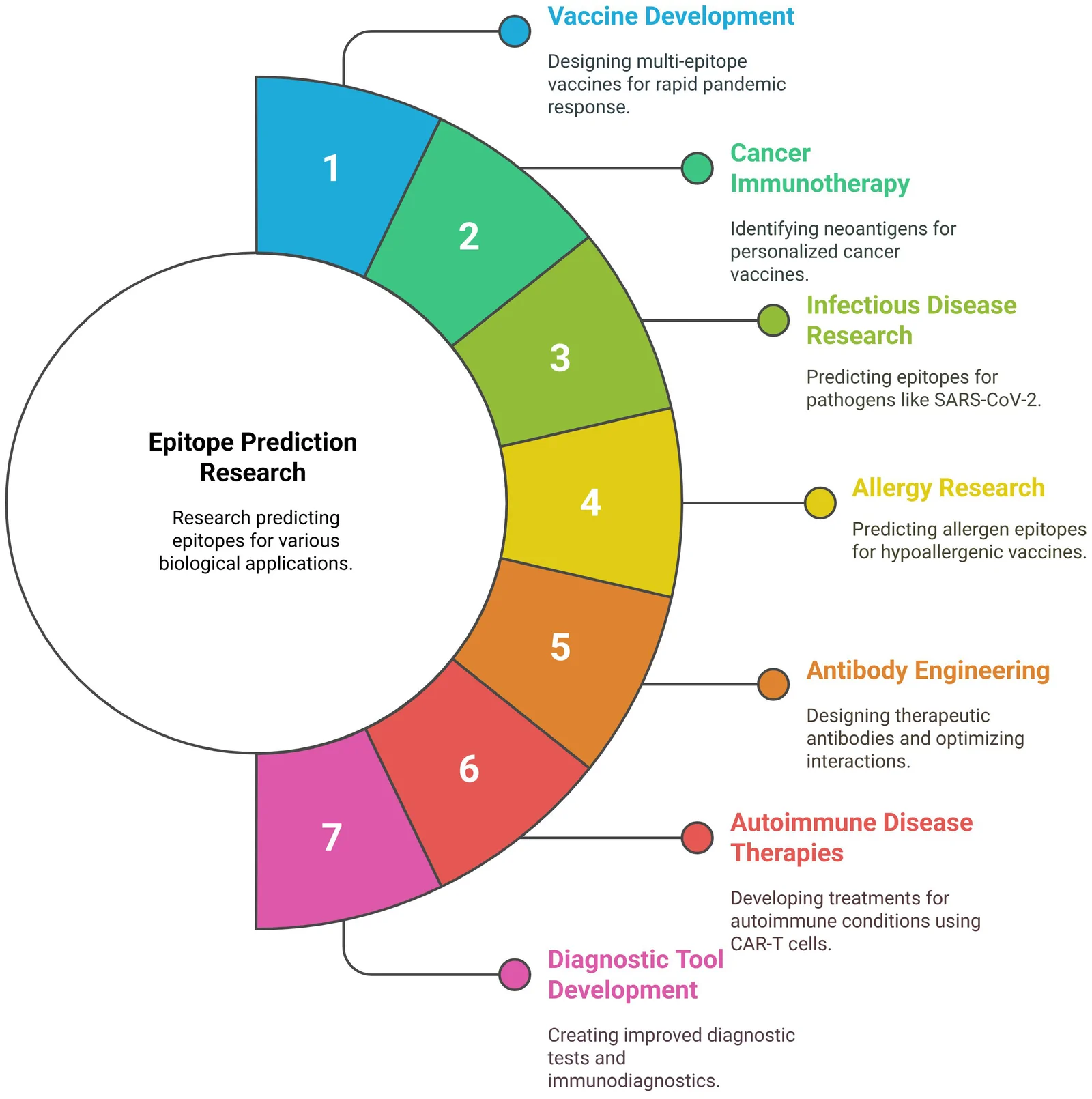
Application contexts of computational epitope prediction literature.

In the first screening phase, we have evaluated titles and abstracts of 250 articles against predefined inclusion and exclusion criterion emphasizing methodological contributions to AI-based epitope prediction.

#### 2.2.1 Inclusion criteria

- Research articles primarily focused on one or more of the twelve epitope prediction tasks, namely linear B-cell epitope prediction, conformational B-cell epitope prediction, or any of the ten T-cell prediction sub-tasks (T1–T10)
- Research articles employing machine learning or deep learning approaches for epitope prediction
- Research article with clear descriptions of datasets, feature extraction techniques, and pipelines, and evaluation metrics
- Research articles published in peer-reviewed journals or high-quality conference proceedings between 2015 and 2026

#### 2.2.2 Exclusion criteria

- Research articles where epitope prediction is used as a secondary tool rather than the primary methodological focus (e.g., vaccine design, immunotherapy development, drug discovery)
- Research articles focused primarily on antigen or antibody design rather than epitope prediction
- Research articles using exclusively wet lab experimentation without computational prediction methods

This initial assessment has identified 250 potentially relevant papers. In the second phase, we have conducted comprehensive full-text evaluations examining methodological clarity, dataset composition and handling, evaluation protocols, performance metrics, and reproducibility factors including code or tool availability. This rigorous full-text screening, complemented by citationnetwork snowballing of the identified literature, has yielded a final collection of 155 studies that cover the twelve epitope prediction tasks, namely linear and conformational B-cell prediction together with the ten T-cell sub-tasks (T1–T10). Among the 155 articles, 32 focus on linear B-cell epitope prediction, 13 address conformational B-cell epitope prediction, 103 perform T-cell epitope prediction across the full ten-sub-task landscape that spans MHC class I and class II binding, antigen processing, immunogenicity, TCR specificity, neoantigen identification, structural TCR modelling, HLA typing, and vaccine design, and 7 articles review epitope prediction across both the B-cell and T-cell branches. This systematically curated collection of studies provides the foundation for our comparative analysis of datasets, methodologies, challenges, and future directions across the twelve epitope prediction tasks.

### 2.3 Counting conventions

The quantities catalogued across the twelve tasks follow different counting conventions, because the same rule does not apply to every entity type.

*Databases:* Section 6 Epitope prediction databases landscape analysis catalogues 48 entries, and these are not counted as a single undifferentiated total, because they span resources of different kinds. Following the three-way distinction drawn in the databases section, 43 are dedicated epitope, receptor, or immunology databases (the Primary, Specialized, and auxiliary immunology resources), 3 are general-purpose sequence databases (UniProt, NCBI GenBank, and SwissProt), and 2 are structural repositories used as source material for conformational and structural-TCR datasets (the Protein Data Bank, accessed through the RCSB PDB portal, and STCRDab). The headline epitope-resource figure is therefore the 43 dedicated databases, and the general-purpose and structural repositories are tallied separately so that general bioinformatics infrastructure does not inflate it. Because a resource that serves more than one epitope branch, such as IEDB, is listed once under each branch that it supports, these are catalogued entries rather than wholly distinct resources, and 14 entries (29%) recur across branches in this way.
*Datasets:* The 144 benchmark datasets are counted as distinct datasets rather than one per study, because a single study frequently introduces several datasets while many studies reuse the same shared benchmark. Each distinct dataset is therefore counted once regardless of how many studies use it, and a dataset that is re-derived with a materially different composition, split, or labelling is counted as a separate dataset even when it descends from an earlier resource. The total comprises linear B-cell (57), conformational B-cell (27), and the ten T-cell sub-tasks (60 in total). The linear set is listed in **Supplementary Table 1**, the conformational set in section 7.2 Benchmark Datasets Availability, and the per-sub-task T-cell sets in **Supplementary Table 3**, and the per-task totals appear in section 7.2 Benchmark Datasets Availability. This enumeration is non-exhaustive, because large shared corpora such as the NetMHCpan and NetMHCIIpan eluted-ligand data, VDJdb, 10x Genomics, ImmuneCODE, IMMREP22, and Hi-TpH spawn many derived training and evaluation splits that are counted once rather than individually.
*Representation learning approaches:* The 272 representation learning approaches are counted as usage instances across the thirteen encoding categories of section 8.1 Sequence representation learning approaches rather than as distinct studies, because a single model usually combines several encodings, for example amino-acid composition together with physicochemical descriptors and a language-model embedding. The total therefore exceeds the model count and divides into linear B-cell (65), conformational B-cell (40), and T-cell (167).
*Models and classifiers:* The 148 predictive models are counted at one principal model per primary publication, so the classifier count equals the model count, because each publication contributes one predictor and one dominant classifier paradigm. They comprise 32 linear B-cell, 13 conformational B-cell, and 103 T-cell predictors. The 103 T-cell models distribute across the ten sub-tasks as T1 HLA typing and imputation (3), T2 antigen processing (5), T3 MHC class I binding (17), T4 MHC class II binding (12), T5 immunogenicity (12), T6 TCR–peptide-MHC specificity (23), T7 structural TCR modelling (4), T8 neoantigen identification (9), T9 tumor T-cell antigen (TTCA) classification (14), and T10 multi-epitope vaccine design (4). For HLA typing (T1) only the three machine-learning and deep-learning imputation models (CookHLA, DEEP-HLA, and HLARIMNT) are counted, because the review is scoped to machine learning and deep learning, and the read-based typing tools that rely on sequence alignment or other non-learning algorithms (for example HLA-HD, HLAminer, HISAT-genotype, and HaploSFHI) are discussed as the complementary paradigm but excluded from the tally. Derivative tools, namely updated versions, implementation variants, or web servers that re-package an existing algorithm from the same study, are not counted separately. The remaining 7 of the 155 examined papers are cross-branch review articles and are excluded from the predictor count, whereas additional task-specific review and benchmark articles, for example IMMREP22, Hi-TpH, and PepBenchmark, are cited as supporting literature.
*Evaluation and optimization schemes:* The 54 evaluation and optimization approaches comprise twenty-five distinct evaluation measures and twenty-nine optimization strategies, namely sixteen feature-selection strategies and thirteen hyperparameter-optimization strategies, and each scheme is counted once as a distinct method irrespective of how many studies adopt it. The twenty-five evaluation measures consist of a core of twelve classification measures together with thirteen further regression, agreement, ranking, and structural-quality measures that the T-cell sub-tasks require.

Across all of these quantities the enumeration is systematic but bounded by the search scope of peer-reviewed publications from 2015 to 2026 retrieved from eight academic databases, so resources or models outside this scope may exist.

## 3 Unified AI-driven epitope prediction pipeline

Through a comprehensive analysis of 155 published studies, we derive a unified AI-driven epitope prediction pipeline that reflects commonly adopted methodological stages and task-specific components underlying computational immunoinformatics. In computational immunoinformatics, several tasks converge on the broader goal of identifying epitope regions recognized by the adaptive immune system and predicting the specificity and strength of these interactions (27). However, the prediction of linear and conformational B-cell epitopes together with the ten T-cell sub-tasks of the antigen-recognition cascade is essential for understanding the complexities of the adaptive immune response. These tasks have significant implications for vaccine design, diagnostics, and immunotherapeutics. To highlight the interrelated nature and distinct challenges of these predictive tasks, **Figure 5** presents a unified predictive pipeline that outlines the nature of these tasks, the stages involved in epitope prediction, and the specific considerations associated with each task. **Figure 5** organizes the AI-driven predictive paradigms for linear B-cell, conformational B-cell, and T-cell epitope identification into five interconnected stages. These five stages form a common methodological skeleton that is shared across all twelve prediction tasks of the taxonomy presented in Section 5, even though the concrete realisation of each stage differs from one task to another. The first stage involves the collection and curation of high-quality benchmark datasets. These datasets are either adopted from existing studies or constructed from scratch. For the B-cell tasks and the peptide-level T-cell tasks they draw on experimentally validated epitopes from public repositories such as the Immune Epitope Database (IEDB), whereas other tasks rely on different source data, for example HLA genotypes and population single-nucleotide-polymorphism panels for HLA typing (T1), curated TCR repertoires for TCR specificity (T6), and experimentally solved three-dimensional complexes for structural modelling (T7).

**Figure 5:**
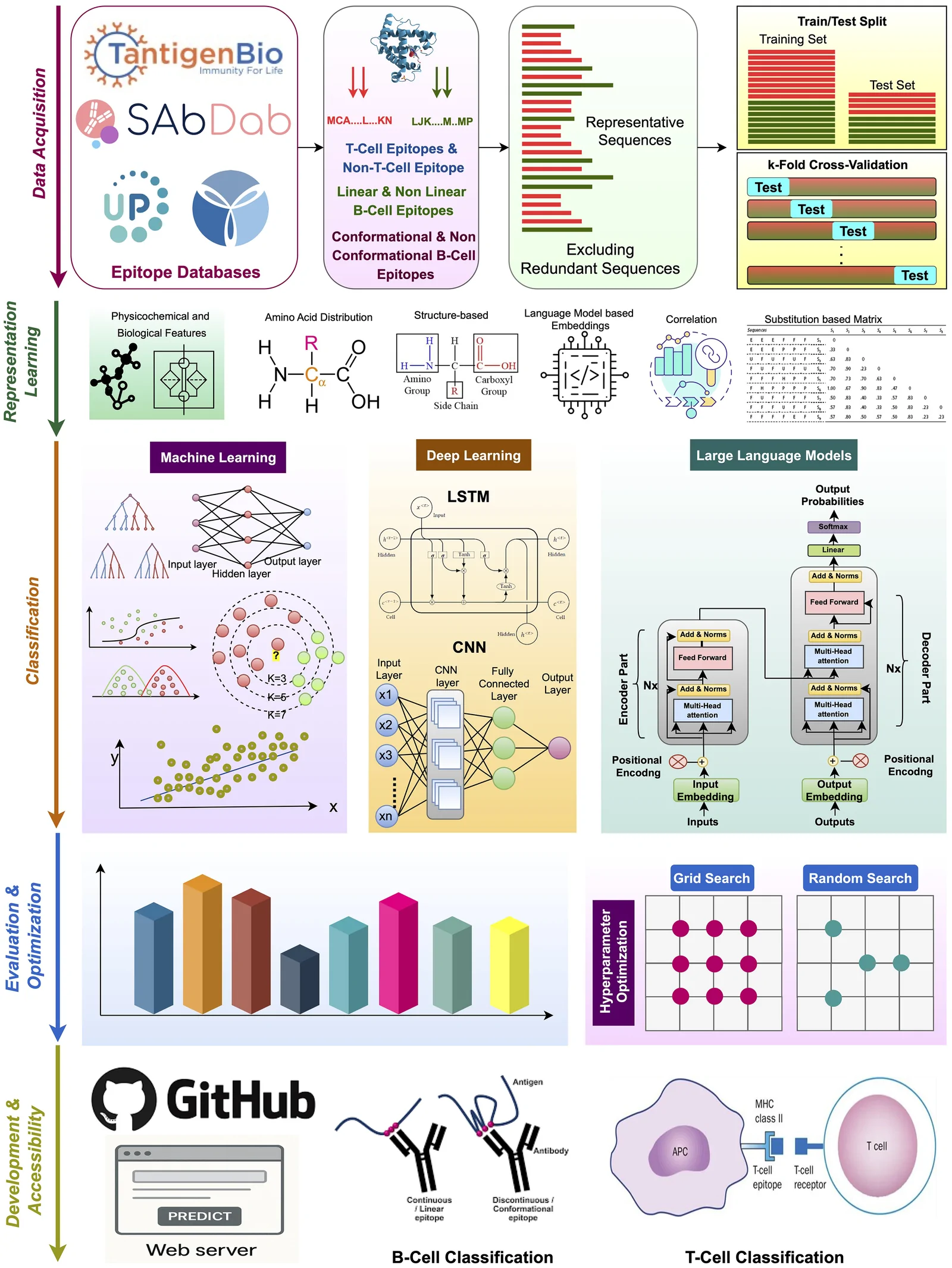
A unified five-stage predictive pipeline shared across the twelve epitope prediction tasks. The five stages form a common methodological skeleton whose concrete instantiation varies by task. The per-task decomposition is detailed in the taxonomy of **Figure 6**.

Comprehensive, accurate, and diverse datasets serve as the foundation for training robust predictive models across all twelve prediction tasks. The second stage is representation learning, where raw inputs are transformed into informative numerical vectors suitable for machine learning models. For most tasks these inputs are antigen sequences, either full proteins or peptide fragments, but the relational and upstream tasks encode other entities, namely paired peptideMHC inputs for class I and class II binding (T3 and T4), paired TCR and peptide-MHC inputs for TCR specificity (T6), and single-nucleotide-polymorphism genotypes for HLA imputation (T1). This transformation typically involves encoding sequence-based features using techniques like one-hot encoding, physicochemical property vectors, sequence embeddings, or protein language models. For conformational B-cell epitope prediction, additional structural features are used to describe the protein surface and 3D shape, which are important for antibody binding. These include solvent accessibility (accessible surface area, ASA, and relative solvent accessibility, RSA), residue protrusion, surface shape, spatial neighbourhood, and flexibility (B-factors or the AlphaFold2 predicted Local Distance Difference Test, pLDDT), and they are examined in detail in Section 8. Furthermore, in the third stage, the resulting numerical representations are used as input to machine learning or deep learning algorithms, and the prediction target itself varies by task. Linear and conformational B-cell prediction, antigen processing (T2), and tumor T-cell antigen classification (T9) label sequence regions or peptides as epitopes or non-epitopes. MHC binding (T3 and T4) is commonly cast as binding-affinity or presentation regression. TCR specificity (T6) is a relational classification over paired receptor and peptide-MHC inputs. Structural TCR modelling (T7) predicts and scores a three-dimensional complex. HLA imputation (T1) infers alleles from genotypes. Neoantigen identification (T8) and multi-epitope vaccine design (T10) are integrative pipelines that chain several of these predictions to rank or assemble candidates. Depending on the task, different model architectures may be employed, including traditional classifiers like support vector machines (SVMs) and tree-based ensembles such as random forests and gradient-boosted trees, and more advanced approaches such as convolutional neural networks (CNNs), recurrent neural networks (RNNs), long short-term memory (LSTM) networks, attention-based transformers, graph neural networks (GNNs) that operate on structural contact graphs, protein language models used directly as predictors, and adapted protein-structure frameworks such as AlphaFold2 and AlphaFold3 for the structural tasks.

The fourth stage focuses on the evaluation and optimization of the predictive pipeline. Here, standard evaluation metrics including accuracy, precision, recall, area under the curve (AUC), and Matthews correlation coefficient (MCC) are commonly used to assess model performance followed by optimization of model hyperparameters using different optimization strategies (e.g Grid Search, random search, or Bayesian optimization). The final stage focuses on the deployment and accessibility of developed models to ensure that AI-based predictors for linear B-cell, conformational B-cell, and T-cell epitopes are practically usable for applications like vaccine design and immunotherapeutics. Deployment involves integrating models into user-friendly platforms, such as web servers, standalone software, or APIs. Accessibility emphasizes open-source availability, comprehensive documentation, and compatibility with diverse computational environments to enable broad adoption by immunologists and clinicians. At this stage, key challenges include optimizing computational efficiency, particularly for conformational B-cell epitope predictors requiring structural modeling, and designing intuitive interfaces for non-expert users. By addressing these challenges, this stage maximizes the impact of predictive models in real-world immunological research. A comprehensive evaluation of epitope prediction tasks across diverse datasets using distinct evaluation measures and a fair comparison helps to identify robust and generalizable models and their limitations. The following sections analyse each stage in turn across all twelve tasks: the data foundation in the databases (Section 6) and benchmark datasets (Section 7), representation learning and classification (Section 8), the cross-cutting role of protein language models (Section 9), performance and evaluation (Section 10), and deployment and accessibility (Section 11).

## 4 A look back: analysis of epitope prediction reviews

To date, according to our best knowledge, a comprehensive analysis of the 155 included studies yields only 7 cross-branch reviews that span both the B-cell and T-cell branches of epitope prediction in different capacities. To establish the necessity of our comprehensive analysis, we systematically examined these 7 cross-branch epitope prediction reviews and critically analyzed their scope, methodological coverage, and fundamental limitations in **Table 1**. This examination reveals substantial gaps that severely constrain the field’s advancement and prevent effective translation of computational predictions into practical immunological applications. Our analysis evaluates these reviews across six critical methodological dimensions where systematic gaps prevent evidencebased method selection. These dimensions correspond to the five stages of the predictive pipeline, and the data-acquisition stage divides into the two axes of database classification and benchmark dataset coverage. The dimensions are database classification frameworks, benchmark dataset coverage, documentation, and availability, representation learning paradigms categorization, classifier utilization patterns, evaluation standardization protocols, and predictor accessibility and deployment characteristics. This systematic examination reveals that while reviews have evolved from basic resource listing to broader coverage and practical implementation guidance, fundamental analytical frameworks remain incomplete. A further limitation cuts across all six dimensions. Each of these reviews treats T-cell epitope prediction as a single undifferentiated task, whereas in practice it spans a cascade of distinct computational problems that run from HLA typing and antigen processing through peptide-MHC binding, immunogenicity, and TCR specificity to neoantigen identification, tumor T-cell antigen classification, and vaccine design. This monolithic treatment conceals sharp differences in methodological maturity that only a per-sub-task view exposes. An aggregate T-cell count cannot reveal that MHC binding (T3 and T4) and TCR specificity (T6) are now saturated with transformers and protein language models, whereas tumor T-cell antigen classification (T9) and multi-epitope vaccine design (T10) remain dominated by classical machine learning. It likewise cannot reveal that several sub-tasks, in particular HLA typing (T1), structural TCR modelling (T7), and tumor T-cell antigen classification (T9), are not recognised as distinct prediction tasks by any of the seven reviews. The present review is the first to decompose the field into the resulting unified taxonomy of twelve prediction tasks and to analyse every stage of the predictive pipeline across them.

**Table 1:** Scope and limitations of existing epitope prediction review articles.

Review article	Journal/publisher	Databases (linear/ conform./ T-cell)	Predictors (linear/ conform./ T-cell)	Scope	Shortcomings
VillanuevaFlores et al. (32)	npj Vaccines/Sealy Institute for Vaccine Sciences	–/–/–	4/6/9	• Practical guide to AI integration in vaccine development • CNNs, RNNs/LSTMs, transformers, and graph neural networks (GNNs) for epitope prediction • Quantitative tool comparison (AUC, MCC, precision, recall); VenusVaccine case study • Structure-based methods (AlphaFold2); computational– experimental integration	• No systematic database classification or benchmark dataset documentation • Insufficient coverage of traditional methods; no comprehensive tool catalog • Data limitations and bias acknowledged but not systematically addressed • Limited predictor availability analysis (open/closed source, maintenance)
Grewal et al. (30)	Frontiers in Immunology	8/4/10	13/7/15	• Comprehensive ML catalog across 3 epitope prediction tasks • Databases and tools with accessibility assessments • ML integration with peptide arrays and crystallography • SARS-CoV-2 case studies	• No standardized comparative performance analysis across methods • Limited coverage of modern representation learning • No database quality or tool maintenance analysis • Cataloging focus without classifier selection guidance
Bravi et al. (33)	Perspectives Open/npj	1/3/2	4/8/6	• Overview of ML/DL for vaccine target selection (B-cell, T-cell, paratope) • Feature-based to deep learning architectures; interpretable ML • Highlights data quality and representation learning importance	• No systematic benchmark dataset analysis or standardized evaluation protocols • Limited coverage of modern representation learning • No systematic classifier performance comparisons • Data limitations identified as bottleneck without proposed solutions
Guarra et al. (31)	Journal of Chemical Theory and Computation/ACSPublications	1/2/4	2/8/5	• Structural biology approaches for antibody/immunogen design • Brief ML/DL coverage; focus on structure- and physics-based methods • Key database overview; AI– structural biology integration for therapeutics	• Narrow ML/DL coverage; modern methods underrepresented • No systematic database analysis or detailed evaluation settings • Insufficient tool availability and performance comparison
Bukhari et al. (11)	Pathogens/MDPI	1/1/3	5/2/23	• Traditional ML (ANNs, SVMs, decision trees, ensembles) for B/Tcell prediction • Database overview (IEDB, SYFPEITHI); tool categorization with accessibility data • SARS-CoV-2 application with per-study performance figures	• No transformers, protein language models, or GNNs • No systematic benchmark dataset analysis across species • No standardized evaluation framework for cross-study comparison
Jaiswal et al. (29)	Systems and Synthetic Immunology/Springer Nature	1/1/3	2/1/2	• Few traditional ML tools (ANN, SVM, Random Forest) • Basic algorithmic descriptions, tool workflows, and limited performance figures	• No deep learning coverage • No benchmark dataset analysis, evaluation settings, or representation learning
SanchezTrincado et al. (28)	Journal of Immunology Research/Hindawi Publishing Corporation	1/1/3	5/4/12	• Few primary databases and web servers for epitope prediction • Traditional ML and basic feature engineering	• No deep learning or representation learning • No benchmark dataset details (species, evaluation settings) • Missing state-ofthe-art performance figures
Our Work	N/A	9/10/24	32/13/103		
				• First comprehensive analysis unifying Bcell (linear + conformational) and T-cell epitope prediction • First to decompose the field into a unified taxonomy of twelve prediction tasks. The single T-cell column in this table aggregates the ten T-cell subtasks (T1–T10) that **Table 2** resolves individually • Systematic examination of databases, benchmark datasets, and predictive pipelines with evaluation practices and accessibility status • Identifies persistent gaps across a unified five-stage prediction pipeline • Proposes actionable future directions for robust, generalizable, interpretable predictors	• Qualitative literature survey; no independent model re-execution • Coverage bounded by search scope (2015–2026)

A critical analysis of the 7 reviews at the data acquisition level reveals fundamental failures in database classification that prevent researchers from navigating the complex database landscape effectively. Early reviews referenced limited databases with Sanchez-Trincado et al. (28) and Jaiswal et al. (29) covering only a handful of databases each, whereas recent reviews achieved substantially broader coverage with Grewal et al. (30) encompassing the most databases, including specialized repositories (per-review counts in **Table 1**). However, all reviews systematically failed to provide classification frameworks that would distinguish primary epitope databases (IEDB, BciPep) from specialized pathogen-specific databases (HIV, FLAVIdB) or supporting structural databases (PDB, ATLAS). This fundamental classification failure prevents researchers from understanding appropriate database selection criteria for specific prediction contexts. Furthermore, Grewal et al. (30) provides the most comprehensive database coverage but offers no systematic framework for distinguishing database types or usage contexts, while Guarra et al. (31) focuses on structural approaches with limited database coverage without acknowledging sequence-based database categories that support the majority of applications. Notably, Villanueva-Flores et al. (32) emphasizes practical AI tool implementation over systematic database cataloging, and this reflects a shift toward methodology-focused guidance, although it still lacks comprehensive database classification frameworks.

Building upon these database classification inadequacies, a systematic examination of benchmark dataset coverage reveals comprehensive documentation failures that fundamentally limit evaluation resource selection. While Bukhari et al. (11) demonstrates increased attention to pandemic-specific datasets and Bravi et al. (33) explicitly acknowledges dataset limitations as critical bottlenecks, no review provides comprehensive coverage of available benchmark datasets with essential statistical characteristics, species distribution analysis, or systematic accessibility status assessment. Villanueva-Flores et al. (32) acknowledges data limitations and bias as major challenges in AI-based epitope prediction. However, this recognition does not translate into comprehensive benchmark dataset documentation with detailed statistical profiles. This documentation gap becomes critically problematic when considering that benchmark datasets vary significantly in size and accessibility across different epitope types, yet reviews provide no systematic guidance for identifying accessible versus permanently inaccessible benchmarks. Moreover, the absence of comprehensive dataset coverage with detailed statistical profiles and cross-species distribution analysis prevents researchers from making informed decisions about benchmark selection for specific biological contexts or reliable cross-species generalization assessment.

These dataset documentation failures extend systematically to methodological analysis where a critical examination of representation learning coverage reveals categorical inadequacies that prevent evidence-based approach selection. While Bravi et al. (33) acknowledges representation learning importance for immunological predictions and Grewal et al. (30) discusses various feature engineering approaches across prediction tasks, Villanueva-Flores et al. (32) provides the most comprehensive coverage of modern deep learning architectures including CNNs, RNNs/LSTMs, transformer-based models, and graph neural networks (GNNs) with detailed explanations of their applications in epitope prediction. However, despite this architectural coverage, no review provides systematic categorization of the distinct representation learning categories actually used across existing predictors with comprehensive strength and limitation assessment for specific prediction scenarios. Furthermore, this categorization failure becomes particularly problematic when considering that transformer-based embeddings, multi-scale feature integration, and transfer learning approaches demonstrate documented performance improvements over traditional feature engineering methods. But existing reviews provide no systematic framework for understanding when specific representation learning approaches are most appropriate or how they perform comparatively across different epitope types and prediction contexts.

Similarly, an analysis of classifier utilization patterns reveals that reviews provide algorithmic descriptions without systematic examination of classifier usage in existing predictors or comparative performance evaluation across different epitope prediction contexts. Bukhari et al. (11) covers traditional approaches including artificial neural networks, support vector machines, and ensemble methods with extensive T-cell predictor coverage, while Guarra et al. (31) focuses on structure-based computational methods with emphasis on conformational approaches (per-review predictor counts in **Table 1**). Villanueva-Flores et al. (32) advances this analysis by providing quantitative performance benchmarks for state-of-the-art AI tools in **Table 1** of their review, and it systematically compares tools across critical metrics including AUC, accuracy, precision, recall, and Matthews correlation coefficient (MCC). However, despite this valuable performance comparison, no review systematically categorizes which classifiers are actually being deployed across the full spectrum of existing predictors with rigorous performance comparison analysis across diverse application scenarios. This utilization pattern gap prevents researchers from understanding which classifiers perform optimally for specific epitope types under varying data availability conditions or how architectural choices impact generalization to novel pathogens.

These methodological analysis deficiencies culminate in evaluation standardization failures where reviews demonstrate systematic inadequacies in documenting evaluation settings and performance analysis protocols. While Bukhari et al. (11) includes performance metrics from individual studies and Guarra et al. (31) provides some benchmarking studies with emphasis on structural approaches, Villanueva-Flores et al. (32) makes significant progress by providing standardized performance comparisons and acknowledging the need for standardized benchmarking frameworks. However, despite this recognition, no review systematically documents evaluation settings for each benchmark dataset with comprehensive performance analysis of existing predictors that would enable standardized comparison across methods under controlled conditions. This evaluation documentation gap becomes critically problematic when considering that studies employ inconsistent evaluation criteria across multiple benchmark datasets with varied protocols, yet no systematic framework exists for understanding predictor performance patterns across standardized evaluation settings or for distinguishing genuine predictive capability from benchmark overfitting.

Finally, these systematic analytical failures extend comprehensively to practical implementation assessment where reviews provide accessibility information without systematic documentation of predictor availability status and deployment characteristics. Sanchez-Trincado et al. (28) covers available web servers with basic descriptions while Grewal et al. (30) provides systematic accessibility assessments for databases and tools. Villanueva-Flores et al. (32) makes a unique contribution by providing detailed guidance on integrating AI tools into laboratory workflows, including practical considerations for data preparation, computational resources, experimental validation strategies, and best practices for interpreting predictions. However, despite this practical implementation focus, no review systematically documents which predictors are open source versus closed source or provides comprehensive web server availability analysis with detailed deployment infrastructure characteristics and long-term maintenance status. Moreover, this availability documentation gap becomes substantially problematic when considering that predictor accessibility varies significantly across different epitope types, yet researchers lack systematic guidance for identifying deployable tools with appropriate maintenance protocols and long-term sustainability assurance.

These systematic limitations across six critical methodological dimensions demonstrate conclusively that existing reviews provide either basic resource cataloging or methodology-focused practical guidance without the comprehensive analytical frameworks required for evidence-based method selection and successful practical implementation. The field urgently requires systematic integration encompassing rigorous database classification enabling appropriate resource selection, complete benchmark dataset documentation with accessibility status assessment, systematic representation learning categorization with performance evaluation, comprehensive classifier usage analysis with strength assessment, standardized evaluation documentation with predictor performance analysis, and systematic assessment of predictor accessibility and deployment, namely open versus closed source status, web-server availability, and long-term maintenance. To address these comprehensive analytical failures, our review provides the first systematic integration framework that bridges these critical gaps. We present unified classification of 43 dedicated epitope/immunology databases (alongside 5 supporting general-purpose and structural resources) enabling appropriate resource selection, complete benchmark dataset documentation with accessibility assessment covering 144 datasets, systematic representation learning categorization across multiple paradigms, and comprehensive classifier usage analysis examining 148 predictive models across different experimental settings and evaluation measures. Unlike existing reviews that either catalog resources or provide practical methodology guidance, our evidence-based analytical framework provides actionable insights for method selection with comprehensive coverage of databases, benchmarks, and systematic evaluation protocols to enable effective translation of computational predictions into practical immunological applications.

## 5 A unified taxonomy of epitope prediction tasks

Before surveying the databases, datasets, and computational components that populate the predictive pipeline, it is essential to clarify *what* is being predicted, because computational epitope identification is not a single problem but a family of related tasks that together serve one overarching goal, namely the rational design of vaccines, immunotherapies, and immunodiagnostics. At the highest level the field divides into two branches that this review treats throughout, namely B-cell epitope prediction and T-cell epitope prediction. B-cell epitopes are recognised directly by antibodies and B-cell receptors, and B-cell prediction therefore comprises two tasks, namely linear B-cell epitope prediction and conformational B-cell epitope prediction. T-cell recognition is indirect and proceeds through a multi-step antigen-presentation and receptor-recognition cascade. This cascade is itself not monolithic, because it decomposes into a taxonomy of *ten* prediction sub-tasks (T1–T10) that span the full pathway from genotyping of the patient’s HLA, through antigen processing and peptide-MHC presentation, to receptor recognition, immunogenicity, and downstream translational applications. The two B-cell tasks and the ten T-cell sub-tasks together constitute the twelve epitope prediction tasks that this review analyses across the predictive pipeline (**Figure 6**).

**Figure 6:**
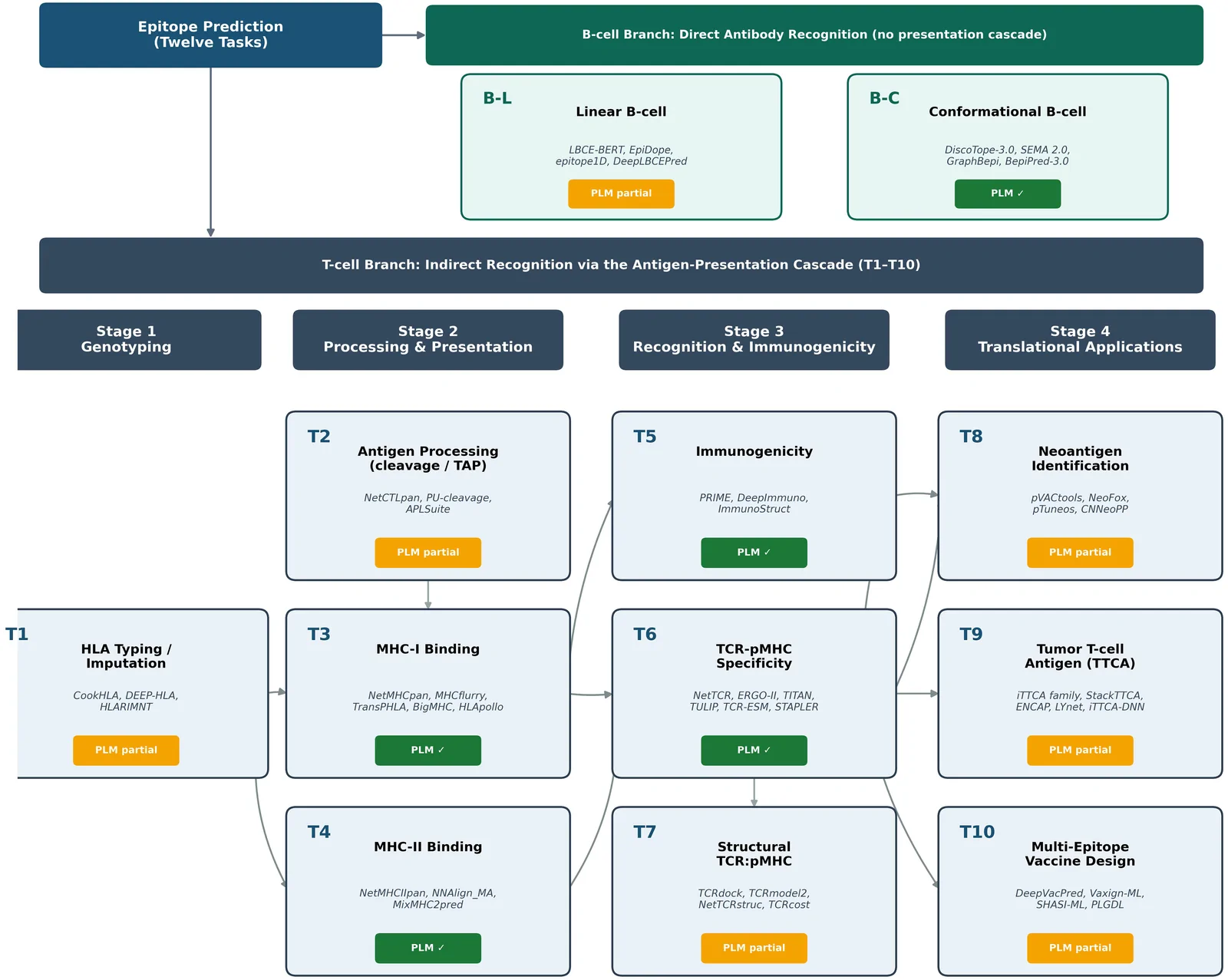
Unified taxonomy of the twelve epitope prediction tasks across the two branches of adaptive immunity. The B-cell branch comprises two tasks that antibodies recognise directly, namely linear and conformational B-cell epitope prediction, and these are parallel rather than sequential because B-cell recognition does not pass through an antigen-presentation cascade. The T-cell branch comprises ten sub-tasks (T1–T10) mapped onto the biological antigen-recognition cascade: upstream genotyping (T1) and antigen processing (T2) feed peptide– MHC presentation (T3/T4), which is evaluated for immunogenicity (T5) and TCR recognition (T6, whose structure is modelled by T7), and these converge in the translational applications of neoantigen identification (T8), tumor antigen classification (T9), and multi-epitope vaccine design (T10). Sub-tasks T1–T7 form the general recognition cascade that applies to any antigen. Sub-tasks T8 and T9 are oncology-specific applications, whereas T10 applies across infectious-disease and cancer settings. Each task box lists representative tools and carries a badge whose colour denotes protein-language-model (PLM) adoption: green for extensive, amber for partial or emerging, and red for none.

The ten T-cell sub-tasks, in the natural order of the recognition cascade, are T1 HLA typing and imputation, T2 antigen processing (proteasomal cleavage and the transporter associated with antigen processing, TAP), T3 MHC class I binding and presentation, T4 MHC class II binding, T5 T-cell epitope immunogenicity, T6 TCR–peptide-MHC (pMHC) binding specificity, T7 structural TCR:pMHC modelling, T8 neoantigen identification, T9 tumor T-cell antigen (TTCA) classification, and T10 multi-epitope vaccine design. These sub-tasks are tightly coupled and feed one another. HLA typing (T1) establishes the patient-specific context. Antigen processing (T2) generates the candidate peptides that binding predictors (T3, T4) score for presentation. Immunogenicity (T5) and TCR specificity (T6), whose structural basis is modelled by T7, determine which presented peptides actually elicit a response. These recognition predictions then converge in the translational applications of neoantigen identification (T8), tumor antigen classification (T9), and multi-epitope vaccine design (T10). This taxonomy classifies the computational prediction problems that make up the field, and it does not imply that every sub-task is run for every antigen. Sub-tasks T1–T7 are antigen-source-agnostic, because they are governed by the general biology of antigen presentation and T-cell recognition. They apply whether the source protein is viral, bacterial, parasitic, allergenic, self, or tumor-derived. The translational applications differ in scope. Neoantigen identification (T8) and tumor T-cell antigen classification (T9) are specific to the oncology setting. A neoantigen is by definition a tumor-specific peptide that arises from somatic mutation, and a tumor T-cell antigen is defined by its association with the tumor. Neither task therefore applies to the epitopes of a viral, bacterial, or other non-tumor antigen. Multi-epitope vaccine design (T10) is broader, because it applies across both infectious-disease and cancer settings. For a non-cancer antigen, the recognition cascade runs T1–T7 but does not enter T8 or T9. It reaches T10 only when a vaccine is the goal. We retain the two oncology-specific sub-tasks because they are among the largest and most clinically consequential application areas of computational T-cell prediction, and a complete review of the field cannot omit them. Recognising this structure is important for two reasons. First, T-cell epitope prediction cannot be judged from any single sub-task, because the degree of methodological development varies sharply along the cascade. The tumor T-cell antigen sub-task (T9) was historically dominated by classical machine learning, and deep learning has only recently emerged there through iTTCA-DNN and LYnet, whereas the most active deep learning and protein language model (PLM) development resides in the other sub-tasks, particularly MHC binding (T3 and T4), immunogenicity (T5), TCR specificity (T6), and structural modelling (T7). A balanced assessment of the field therefore requires the complete taxonomy. Second, the taxonomy provides the organising frame for the remainder of this review. The subsequent sections analyse the databases, datasets, representation learning, classifiers, language models, performance, and accessibility *across* these tasks, and **Table 2** previews the per-task picture that those sections substantiate in detail. We therefore introduce the twelve tasks here, and we begin with the two B-cell tasks before proceeding through the ten T-cell sub-tasks in their natural biological order along the recognition cascade.

**Table 2:** Consolidated per-task summary of the twelve epitope prediction tasks across the predictive pipeline: the two B-cell tasks, namely linear and conformational B-cell prediction, and the ten T-cell sub-tasks (T1–T10).

Sub-task	Representative tools	Key databases	Dominant method	Best AUC/acc.*	PLM use	Code	Principal gap
Linear B- cell	LBCE-BERT (8), LBCE-XGB (10), EpiDope (34), epitope1D (9), DeepLBCEPred (7), EpiBERTope (35)	IEDB, Bcipep, AbYbank/AbDb	ML (RF/SVM/XGBoost), CNN–BiLSTM, BERT	0.53– 0.99 AUC	Yes (BERT, ESM)	Med	cross-dataset collapse; overfitting; performance plateau
Conformational B-cell	DiscoTope3.0 (36), SEMA 2.0 (15), GraphBepi (37), BepiPred-3.0 (38), EpiCluster (6), CBTOPE2 (39)	PDB, SAbDab, IEDB-3D	GNN, MLP/CNN, ESM/AF2 structural	<0.6– 0.99 AUC	Yes (ESM, AF2)	High	poor neutralbenchmark generalisation (Cia); datasetdependent
T1 HLA typing	CookHLA (40), DEEP-HLA (41), HLARIMNT (42) (impute.); HLA-HD (43), HISATgenotype (44) (type)	IPD- IMGT/HLA, 1000 Genomes, dbSNP	HMM, CNN, transformer; alignment, EM	93–99% conc.	Emerging	Med	non-European bias; class-II harder
T2 Antigen processing	NetCTLpan (45), NetCleave (46), PU-cleavage (47), APLSuite (48)	IEDB, MHC Ligand Atlas, PDB	feed-forward/MLP/BiLSTM	∼0.87– 0.88 AUC	Emerging (ESM2)	Med	smallest task; TAP frozen; ceiling
T3 MHC-I binding	NetMHCpan- 4.1 (49), MHCflurry2.0 (50), TransPHLA (51), BigMHC (52), BVLSTMMHC (53), DPCMHC (54), 3pHLA (55), MixMHCpred (56), MUNIS (57)	IEDB, NetMHCpan corpus, SysteMHC Atlas	ensemble NN/CNN/trans- former	AUC >0.9	Yes (ESM, BERT)	High	MS data siloed; PLM not yet Standard
T4 MHC- II binding	NetMHCIIpan- 4.0 (49), NNAlign MA (58), MixMHC2pred (59), DeepSeqPanII (60), MTL4MHC2 (61), BERTMHC (62)	IEDB, NetMHCII corpus, immunopeptidomics	NNAlign /LSTM/ transformer	AUC 0.8–0.9	Yes (ESM2, BERT)	High	DQ/DP underrepresented; lags MHC-I
T5 Immuno- genicity	PRIME (63), DeepImmuno (64), Repitope (65), ImmunoStruct (66), DeepHLApan (67)	IEDB, TANTIGEN, ITSNdb	RF/XGBoost, CNN, GNN, transformer	AUC 0.6–0.8	Partial (transformer)	High	AUC plateau; weak Negatives
T6 TCR– pMHC specificity	NetTCR- 2.0/2.1 (68, 69), ERGO-II (70), TITAN (71), epiTCR (72), TULIP (73), TCR-ESM (74), MixTCRpred (75), UniPMT (76), UnifyImmun (77), STAPLER (78), catELMo (79)	VDJdb, McPAS- TCR, 10x, ImmuneCODE, IMMREP	CNN, LSTM, attention, GNN, trans- former LM	AUC 0.6– 0.98	Yes (extensive)	High	unseenepitope generalisa- tion; data bias
T7 Structural TCR	TCRdock (80), TCRmodel2 (20), NetTCRstruc (81), TCRcost (82), ImmuneBuilder (83) AlphaFold3 (84)	PDB, ATLAS, TCR3d, STCRDab ,	AlphaFold2/3, GVP-GNN, CNN+LSTM	AUC- ROC 0.76– 0.97	AF2/AF3, ESM-IF1	High	scarce structures; compute cost
T8 Neoantigen ID	pTuneos (85), NeoGuider (86), TruNeo (87), CNNeoPP (88), NeoPredPipe (89), NeoFox (90), pVACview (91)	TCGA, IEDB, COSMIC, dbSNP, TESLA	rule-based pipelines, RF/XG- Boost, logistic reg., deep NN+BERT	AUC 0.81– 0.99	Partial	High	weak immunogenicity filter; non-SNV
T9 TTCA	iTTCA- RF (92)/MFF (93)/M PSRTTCA (95), StackTTCA (96), ENCAP (97), iTTCA-DNN (98), LYnet (99)	TANTIGEN, VLCAPD (94),, IEDB	ML (SVM/R- F/ET), ensemble, emerging DL (DNN, CNN–BiLSTM)	Acc. 0.72– 0.99	Emerging	High	DL just emerging; PLM only via general frameworks; 5 benchmark Sets
T10 Vac- cine design	DeepVacPred (100) Vaxign-ML (101), SHASI-ML (102), PLGDL (103)	,IEDB, Protegen, VaxiJen/Vaxign, proteomes	XGBoost, DNN (CNN+dense), PLM+GNN	∼0.85– 0.97 in-silico	Emerging	Med	few endto-end ML; no wet-lab efficacy

Among these, sub-tasks T1–T7 apply to the epitopes of any antigen. Sub-tasks T8 and T9 are oncology-specific applications, whereas T10 applies across infectious-disease and cancer settings.

*The *Best AUC/acc.* column lists illustrative best-reported figures drawn from heterogeneous datasets, splits, and evaluation protocols. These figures are *not* directly comparable across studies or sub-tasks and are provided only to indicate the approximate maturity of each sub-task. Underlying per-study values are listed in **Supplementary Table 4**. PLM, protein language model.

### 5.1 The B-cell branch: direct antibody recognition

Antibodies and B-cell receptors bind their target epitopes directly on the surface of a folded antigen, so B-cell prediction does not pass through an antigen-presentation cascade. It instead comprises two parallel tasks that differ in how the epitope is defined along the antigen.

### 5.2 Linear B-cell epitope prediction

A linear, or continuous, B-cell epitope is a short contiguous stretch of amino acids, typically 5 to 20 residues, that an antibody or B-cell receptor recognises directly on an antigen. Because the determinant is contiguous in sequence, the task is amenable to sequence-based modelling, and it is therefore the most tractable B-cell task and the usual entry point for new encodings and classifiers. Its significance is practical, because short synthetic peptides that reproduce a linear epitope underpin peptide-based vaccines, serological diagnostics, and antibody development, so reliable linear prediction directly accelerates these applications. It is the sequence-level counterpart of conformational B-cell prediction, and it connects to the T-cell branch through the helper-T-cell collaboration that B cells require, because high-affinity antibody production depends on CD4^+^ help that is itself governed by MHC class II presentation (T4). A predicted epitope must be surface-accessible on the native antigen to be engaged by a circulating antibody. The central goal is therefore to separate the minority of antibody-binding residues from the bulk of the antigen sequence and to do so in a way that generalises across antigens rather than memorising a single benchmark.

### 5.3 Conformational B-cell epitope prediction

A conformational, or discontinuous, B-cell epitope is a cluster of amino acids that lie far apart in the linear sequence but are brought into spatial proximity by the antigen’s three-dimensional fold, and such epitopes typically span clusters of roughly 20 to 70 residues that jointly form the antibodybinding surface. The great majority of native B-cell epitopes are conformational, so accurate prediction here is essential for structure-based vaccine design and therapeutic-antibody development against correctly folded targets. Because the determinant is defined by the folded structure rather than by sequence alone, the task requires explicit structural modelling, and it increasingly relies on graph neural networks together with protein-language-model and AlphaFold-derived structural embeddings. It is the structural counterpart of linear B-cell prediction and shares the same downstream applications, and like its linear counterpart it ultimately depends on CD4^+^ helper-T-cell support modelled in the T-cell branch. The residues must be surface-exposed on the assembled structure to be accessible to an antibody. Its defining challenge is robustness, because predictors that score highly within individual studies degrade sharply on neutral structure-based benchmarks, so generalisation across antigens rather than peak in-study performance is the central open goal. The T-cell branch: the antigen-recognition cascade. T-cell recognition is indirect, and the ten sub-tasks below are presented in the natural order of the antigen-presentation and receptorrecognition cascade.

Stage 1: Upstream genotyping. The cascade begins by establishing the patient-specific HLA context against which all downstream presentation and recognition are evaluated.

### 5.4 T1: HLA typing/imputation

T1 is the upstream genotyping step of the cascade, and it establishes which HLA alleles a patient carries. The HLA genes encode the MHC molecules that present peptides to T cells, and they are the most polymorphic genes in the human genome, with tens of thousands of alleles catalogued. Because every class-I and class-II presentation prediction is allele-specific, T1 fixes the patient-specific context on which all downstream sub-tasks depend, which makes it foundational for personalised vaccine and immunotherapy design. Its central importance lies less in novel modelling than in accuracy and population equity, because errors or ancestry bias introduced here propagate into every subsequent step of the pipeline.

Two distinct computational strategies determine these alleles, and this review treats them as separate paradigms. The first is *HLA typing* from sequencing reads, in which whole-exome, whole-genome, or RNA sequencing reads are matched against a reference of known alleles to call the genotype directly. The tools that perform it are predominantly statistical or algorithmic rather than machine learning. HLA-HD scores weighted read counts against an allele dictionary, HLAminer parses direct long-read sequence alignments, and HISAT-genotype combines a graphbased index of the reference genome and its known variants with an expectation-maximisation algorithm that estimates allele abundance. The second strategy is *HLA imputation*, in which the alleles are predicted from the inexpensive single-nucleotide-polymorphism (SNP) genotypes that microarrays produce in large genome-wide association studies. Imputation exploits linkage disequilibrium, namely the non-random co-occurrence of particular SNPs with particular HLA alleles, so that the unobserved alleles can be inferred from the observed SNP pattern. This second strategy is where machine learning and deep learning have entered the task. Alongside classical maximum-likelihood imputation such as HaploSFHI, recent predictors apply an upgraded hidden Markov model that adaptively learns the local genetic map (CookHLA), a multitask convolutional neural network (DEEP-HLA), and a Transformer that reads the chunked SNP sequence through an attention mechanism (HLARIMNT). The methodological frontier of T1 therefore lies in imputation, whereas read-based typing remains the established and largely non-machine-learning production route. Because this review concentrates on machine learning and deep learning, the three imputation models are catalogued as the T1 predictors in the sections that follow, and the statistical and alignment-based typing tools are noted here as the complementary non-machine-learning paradigm.

Stage 2: Antigen processing and peptide-MHC presentation. The processing machinery generates candidate ligands (T2), which are then scored for presentation by class-I (T3) and class-II (T4) molecules.

### 5.5 T2: antigen processing (proteasomal cleavage/TAP)

T2 predicts antigen processing, which is the set of intracellular events that cut a full-length protein into the short peptides that MHC molecules can display, and it therefore determines which fragments are even available for presentation. For the class I pathway the dominant event is proteasomal cleavage. The proteasome is the cell’s principal protein-degradation machine, and it chops cytosolic proteins into fragments whose carboxy-terminal (C-terminal) end usually defines the final class I ligand. These fragments are then moved from the cytosol into the endoplasmic reticulum by the transporter associated with antigen processing (TAP), where they are loaded onto MHC class I. A predictor for T2 reproduces this chain by scoring how likely each residue is to be a cleavage site and how efficiently a peptide is transported, and it is normally placed ahead of binding prediction so that the two steps together represent the full class I presentation pathway. The class II pathway is handled differently, because proteins are degraded inside endolysosomal compartments rather than by the cytosolic proteasome, and there the local conformational stability of the antigen governs which regions survive to be presented to CD4^+^ T cells. Conceptually T2 sits immediately upstream of MHC binding (T3 and T4), because the peptides that binding predictors score must first be generated by processing. It is the smallest and most neglected sub-task, and TAP transport in particular has been modelled only sparingly, yet improving it tightens the candidate pool that the remainder of the cascade evaluates.

### 5.6 T3: MHC class I binding and presentation

T3, the most mature sub-task, predicts whether a short 8–11-mer peptide binds to and is presented by an MHC class I molecule, expressed either as binding affinity or as mass-spectrometry elutedligand presentation. It is the central node of class-I antigen presentation, because it consumes the ligands generated by processing (T2) within the allele context fixed by typing (T1), and its predicted binders are the substrate that immunogenicity (T5), TCR specificity (T6), and the neoantigen and vaccine applications (T8, T10) build upon. Its relative maturity makes it the de-facto backbone of most T-cell prediction pipelines.

### 5.7 T4: MHC class II binding

T4 predicts the presentation of longer, variable-length peptides (approximately 13–25-mers) by HLA-DR, -DQ, and -DP, together with the location of the 9-mer binding core, and it thereby governs CD4^+^ helper-T-cell responses. It is the class II counterpart of T3 and shares the same downstream consumers, but it is intrinsically harder because the binding register is open-ended and the training data are sparser. Reliable T4 prediction matters most wherever helper-T-cell coverage is required, in particular for multi-epitope vaccine design (T10).

Stage 3: Immunogenicity and TCR recognition. Presented peptides are evaluated for their capacity to elicit a response (T5) and for recognition by the T-cell receptor (T6), whose threedimensional basis is modelled structurally (T7).

### 5.8 T5: T-cell epitope immunogenicity

T5 asks a stricter question than binding: it predicts whether a peptide that is presented on an MHC molecule will actually *elicit* a T-cell response. This distinction is central, because the two properties diverge sharply in practice. Only a small fraction of presented binders are ever engaged by a T-cell receptor, so a high-affinity binder identified by T3 or T4 is not necessarily immunogenic. Immunogenicity depends on determinants that binding models do not represent. The peptide must differ sufficiently from the self-peptides to which the T-cell repertoire was rendered tolerant, so dissimilarity-to-self, or foreignness, is a primary driver, and the residues that project away from the MHC groove toward the receptor must be compatible with an available TCR. The chemical character of the central, receptor-exposed positions is therefore especially influential (104). T5 consequently acts as the decisive filter that reduces the many predicted binders to the minority that are functionally relevant, and it is the pivotal input to neoantigen prioritisation (T8) and rational vaccine design (T10). Two factors make it the hardest and most rate-limiting sub-task. First, the biological signal is weak and depends on many interacting properties. Second, trustworthy negative data are scarce, because a peptide that has not been observed to trigger a response may be truly non-immunogenic or may simply never have been tested in the appropriate HLA context, and this shortage of reliable negatives both inflates apparent accuracy and depresses real-world performance. Most predictors address class I, CD8^+^ immunogenicity, whereas class II, CD4^+^ immunogenicity remains comparatively underdeveloped. For these reasons the accuracy ceiling reached at T5 propagates into every downstream application that depends on it.

### 5.9 T6: TCR–peptide-MHC binding specificity

T6 predicts whether a particular T-cell receptor (TCR) recognises a given peptide-MHC complex. The TCR is the membrane receptor through which each T cell reads presented peptides, and its specificity is determined mainly by three hypervariable loops, the complementarity-determining regions CDR1, CDR2, and above all CDR3, that are carried on its paired *α* and *β* chains. Because these loops are assembled by somatic V(D)J gene recombination, the resulting repertoire is astronomically diverse, and the number of distinct receptors available to an individual is commonly estimated between 10^15^ and 10^61^. This binding event is the molecular switch of cellular adaptive immunity, because it is the point at which a peptide that has survived processing and presentation is finally either recognised or ignored. The same diversity that makes recognition specific also makes it the central computational difficulty: the space of possible TCR–peptide pairs is far too large to characterise experimentally, so a predictor must generalise from a small and biased sample of measured pairs to receptors and epitopes that it has never encountered. For this reason T6 is the most immunologically nuanced sub-task, and it is also the locus in which modern deep learning and protein language models are most heavily concentrated, so an accurate picture of the field cannot be formed without it. It complements immunogenicity (T5) by supplying the receptor-side view of recognition, it is the sequence-level counterpart of the structural modelling addressed in T7, and its predictions underpin repertoire-based diagnostics, adoptive and engineered TCR-T cell therapies, and neoantigen vaccine design. Its defining open problem is generalisation to *unseen* epitopes, and this difficulty is compounded by training data that are dominated by a few HLA alleles and by the scarcity of experimentally confirmed non-binding pairs.

### 5.10 T7: structural TCR:pMHC prediction

T7 predicts the three-dimensional structure of the TCR:peptide-MHC complex and uses that structure to judge recognition. It addresses the same biological event as T6, namely whether a given receptor engages a given peptide-MHC, but it reasons from physical geometry rather than from sequence patterns alone. The motivation is that recognition is ultimately governed by how the atoms of the receptor and the presented ligand are arranged and contact one another in space, so sequence-only predictors eventually reach a performance ceiling that an explicit structural model can move beyond. The task therefore has two coupled computational parts. The first part is structural modelling, in which the amino acid sequences of the TCR and the peptide-MHC are folded and docked into a single predicted complex that specifies the docking orientation, the conformations of the hypervariable complementarity-determining region (CDR) loops, and the atom-level interface contacts. The second part is recognition scoring, in which the quality of the modelled interface is evaluated so that true binders are separated from decoy peptides or non-binding receptors. The defining obstacle is data scarcity, because only a few hundred TCR:peptide-MHC complexes have been solved experimentally and they are dominated by class I, so there is far too little structural data to train large models from scratch, and most methods consequently adapt the AlphaFold protein-structure framework rather than learn folding and docking afresh. T7 is best understood as the structural counterpart of the sequence-based T6. It supplies orthogonal and mechanistically interpretable evidence, it can validate or refine sequence-level predictions, and its contribution is greatest where structural interpretability matters more than high throughput. Its principal open problems are the shortage of experimental structures, the heavy computational cost of structure-based pipelines, and the still-immature integration of structural and sequence-based recognition.

Stage 4: Translational applications. The recognition predictions converge in clinical and designoriented applications, namely neoantigen identification (T8), tumor T-cell antigen classification (T9), and multi-epitope vaccine design (T10). Two of these applications are specific to the oncology setting. Neoantigen identification (T8) and tumor T-cell antigen classification (T9) are entered only for tumor antigens, whereas multi-epitope vaccine design (T10) applies to infectious-disease and cancer targets alike.

### 5.11 T8: neoantigen identification

Neoantigens are tumor-specific peptides that arise from somatic alterations in cancer cells, including non-synonymous point mutations, small insertions and deletions, gene fusions, and aberrant alternative splicing. The wild-type counterparts of these sequences are absent from normal tissue, so the T-cell repertoire was never rendered tolerant to them, and they are therefore among the most attractive targets for personalised cancer immunotherapy, namely individualised cancer vaccines and adoptive or engineered T-cell therapies. T8 is an integrative *applied pipeline* rather than a single predictor. It first calls somatic mutations from tumor and matched-normal whole-exome or whole-genome sequencing and quantifies expression from RNA-seq, it then translates the affected transcripts into candidate mutant peptides, and it finally chains the upstream sub-tasks, namely HLA typing (T1), antigen processing (T2), class I and class II binding (T3 and T4), and immunogenicity (T5), to rank the candidates that are most likely to be recognised. It is therefore a downstream consumer that inherits both the strengths and the limitations of those upstream sub-tasks, and its dominant bottleneck is the immunogenicity filter (T5) together with the scarcity of experimentally validated positive examples.

Three complementary computational framings recur within this task, and distinguishing them clarifies what each tool actually contributes. The first and largest framing applies machine learning or deep learning to *predict and prioritise* which tumor-specific mutations are immunogenic, and it includes the random-forest prioritiser pTuneos, the logistic-regression model NeoGuider that is built on adaptive kernel-density and isotonic-regression feature transforms, the deep neural pipelines TruNeo and CNNeoPP, the alternative-splicing pipeline ASNP, and the recognitionpotential pipeline NeoPredPipe. The second framing does not predict candidates *de novo* but instead helps researchers interpret the large and complex output of these pipelines: NeoFox annotates each candidate with a comprehensive set of biological features, and pVACview supplies an interactive interface for exploring, comparing, and manually selecting candidates across the variant, transcript, and peptide levels. The third framing inverts the target entirely, because rather than identifying the neoantigens it identifies the neoantigen-*reactive* CD8^+^ T cells that recognise them, as in the gradient-boosting model of Shi et al. that classifies tumor-infiltrating lymphocytes from their single-cell transcriptomes. These three framings address different facets of one clinical problem, and together they supply the prediction, annotation, and cellular-readout infrastructure that personalised cancer immunotherapy requires.

### 5.12 T9: tumor T-cell antigen classification

T9 determines whether a peptide is a tumor-associated antigen recognised by T cells. It is best understood as one translational classification node within the cascade rather than as the whole of T-cell prediction. We adopt T9 as the recurring worked example of this review, and it reappears in each subsequent dimension for three reasons. It is the most fully characterised sub-task, because it has the most complete set of public benchmark datasets and is the only sub-task for which a full deployment and accessibility audit is possible. It is the clearest illustration of the field-wide methodological lag, because its predictors still rely almost exclusively on classical machine learning while modern representation learning and protein language models have only recently begun to appear. Finally, it is a clinically consequential translational application, because its predictions feed cancer-vaccine and immunotherapy design. Its databases, datasets, classifiers, performance, and code availability are therefore analysed in detail as a connected thread across the corresponding later sections.

### 5.13 T10: multi-epitope vaccine design

T10 is the most downstream and integrative application in the cascade, because it converts the upstream recognition predictions into a concrete vaccine candidate. It consumes the outputs of MHC binding (T3 and T4), immunogenicity (T5), and HLA typing (T1), so it sits at the convergence of the whole pipeline and is the point at which upstream prediction quality is translated into a candidate immunogen. Computational approaches to this task fall into two distinct framings, and separating them clarifies what each one contributes. The first framing is multi-epitope construct assembly. Its goal is to select individual B-cell, helper-T-cell, and cytotoxic-T-cell epitopes and to join them, together with short linker peptides and an adjuvant sequence, into a single synthetic protein that is designed to maximise population coverage across the diverse HLA alleles of a target population. DeepVacPred is the representative deep-learning example, because it scores candidate vaccine subunits and assembles the highest-ranked epitopes into one construct in a SARS-CoV-2 case study. The second framing is reverse-vaccinology antigen discovery. Here the unit of prediction is the whole protein rather than the short epitope, and a classifier scans an entire pathogen proteome to rank which proteins are protective antigens worth carrying forward to epitope mapping. Vaxign-ML applies this strategy to viral and bacterial proteomes through a protective-antigenicity (protegenicity) score, and SHASI-ML applies it to *Salmonella* proteins for bacterial vaccine development. This task matters because it is where epitope prediction meets translational immunology, yet, alongside tumor T-cell antigen classification (T9), it remains among the sub-tasks least penetrated by modern representation learning. Its predictors are still dominated by classical machine learning and reverse-vaccinology feature engineering, and protein language models have only very recently appeared, as in the PLGDL framework that combines a protein language model with geometric deep learning to rank protective antigens (103). Its defining open challenges are twofold. Construct-level decisions, namely epitope ordering, linker choice, and adjuvant selection, together with population-level coverage optimisation, remain only weakly modelled, and validation is almost entirely in-silico, so that strong simulated scores do not yet guarantee wet-lab efficacy.

## 6 Epitope prediction databases landscape analysis

Our comprehensive analysis of 155 studies identifies 48 databases and database portals, summarized in **Table 3**, used for linear B-cell, conformational B-cell, and T-cell epitope prediction research. To make the nature of each resource explicit, we use five categories: (1) *Primary*, namely dedicated epitope/immunology databases providing broad-context, experimentally verified epitope or receptor annotations (e.g. IEDB, Bcipep, VDJdb, IPD-IMGT/HLA); (2) *Specialized*, namely disease- or antigen-specific immunology resources (e.g. SDAP, FLAVIdB, ImmuneCODE); (3) *Supplementary*, namely auxiliary antibody, immunogenetics, and repertoire databases (e.g. AbYbank/AbDb, IMGT, TCRdb, PIRD); (4) *Structural repository*, namely 3D structural databases used as source material for conformational and structural-TCR dataset construction (PDB, STCRDab); and (5) *Generalpurpose*, namely broad-scope sequence databases not specific to immunology, included because they are routinely used to build negative sets and retrieve antigen sequences (UniProt, NCBI GenBank, SwissProt). The first three categories constitute the *43 dedicated epitope, receptor, or immunology resources*, and these are supported by *3 general-purpose sequence repositories* and *2 structural repositories (PDB and STCRDab)*. The catalogue includes the canonical T-cell-receptor and HLA databases that anchor modern T-cell prediction, namely VDJdb and McPAS-TCR (TCR– peptide-MHC specificity, T6), IPD-IMGT/HLA (HLA typing, T1), STCRDab (structural TCR, T7), and the 10x Genomics single-cell and ImmuneCODE repertoire resources (T6), together with the immunogenicity and neoantigen resources ITSNdb, dbPepNeo, and NEPdb (T-cell immunogenicity, T5).

**Table 3:** Comprehensive summary of B-cell and T-cell epitopes associated databases, with usage status reflecting frequency and specific context of use in the research community.

#	Name	Category	Scope	Approx. size	Accessibility (2026)	Usage status (context)
Linear B-cell epitope databases						
1	IEDB	Primary	Linear B-cell & T-cell epitopes, multi-species	1.6M epitopes (6.8M assays)	Active	Widely used (general immunology, benchmarking, ML training)
2	Bcipep	Primary	Linear B-cell epitopes	3,031 entries	Outdated updates (no since 2005)	Declining use (historical benchmarking, limited by HIV-centric content)
3	AbYbank/ AbDb	Supplementary	Antibody sequences, structures from PDB	1,500+ structures	Active	Moderately used (antibody structure studies, not epitope-specific)
4	HIV Molecular Immunology Database	Specialized	HIV-associated linear T-cell & Bcell epitopes	13,700 (T-cell), 3,000+ (B-cell)	Active	Widely used (HIV vaccine and immunology research)
5	SDAP	Specialized	Allergen-associated linear B-cell epitopes	4,000+ entries	Active	Specialized use (allergy/allergenicity studies)
6	EPITOME	Specialized	Linear B-cell epitopes	142 entries	Limited activity	Underutilized (small-scale, method validation)
7	AntiJen	Specialized	Linear T-cell & B-cell epitopes, kinetics	24,000 entries	Active	Underutilized (historical, kinetic/thermodynamic studies)
8	CEDAR	Specialized	Cancer-associated linear T-cell & B-cell epitopes	Entry count under development	Active (late alpha)	Emerging use (cancer immunology, neoepitope discovery)
9	FLAVIdB	Specialized	Flavivirus linear B-cell & T-cell epitopes, antigens	12,858 sequences	Inactive	Underutilized (pathogen-specific, flavivirus research)
10	UniProt	General-purpose	Protein sequences, functional annotation	>250M sequences	Active	Widely used (reference for protein annotation, mapping)
11	NCBI GenBank	General-purpose	Nucleotide/protein sequences, reference genomes	>3.7B nucleotide sequences	Active	Widely used (reference for sequence retrieval, mapping)
12	SwissProt	General-purpose	Curated protein sequences, functional annotation	>570,000 entries	Active	Widely used (high-quality reference, annotation)
Conformational B-cell epitope databases						
13	IEDB	Primary	Conformational B-cell & T-cell epitopes, multi-species	1.6M epitopes (6.8M assays)	Active	Widely used (general immunology, 3D/conformational studies)
14	PDB (RCSB PDB)	Structural repository	3D structures of proteins/antibody-antigen complexes, accessed via the RCSB PDB portal (rcsb.org), one of three wwPDB deposition sites	>200,000 structures	Active	Widely used (structural bioinformatics, conformational prediction)
15	SAbDab	Primary	Structural antibody database, antibody-antigen complexes	5,426+ structures	Active	Moderately used (antibody-antigen structure, paratope/epitope mapping)
16	AbYbank/AbDb	Supplementary	Antibody structures from PDB	1,500+ structures	Active	Moderately used (antibody structure, not epitope-specific)
17	HIV Molecular Immunology Database	Specialized	HIV-associated conformational T-cell & B-cell epitopes	13,700 (T-cell), 3,000+ (B-cell)	Active	Widely used (HIV vaccine and immunology research)
18	CED	Specialized	Conformational B-cell epitopes	225 entries	Inactive (not accessible)	Underutilized (historical, conformational epitope studies)
19	HLA Eplet Registry	Specialized	Antibody-verified and predicted eplets on HLA	200+ eplets	Active	Specialized use (transplantation, HLA antibody research)
20	CEDAR	Specialized	Cancer-associated conformational T-cell & B-cell epitopes	Entry count under development	Active (late alpha)	Emerging use (cancer immunology, neoepitope discovery)
21	FLAVIdB	Specialized	Flavivirus conformational B-cell & T-cell epitopes	12,858 sequences	Inactive	Underutilized (pathogen-specific, flavivirus research)
22	IMGT	Supplementary	Immunogenetics, antibody/TCR sequences, 3D structures	>250,000 entries across databases	Active	Widely used (reference for immunogenetics, annotation)
23	IEDB-3D	Supplementary	Structural T-cell & B-cell epitopes, visualization	4,859 assays (B-cell)	Active	Widely used (structural epitope visualization, benchmarking)
T-cell epitope databases						
24	IEDB	Primary	T/B-cell epitopes, MHC data, infectious, allergic, autoimmune, transplant	1.6M epitopes (6.8M assays)	Active (2026)	Widely used
25	SYFPEITHI	Primary	MHC class I/II ligands, peptide motifs (multiple species)	>7,000 peptides	Outdated (since 2012)	Underutilized (historical reference only)
26	MHCBN 4.0	Primary	T-cell epitopes, MHC binding, non-binders	∼25,000 entries	Outdated (2012)	Underutilized (historical reference)
27	EPIMHC	Primary	MHC-restricted peptide ligands and epitopes	480 entries	Inactive	Underutilized (historical reference)
28	SysteMHC Atlas	Primary	MHC-bound peptides (class I & II), immunopeptidomics	∼2.1M unique pep- tides	Active	Underutilized
29	TANTIGEN 2.0	Specialized	Tumor antigens, MHC binding, cancer epitopes	4,296 antigen variants, 403 tumor antigens	Active	Widely used (cancer immunotherapy)
30	CAPD	Specialized	Cancer antigen peptides, MHC	Entry count not speci- fied	Active	Widely used (cancer antigen discovery)
31	CEDAR	Specialized	Cancer epitopes	Entry count under development	Active (late alpha)	Emerging use (cancer immunology)
32	HIV Mol. Imm. DB	Specialized	HIV-specific T/B-cell epitopes, MHC data	13,700 (T-cell), 3,000+ (B-cell)	Active	Widely used (HIV vaccine research)
33	FLAVIdB	Specialized	Flavivirus B-cell and T-cell epitopes, antigens	184 T-cell, 201 B-cell epitopes, 12,858 antigen sequences	Inactive (as of 2026)	Underutilized (pathogen-specific)
35	TCRdb	Supplementary	TCR sequences, immune repertoire	>277M TCR sequences	Active	Moderately used (immune repertoire studies)
36	IMGT/ 3Dstructure-DB	Supplementary	3D structures (immunoglobulins, TCRs, MHC)	Part of larger IMGT system	Active	Moderately used (structural immunology)
37	VDJdb	Primary	Curated TCR–peptide-MHC specificity pairs (sub-task T6)	>90,000 TCR–epitope pairs	Active	Widely used (TCR specificity prediction)
38	McPAS-TCR	Primary	Pathology-associated TCR sequences with epitope/MHC annotation (T6)	Thousands of curated TCRs	Active	Widely used (TCR specificity, disease association)
39	IPD-IMGT/HLA	Primary	Reference HLA allele sequences for typing/imputation (T1)	>40,000 alleles	Active	Widely used (HLA typing, allele reference)
40	STCRDab	Structural repository	Curated TCR and TCR–pMHC 3D structures (T7)	∼900+ structures	Active	Moderately used (structural TCR modelling)
41	10x Genomics dextramer	Specialized	Single-cell paired αβ TCR with peptide specificity (T6)	Paired-chain repertoires	Active	Widely used (TCR specificity training data)
42	ImmuneCODE	Specialized	SARS-CoV-2-associated TCR repertoire and specificity (T6)	Millions of TCR sequences	Active	Moderately used (SARS-CoV-2 TCR studies)
43	Protegen (105)	Specialized	Curated protective antigens for reverse-vaccinology/multi-epitope vaccine design (T10)	Hundreds of protective antigens	Active	Specialized use (reverse vaccinology, protective-antigen reference)
44	ITSNdb	Specialized	Curated immunogenic/nonimmunogenic neoepitope test set for T-cell immunogenicity benchmarking (T5)	Hundreds of validated neoepitopes	Active	Underutilized (immunogenicity benchmarking)
45	dbPepNeo 2.0	Specialized	Human tumour neoantigen peptides with HLA-I presentation and immunogenicity labels (T5/T8)	>22,000 neoantigens	Active	Moderately used (neoantigen immunogenicity)
46	NEPdb	Specialized	Experimentally validated immunogenic and nonimmunogenic neoepitopes (T5/T8)	∼17,000 entries	Active	Moderately used (neoantigen immunogenicity)
47	TumorAgDB2.0	Specialized	Integrated human tumour neoantigen resource (aggregates IEDB, TESLA, CADv1.0, NCI) with immunogenicity labels (T5)	∼10,000 labelled Neoantigens	Active	Emerging use (neoantigen immunogenicity)
48	TBAdb	Supplementary	Curated TCR/BCR–antigen binding pairs, the antigenbinding module of PIRD (T6)	Thousands of receptor–antigen pairs	Active	Moderately used (TCR specificity training data)
49	PIRD	Supplementary	Pan immune repertoire database of curated TCR and BCR sequences (T6)	>1.9M receptor sequences	Active	Moderately used (immune repertoire studies)

To complement this category-level classification, it is useful to map the catalogue onto the twelve prediction tasks in the order used throughout this review, because database selection is ultimately task-driven. *Linear B-cell* prediction draws principally on IEDB together with the dedicated B-cell repositories Bcipep and AbYbank/AbDb, with general-purpose sequence sets (UniProt, NCBI GenBank, SwissProt) supplying negative examples. *Conformational B-cell* prediction is structure-driven and is built principally on the Protein Data Bank (PDB), supplemented by IEDB-derived antibody contacts and epitope registries. Among the ten T-cell sub-tasks, HLA typing (T1) is anchored on IPD-IMGT/HLA with the 1000 Genomes and dbSNP reference panels. Antigen processing (T2) relies on IEDB and the Human MHC Ligand Atlas for natural cleavage sites, on NetChop-style cleavage training data and the C-terminal signal implicit in immunopeptidomes such as the SysteMHC Atlas, and, for the class-II conformational-stability arm, on protein structures drawn from the PDB and AlphaFold. MHC class I (T3) and class II (T4) binding are trained on IEDB together with the NetMHCpan and NetMHCIIpan binding-affinity and eluted-ligand corpora and the SysteMHC Atlas. Immunogenicity (T5) adds the assay-level annotations of IEDB, TANTIGEN, ITSNdb, dbPepNeo, and NEPdb, together with the cancer-epitope resource CEDAR and the integrated tumour-neoantigen database TumorAgDB2.0. TCR–peptide-MHC specificity (T6) is supported by the dedicated receptor databases VDJdb and McPAS-TCR together with the pan immune repertoire database PIRD and its TBAdb antigen-binding subset, by 10x Genomics single-cell and ImmuneCODE repertoire data, and by the IMMREP22 and Hi-TpH community benchmarks. Structural TCR modelling (T7) draws its templates and benchmarks from the PDB complexes together with the curated TCR-structure databases STCRDab and TCR3d and the ATLAS affinity-andstructure benchmark, with receptor sequences taken from VDJdb and the 10x Genomics repertoire data. Neoantigen identification (T8) consumes the clinico-genomic references TCGA, ICGC, COSMIC, and dbSNP alongside IEDB and the TESLA validation set. Tumor T-cell antigen classification (T9) is built from TANTIGEN, CAPD, and IEDB. Multi-epitope vaccine design (T10) combines IEDB and its population-coverage tool with the protective-antigen database Protegen, the VaxiJen and Vaxign servers, and pathogen proteomes retrieved from UniProt and GenBank. This task-level view shows that IEDB is the single connective resource spanning almost every sub-task, whereas most other databases are sharply task-specialised. The per-task databases are tabulated alongside their datasets in section 7.3 Benchmark datasets across the T-cell sub-tasks (T1–T10) and **Supplementary Table 3**.

Analysis of database support for standalone or multiple epitope prediction tasks (linear, conformational) reveals substantial overlap in usage across research domains. Fourteen database entries (29%) serve multiple epitope types. The Immune Epitope Database (IEDB) is the predominant cross-branch resource, and it contains 1.6 million epitopes that span linear B-cell, conformational B-cell, and T-cell epitopes across infectious, allergic, autoimmune, and transplant contexts. Other notable cross-branch databases include the HIV Molecular Immunology Database, CEDAR, AbYbank/AbDb, and FLAVIdB. This overlap suggests opportunities for unified dataset development and cross-task learning approaches. In addition to usage patterns, the database ecosystem is characterized by remarkable diversity in both scale and biological coverage. Data scale and scope diversity is evident in the range of database sizes, from highly specialized collections like EPITOME with 142 entries to comprehensive repositories such as IEDB, which contains 1.6 million epitopes. Species coverage spans multiple organisms, including both model organisms (human, mouse) and pathogen-specific collections (e.g., flaviviruses, HIV). Temporal coverage also varies significantly, with some databases offering historical data dating back to the 1990s, while others focus on contemporary high-throughput immunopeptidomics data generated by mass spectrometry platforms.

With the perspective of accessibility and maintenance status, these 48 database entries can be further segregated into three classes. From the 48 entries, 39 (81%) are actively maintained with regular updates and accessible interfaces, 4 (8%) are outdated with limited or discontinued updates, and 5 (10%) are inactive or inaccessible. This distribution highlights ongoing challenges in database sustainability, with approximately 19% of available resources suffering from maintenance issues that limit their utility for contemporary research. Beyond the fundamental classification, usage overlap, coverage, and accessibility patterns of these resources, it is important to consider how frequently and in what contexts different databases are actually utilized by the research community. Database usage pattern analysis reveals a pronounced distribution: 19 database entries (40%) are widely adopted across the field, while 14 entries (29%) remain significantly underutilized despite containing valuable data, and 15 entries (31%) serve specialized niche applications or are moderately used. IEDB dominates with universal adoption across the B-cell and T-cell prediction tasks, whereas comprehensive and actively maintained resources such as SysteMHC Atlas (2.1 million unique peptides) and CEDAR (under development in late alpha version) remain paradoxically underutilized. Furthermore, database heterogeneity extends to the use of distinct data formats across the ecosystem, including FASTA, XML, JSON, CSV, and specialized immunological formats. While FASTA and XML are prevalent in primary sequence repositories, structural databases predominantly use PDB and mmCIF (Macromolecular Crystallographic Information File) formats. This diversity of formats, coupled with inconsistent annotation standards and varying quality control procedures, presents significant barriers to large-scale data integration and standardized benchmarking. In a nutshell, this unified landscape analysis establishes the foundation for understanding how database selection and integration strategies impact the development of robust, generalizable epitope prediction models across all twelve prediction tasks.

## 7 Benchmark datasets: construction and availability

This section provides a comprehensive analysis of epitope prediction datasets by examining both the construction methodologies employed by researchers and the current availability landscape across linear B-cell, conformational B-cell, and T-cell epitope prediction tasks.

### 7.1 Benchmark datasets construction: single-database and multi-database integration strategies

To develop benchmark epitope prediction datasets using diverse databases, researchers are compiling positive and negative sequences using two primary strategies: single-database strategy and multi-database integration strategy. The single-database strategy utilizes individual databases as exclusive data sources to extract both positive and negative sequences. For example, for linear B-cell epitope datasets, researchers download complete B-cell assay data from comprehensive repositories like IEDB and filter for verified positive epitopes with length constraints of 5–25 amino acids. They create negative datasets by extracting non-epitope peptides that have showed no binding activity. single-database strategy yields datasets with good experimental validation but may lack broader biological context. Building on this limitation, pre-dominant strategy in literature is multi-database integration strategy which combines multiple databases to capture different types of biological information and improve dataset quality through cross-database validation. For example, linear B cell epitope datasets extract positive epitopes with different immunogenicity levels from Bcipep database within the same pathogen groups including virus, bacteria, protozoa, and fungi and negative or non-immunogenic sequences from IEDB database. Likewise, researchers extract positive linear B-cell epitopes directly from antibody-antigen complex structures provided by the abYbank/AbDb database by identifying antigen residues within 4A˚ radius of antibody atoms. Negative sequences are obtained from the Immune Epitope Database or generated by extracting separate peptide sequences from antigen regions that are distant from antibody binding sites within the same structural complexes.

For conformational B-cell epitope prediction datasets, researchers integrate data from multiple specialized databases including Protein Data Bank (PDB), Immune Epitope Database (IED), epitope registries, and structural antibody databases. Positive sequences are extracted directly from antigen-antibody complex structures sourced from the PDB where researchers identify amino acid residues that physically contact antibodies. The contact definition varies across studies with distance thresholds ranging from 4A˚ to 15A˚ between antigen and antibody atoms. Most approaches focus on surface-accessible residues by applying Relative Solvent Accessibility cutoffs. This ensures only exposed amino acids are considered as epitope candidates. Some methods supplement PDB-derived contact data with experimentally verified epitope information from the Immune Epitope Database or create epitope patches around antibody-verified binding sites obtained from specialized epitope registries like the HLA Eplet Registry. In contrast, negative sequences are selected using three primary strategies from the same antigen-antibody complex structures. The first strategy defines non-epitopes as surface residues that do not meet the distance criteria for antibody contact within the same complexes used for positive samples. The second approach generates synthetic negative examples by creating random permutations of true epitope sequences. These include complete sequence shuffling, partial amino acid rearrangement, or shifting epitope motifs to different protein regions.

The third strategy incorporates explicitly curated non-epitope datasets from databases like IEDB or selects surface residues that are not associated with known antibody recognition sites from epitope registries. This approach ensures that selected negative regions do not inadvertently contain actual epitope residues that could confuse model training. For T-cell epitope prediction datasets, researchers pre-dominantly combine positive tumor antigens from specialized databases like TANTIGEN with negative sequences from IEDB. Studies have used four different criterion namely rigorous filtering, disease-free association, MHC-binding without T-cell response, and unclear MHC context criterion to carefully select negative sequences. Specifically, the rigorous filtering criterion selects only antigens that have been experimentally tested and confirmed through *in vitro* and *in vivo* assays, restricts selection to MHC class I or II presented peptides from human sources with lengths between 9–14 amino acids, and discards any sequences associated with tumor, cancer, carcinoma, or metastasis terms. The disease-free association criterion selects negative samples that have no known association with any disease condition. The MHC-binding without T-cell response criterion targets antigens that bind to MHC molecules but show no documented evidence of eliciting T-cell responses. The unclear MHC context criterion involves negative samples where researchers did not clearly specify the MHC presentation context (class I or II), which represents the least effective approach for reliable model training.

### 7.2 Benchmark datasets availability

The availability of epitope prediction datasets varies significantly across the twelve prediction tasks summarised in **Figure 7**, with linear B-cell epitope datasets being the most abundant, followed by conformational B-cell epitope datasets, while the ten T-cell sub-tasks (T1–T10) each have comparatively few individually catalogued benchmark sets and instead rely on large shared training corpora and community benchmarks. This disparity reflects both the methodological complexity of different epitope types and the historical development trajectory of the field.

**Figure 7:**
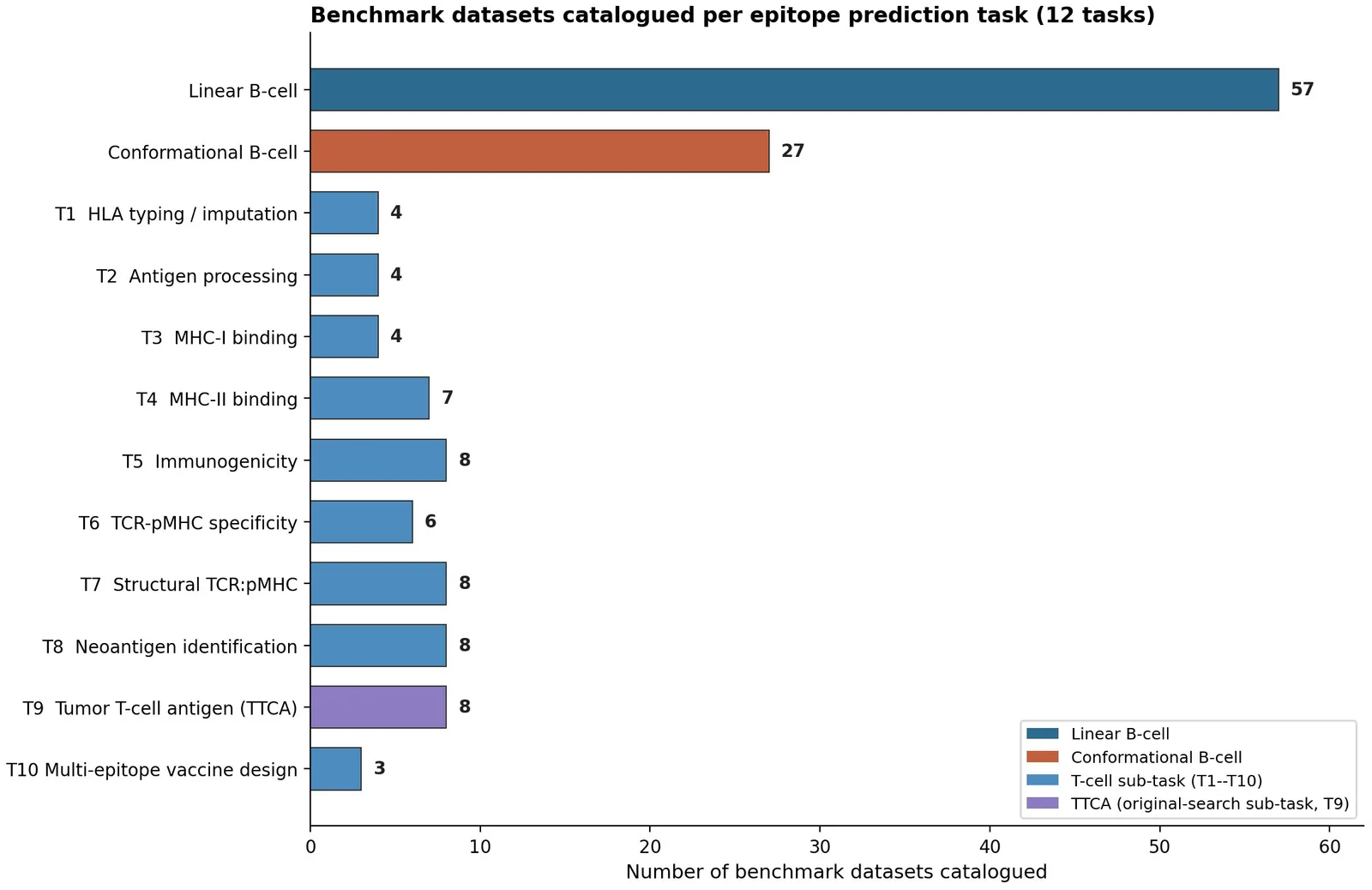
Number of benchmark datasets catalogued per epitope prediction task across all twelve tasks (linear B-cell, conformational B-cell, and the ten T-cell sub-tasks T1–T10). Linear and conformational B-cell datasets are individually enumerated (**Supplementary Table 1**; **Table 4**). The T-cell bars are the catalogued benchmark datasets per sub-task (**Supplementary Table 3**), and they sum with the linear and conformational sets to the 144 datasets reported in the text. The per-task totals are non-exhaustive, because large shared corpora contribute many derived training and evaluation splits that are counted once rather than individually.

For linear B-cell epitope prediction, 57 datasets have been developed with 49 being publicly available and 8 remaining private (**Supplementary Table 1**). Among public datasets, 45 are currently accessible while 4 have become inaccessible over time. The Lim et al. dataset (106) constitutes the largest publicly available resource with 301,915 labeled sequences, followed by IEDB38 (107) with 223,575 sequences. Among further large-scale resources, Yuan et al. (13) (128,959 sequences) is notable as the largest dataset with comprehensive immunoglobulin subtype annotations (IgA, IgE, IgM), followed by NetBCE (108) with 124,879 sequences and epitope1D Core (9) with 123,919 sequences. Seven datasets provide both core training sets and independent test sets, which enables robust model evaluation on non-overlapping data distributions. Species coverage demonstrates significant variation across datasets (**Supplementary Table 1**). The epitope1D dataset (9) exhibits high taxonomic diversity by incorporating Organism Ontology information as a feature, and it supports 20 taxonomy groups spanning Virus (Riboviria, Duplodnaviria, Monodnaviria, Varidnaviria), Bacteria (Terrabacteria group, Proteobacteria, PVC group, Spirochaetes, FCB group, Thermodesulfobacteria, Fusobacteria), and Eukaryota (Metamonada, Discoba, Sar, Viridiplantae, Opisthokonta) superkingdoms. The Lim et al. dataset (106) specifically addresses multi-species representation with comprehensive coverage across bacterial (40,949 peptides), fungal (1,346 peptides), viral (72,070 peptides), multicellular (3,495 peptides), and unicellular (184,055 peptides) organisms, which total 301,915 peptides. Eight specialized pathogenfocused datasets include individual collections for O. volvulus (109), Epstein-Barr virus (109), Hepatitis C virus (109), SARS-CoV epitopes (110), SARS-CoV-2 peptides (110), as well as private datasets for Zika virus and Dengue virus (111), and human-adapted viruses (112). These provide targeted resources for specific disease contexts.

Dataset composition approaches vary considerably (**Supplementary Table 1**). Some datasets emphasize balanced epitope-to-non-epitope ratios, such as ABCPred (10) (700:700), Chen (9, 10, 113) (872:872), and BCPreds (10) (701:701), while others maintain natural class distributions like NetBCE (108) (27,095:97,784). The LBtope collection provides multiple variants including LBtope (114) with 45,320 sequences, and specialized subsets like LBtope Confirm (115) and Lbtope Fixed non redundant (116). This offers flexibility for different modeling requirements. The Carmona et al. collection (117) provides 62,730 experimentally verified epitopes. Six datasets specifically focus on antibody class specificity, including the Tung et al. dataset (118) with 179,169 samples covering IgG (49,093), IgE (2,435), and IgA (631) classifications, and the Yuan et al. dataset (13) with detailed immunoglobulin subtype annotations (IgA=443, IgE=1,450, IgM=7,715). This diversity in dataset characteristics supports various research objectives from general epitope prediction to pathogen-specific vaccine development applications.

The landscape of conformational B-cell epitope prediction has been shaped by a diverse array of datasets that vary considerably in size, curation methodology, and public accessibility. A systematic analysis of thirteen representative studies from 2020 to 2024 reveals several noteworthy patterns and challenges inherent to this field, which are summarized in **Table 4** and **Figure 8**. The database sources from which conformational B-cell epitope prediction datasets are derived show consistent reliance on the Protein Data Bank (PDB) as the primary repository of structural information, with 11 of the 13 studies analyzed using PDB-derived antibody-antigen complex structures (3, 4, 6, 12, 14, 39, 119–123). Supplementary databases serve specialized roles in dataset construction. IEDB provides curated epitope annotations and serves as a source for both linear and conformational epitope data (2, 4). SAbDab offers antibody-specific structural information that has been used for independent test set construction (123). IMGT contributes immunogenetics data for antibody sequence analysis (122). SwissProt provides high-quality protein sequence data for antigen chain extraction (15, 39). The temporal growth of available structural data in PDB has enabled progressively larger training sets over time. This expansion is evidenced by the growth from 57 antigen chains in the original Ansari and Raghava 2010 benchmark used by Angaitkar et al. (4) to 1,645 antigen chains in SEMA 2.0 (15). The Cia et al. (119) benchmark combined PDB structures with the Antibody Database (AbDb) to construct a comprehensive evaluation resource containing 1,151 antibody-antigen complexes.

**Table 4:** Conformational B-Cell epitope prediction datasets.

Study	Database source	Epitope/non-epitope threshold	Dataset statistics	Experimental setting	Availability
Ivanisenko et al. (15)	SwissProt	Epi: ≤8Å Non-epi: 8–16Å Masked: >16Å	1) SEMA 2.0: 1,645 antigen chains Train: 1,544– Test: 101	90/10 Split	Public
Vardaxis et al. (122)	PDB, IMGT	Epi: ≤4Å from CDR Non-epi: No contact	1) Main: 1,003 antigens Epi: 6,986 | Non-epi: 209,580 2) SARS-CoV-2: 177 complexes Epitopes: 312	1) 5-fold CV 2) Independent	Private
Wang et al. (123)	PDB, SAbDab	Epi: <4.5Å Point cloud: <2Å Non-epi: ≥4.5Å	1) EpiPred: 148 complexes 2) BM: 44 complexes 3) SAbDab: 77 complexes 4) DiscoTope3: 24 complexes 5) SEDB: 89 complexes 6) Discotope: 56 complexes 7) Epitome: 78 complexes 8) SEMA: 103 complexes	Train/Test Split	Mixed (only 1st dataset is public)
Pandey et al. (39)	PDB	Epi: Antibody-interacting (Window = 17) Non-epi: Non-interacting	1) Cia Benchmark: 268 structures Pos: 5,049 | Neg: 5,049 (balanced)	5-fold CV	Public
Cia et al. (119)	PDB, AbDb	Epi: RSA≥10%, ΔRSA≥5% Non-epi: ΔRSA<5%	1) EAg: 1,151 complexes (Single epitope/antigen) 2) EAg_rep: 268 structures (70% sequence identity)	Benchmark	Public
Choi and Kim (6)	PDB	Epi: ≤4Å RSA≥15% (epitope3D) RSA≥10% (Benchmark) Non-epi: No contact	1) epitope3D: 245 antigens Epi: 6,018 | Non-epi: 110,702 2) Benchmark: 268 antigens 3) PUPre: 22 antigens Epi: 500 | Non-epi: 3,458	5-fold CV	Public
Singh et al. (14)	PDB	Epi: ≤4.5Å Non-epi: >4.5Å	1) Custom: 29 complexes Total residues: 29,929 Epi: 12,639 | Non-epi: 17,290	10-fold CV	Private
Angaitkar et al. (4)	IEDB, PDB	Epi: RSA>0 (visible) Non-epi: Surface exposed	1) Ansari & Raghava 2010: 57 chains Total residues: 10,547 Epi: 915 | Non-epi: 9,632	80/20 Split (SMOTE)	Public
Kumar et al. (2)	IEDB, BCEPS	Epi: Linear + Conformational (linearized) Non-epi: Negative samples	1) BCEPS: 7,871 peptides Epi: 3,875 | Non-epi: 3,996	5-fold CV	Public
da Silva et al. (3)	PDB	Epi: ≤4Å, RSA≥15% Non-epi: Balanced 1:2	1) epitope3D: 245 structures Total: 168,739 data points Epi: 6,018 | Non-epi: 110,702	10-fold CV	Public
Shashkova et al. (120)	PDB	Epi: ≤8Å Non-epi: 8–16Å Distant: >16Å	1) SEMA: 884 sequences Train: 783 | Test: 101	90/10 Split	Public
Lu et al. (12)	PDB	Epi: ≤4.5Å, RSA≥0.01 Non-epi: >4.5Å	1) EpiPred: 103 complexes 2) DBD v5: 59 complexes Combined: 162 complexes Epi: 4,305 | Non-epi: 31,554	LOOCV	Public
Solihah et al. (121)	PDB	Epi: ≤4Å (VdW), RSA≥0.01 Non-epi: Non-binding	1) Rubinstein’s: 76 chains (62 cplx) 2) Kringelum: 39 chains 3) SEPPA 3.0: 90 chains	LOOCV	Public (1st dataset not accessible)

Epi, Epitope; Non-epi, Non-Epitope; CV, cross-validation; LOOCV, leave-one-out CV; RSA, relative solvent accessibility; ΔRSA, change in RSA upon binding; VdW, van der Waals; CDR, complementaritydetermining region; SMOTE, Synthetic Minority Over-sampling Technique.

**Figure 8:**
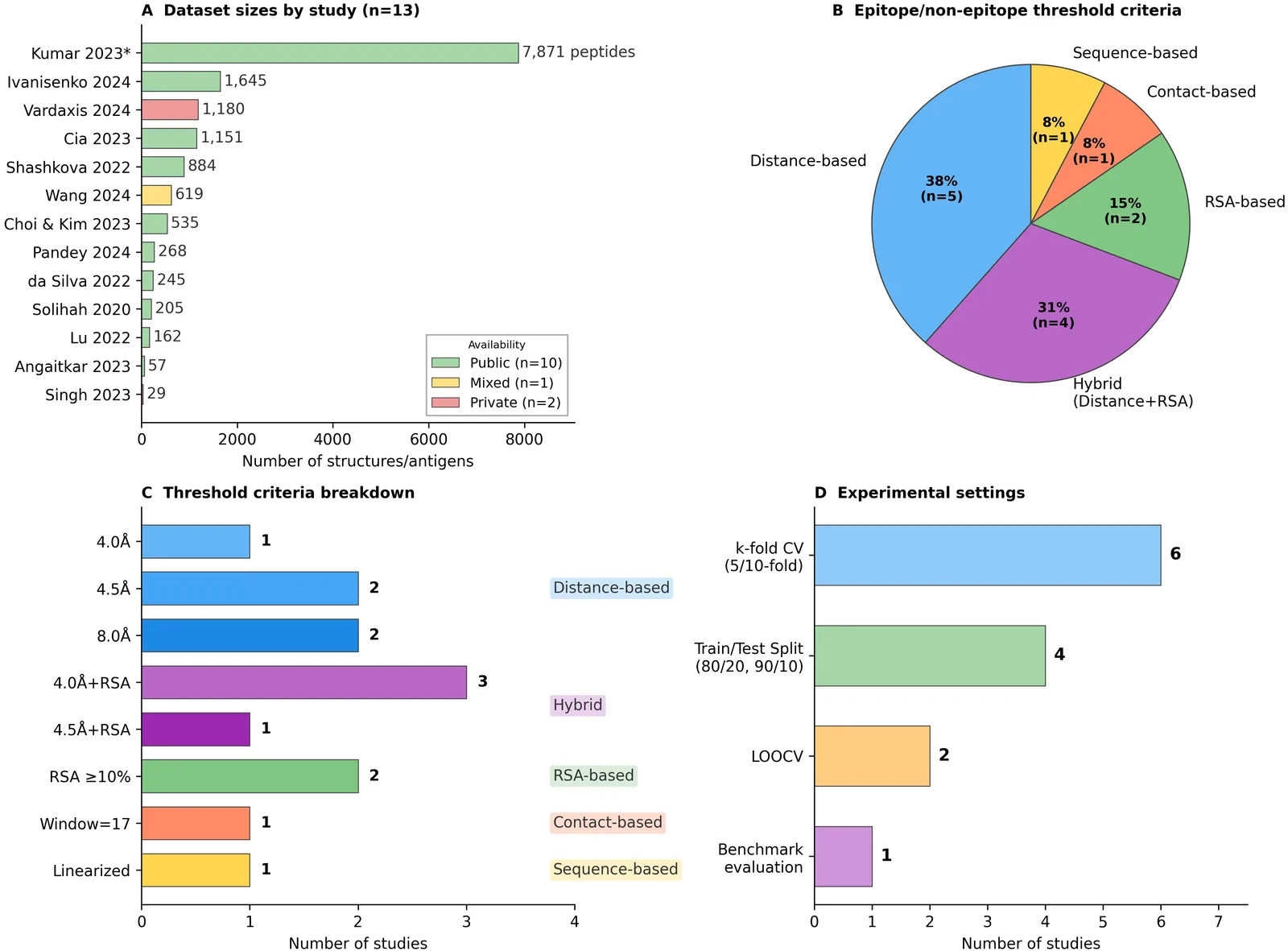
A comparison of conformational epitope prediction datasets: **(A)** In terms of size and public availability, **(B)** five different threshold criterion used to define epitopes and non-epitopes, **(C)** variations in threshold value, **(D)** experimental settings used to perform experiments.

Dataset sizes across the thirteen studies span a remarkable range from 29 to 7,871 samples, as illustrated in **Figure 8A**. The majority of datasets contain fewer than 1,000 structures or antigens. Kumar et al. (2) assembled the largest dataset with 7,871 peptides comprising 3,875 B-cell epitopes and 3,996 non-B-cell epitopes. This dataset combines both linear and conformational epitopes in a linearized format derived from BCEPS, ILED, and IDED databases. In contrast, Singh et al. (14) used the smallest dataset containing only 29 complexes with 29,929 total amino acids. Of these residues, 12,639 were labeled as epitopes and 17,290 as non-epitopes. The SEMA 2.0 dataset contains 1,645 antigen chains from SwissProt with 1,544 chains allocated for training and 101 for testing (15). This represents a substantial expansion from the original SEMA dataset of 884 antigen sequences clustered at 95% identity (120). The Cia et al. (119) benchmark provides two dataset variants: EAg with 1,151 complexes containing single epitopes per antigen, and Erep Ag with 268 representative structures filtered at 70% sequence identity. Vardaxis et al. (122) constructed a training database of 1,003 antigen sequences containing 6,986 unique conformational B-cell epitopes. Their unbound structure collection for 3D macrostructure models included 41,592 structures. The epitope3D dataset comprises 245 non-redundant unbound antigen structures with 168,739 data points (3). Only 3.56% of these data points represent epitope residues, while 53.82% are surface residues. Wang et al. (123) utilized multiple datasets for comprehensive evaluation. Their training set contained 118 complexes from EpiPred, validation used 44 complexes from the BM dataset, and testing employed 30 EpiPred complexes plus 77 SAbDab complexes.

The criteria used to define epitope and non-epitope residues exhibit considerable heterogeneity across studies, as demonstrated in **Figure 8B**. Distance-based thresholds dominate the field at 38% of studies (n=5). Hybrid approaches combining distance and relative solvent accessibility account for 31% (n=4). Pure RSA-based definitions represent 15% (n=2). Contact-based and sequence-based methods each constitute 8% (n=1). The SEMA framework introduced a sophisticated three-zone labeling scheme (15, 120). Residues within distance R1 of the antibody are classified as epitopes. Residues between R1 and R2 are labeled as non-epitopes. Residues beyond R2 are masked during training to avoid ambiguous labels. This masking strategy addresses the fundamental uncertainty that non-epitope residues might belong to yet-undiscovered epitopes. The validity of this concern is demonstrated by extensively studied antigens such as lysozyme, where epitopes cover almost the entire surface with 70 out of 85 surface residues belonging to at least one known epitope (119). The Cia et al. (119) benchmark employed a distinct definition based on changes in solvent accessibility upon antibody binding. Epitope residues were defined as surface residues with RSA ≥ 10% and ΔRSA ≥ 5%, where ΔRSA represents the difference between unbound and bound RSA values. Non-epitope residues showed ΔRSA ≪ 5%. Vardaxis et al. (122) defined epitopes as amino acids with atoms within contact distance of antibody CDR regions. Non-epitope residues were represented as zeros in their positional permutation vector encoding scheme.

**Figure 8C** provides a granular breakdown of specific threshold values employed across studies. The most common hybrid approach uses a 4.0 A˚ distance cutoff combined with RSA constraints (n=3). Pure distance thresholds of 4.5 A˚ and 8.0 A˚ are each used by two studies. The 4.0 A˚ threshold typically reflects direct van der Waals contacts between antigen and antibody atoms. Larger radii such as 8.0 A˚ additionally capture residues involved in long-range interactions (120). The SEMA models were trained with R1 = 8.0 A˚ and R2 = 16.0 A˚ after systematic evaluation of multiple radius combinations (15, 120). The epitope3D method employs a 4 A˚ distance cutoff combined with an RSA threshold of 15% (3). Unbound antigen structures were required to have 100% structural alignment and at least 70% sequence similarity with corresponding bound complexes. Vardaxis et al. (122) used a 4 A˚ threshold from antibody CDR regions. Wang et al. (123) employed a 4.5 A˚ distance for epitope definition and a stricter 2 A˚ threshold for point cloud binding surface annotation. The choice of RSA threshold significantly affects class imbalance ratios. Solihah et al. (121) observed that larger surface exposure thresholds reduce predictive performance. Their choice of 0.01 as the RSA limit aligns with the finding that all epitope RSA values are positive, even when only slightly greater than zero. Angaitkar et al. (4) considered all visible residues with *RSA>>>* 0 as potential epitope candidates.

The severe class imbalance between epitope and non-epitope residues represents one of the most persistent challenges in this field. The epitope3D dataset exemplifies this problem with an imbalance ratio of approximately 1:29 between epitope and non-epitope classes (3). Various strategies have been employed to address this issue. Solihah et al. (121) introduced CluSMOTE, which combines cluster-based undersampling using hierarchical DBSCAN with Synthetic Minority Oversampling Technique. The epitope3D methodology applies a two-stage balancing approach (3). First, the majority class is randomly undersampled until the imbalance reduces from 1:29 to 1:8. Then, SMOTE oversampling of the minority class achieves a final 1:2 distribution containing 50,036 residues with 33.33% epitopes. Angaitkar et al. (4) employed SMOTE on a dataset where the training set originally contained 750 epitopes and 7,687 non-epitopes. After SMOTE application, both classes contained 7,687 samples each. Pandey et al. (39) created balanced datasets with equal numbers of positive and negative patterns for both training (3,980 each) and validation (1,069 each). The EpiCluster training set contained 5,106 epitopes and 98,145 non-epitopes at an imbalance ratio of 1:19.2 (6). The experimental settings and validation strategies employed across studies show notable variation, as depicted in **Figure 8D**. K-fold cross-validation is the most prevalent approach, used by six studies. Train/test splits at various ratios are employed by four studies. Leave-one-out cross-validation is used by two studies. Formal benchmark evaluation without model training is conducted by one study. Pandey et al. (39) employed 5-fold cross-validation on 214 antigens for model development while reserving 54 antigens as an independent validation set. They systematically tested window sizes ranging from 7 to 31 residues, with 17 residues identified as optimal.

Lu et al. (12) used complex-based LOOCV on their combined EpiPred and DBD v5 dataset. Their training set contained 103 complexes with 2,708 epitopes and 19,567 non-epitopes. Vardaxis et al. (122) employed a temporal split strategy. They allocated 94% of older PDB structures for training and reserved 6% of the most recent structures for testing. Cross-validation models were trained for 459 epochs, while independent test models were trained for 583 epochs. The SEMA framework implemented a retrospective test design (120). The test set included only structures released in PDB after January 1, 2020, with less than 70% sequence identity to training sequences. Choi and Kim (6) applied different sequence similarity cutoffs depending on the evaluation context: 70% for the epitope3D dataset and 99% for the 2023 benchmark set. Wang et al. (123) maintained strict separation between data splits, with antigen sequence similarity below 90% between training and validation sets and below 25% between the BM validation set and EpiPred test set. The availability of datasets for reproducible research represents a critical concern that warrants careful examination. Across the thirteen studies analyzed, a total of 27 distinct datasets are referenced in **Table 4**. Of these, 17 are publicly available whereas 10 remain private. The distribution of private datasets is concentrated in three studies. Wang et al. (123) evaluated their WUREN method across eight benchmark datasets: EpiPred, BM, SAbDab, DiscoTope3, SEDB, Discotope, Epitome, and SEMA. Only the EpiPred dataset is publicly accessible, whereas the remaining seven are private. This substantially limits reproducibility of their comprehensive multi-dataset evaluation. Vardaxis et al. (122) utilized two private datasets. Their main training database contains 1,003 antigen sequences with 6,986 unique conformational B-cell epitopes. Their SARS-CoV-2 validation set comprises 177 spike protein-antibody complexes with 312 epitopes. The dataset from Singh et al. (14) containing 29 antigen-antibody complexes also remains private. The Solihah et al. (121) dataset presents a partially accessible situation. The two test sets (Kringelum with 39 antigen chains and SEPPA 3.0 with 90 antigen chains) are accessible. However, the original Rubinstein training dataset of 76 antigen chains from 62 complexes is no longer available. Public availability has enabled standardized benchmarking across the field. The Cia et al. (119) repository at https://github.com/3BioCompBio/BCellEpitope provides both the EAg dataset of 1,151 structures and the representative Erep Ag dataset of 268 structures. These resources have been adopted by subsequent methods including CBTOPE2 (39) and EpiCluster (6). The epitope3D dataset is freely available through a web interface at http://biosig.unimelb.edu.au/epitope3d with an API for pipeline integration (3). The SEMA codebase and trained models are available at https://github.com/AIRI-Institute/SEMAi with web interfaces at https://sema.airi.net (15, 120). The careful curation of sequence identity thresholds varies substantially across studies. Values range from 25% between training and test sets in Wang et al. (123) to 99% in the EpiCluster 2023 benchmark evaluation (6). This variation complicates direct comparison of reported results across different studies and highlights the need for standardized dataset construction protocols in future work.

### 7.3 Benchmark datasets across the T-cell sub-tasks (T1–T10)

The ten T-cell prediction sub-tasks (T1–T10) are supported by substantially larger and more diverse resources than the linear and conformational B-cell sets catalogued above, and these are summarised in **Table 5**. These resources divide into dedicated immunology databases (IEDB, VDJdb, McPAS-TCR, TANTIGEN, IPD-IMGT/HLA, and SysteMHC Atlas), large training corpora (the NetMHCpan and NetMHCIIpan binding-affinity and eluted-ligand (BA/EL) data, 10x Genomics single-cell data, and ImmuneCODE), structural repositories (PDB and STCRDab), and clinico-genomic references (TCGA, ICGC, COSMIC, 1000 Genomes, and dbSNP). Community benchmarks such as IMMREP22 (124) for TCR specificity and Hi-TpH (125) for TCR-pHLA binding, together with the benchmark and review literature (18, 19, 32, 126, 127), provide an evaluation infrastructure that is far richer than the three core TTCA datasets which anchor most TTCA publications. Full per-sub-task dataset statistics, including approximate scale, MHC class, and accessibility, are tabulated in **Supplementary Table 3**, and representative per-study methods with their representation learning, classifiers, headline performance, and code availability are listed in **Supplementary Table 4**. The compact summaries in **Figures 9** and section 10.3 visualise the dataset scale and best-reported performance ranges across sub-tasks. Fragmented evaluation and weak generalisation to unseen epitopes nonetheless remain field-wide concerns. Among these sub-tasks, the tumor T-cell antigen (TTCA, T9) datasets are the most fully characterised, and they are examined in detail in the following subsection.

**Table 5:** Key data resources per T-cell sub-task (T1–T10) and their representative predictors.

Resource	Sub-task	Size/scope	Type	Representative usage
IEDB	T2–T6, T8–T10	>600,000 epitope/assay records	Immunology DB	NetMHCpan, MHCflurry, PRIME, Repitope (49, 50, 63, 65)
NetMHCpan/ NetMHCIIpan BA/EL	T3/T4	∼105–106 ligand entries	Training corpus	NetMHCpan-4.1, NetMHCIIpan-4.0, NNAlign MA (49, 58)
SysteMHC Atlas	T3/T4	Immunopeptidomics spectra	Immunology DB	TransBindpMHCI (128), MHCflurry (50)
NetChop/ NetCTLpan data	T2	∼106 MS cleavage sites; 504 NetCTLpan pairs	Training corpus	NetCTLpan, PU-cleavage, Ziegler (45, 47, 129)
ITSNdb/immunogenicity sets	T5	∼104–105 immunogenicitylabelled peptides (e.g. DeepHLApan 327,178; Repitope 52,855; NeoTImmuML 10,312)	Immunog. benchmark	PRIME, DeepImmuno, ImmunoStruct (63, 64, 66)
VDJdb	T6	>90,000 TCR–epitope pairs	TCR DB	NetTCR, ERGO-II, TITAN, epiTCR, STAPLER (68, 70–72, 78)
McPAS-TCR	T6	∼5,000+ entries	TCR DB	pMTnet, DLpTCR (130, 131)
10x/ImmuneCODE	T6	paired αβ/SARSCoV-2 repertoires	Singlecell/repertoire	DeepTCR, MixTCRpred (75, 132)
IMMREP22/ Hi-TpH	T6	community benchmarks	Benchmark	generalisation evaluation (124, 125)
PDB, ATLAS, TCR3d, STCRDab	T7	∼hundreds of solved complexes; ∼16,500 modelled poses	Structure DB/benchmark	TCRdock, TCRmodel2, NetTCRstruc, TCRcost (20, 80–82)
IPD-IMGT/HLA	T1	thousands of alleles	Reference DB	HLA-HD, CookHLA, DEEP-HLA (40, 41, 43)
TCGA/COSMIC/dbSNP	T8	somatic/germline variants	Genomic ref.	pVACtools, pTuneos (85, 133)
TANTIGEN/CAPD	T9	tumor antigens	Immunology DB	iTTCA family, ENCAP

The intrinsic properties, scale, and maintenance status of the dedicated databases listed here are catalogued in **Table 3**. This table instead maps each resource to the sub-tasks it serves and to the representative predictors that consume it.

**Figure 9:**
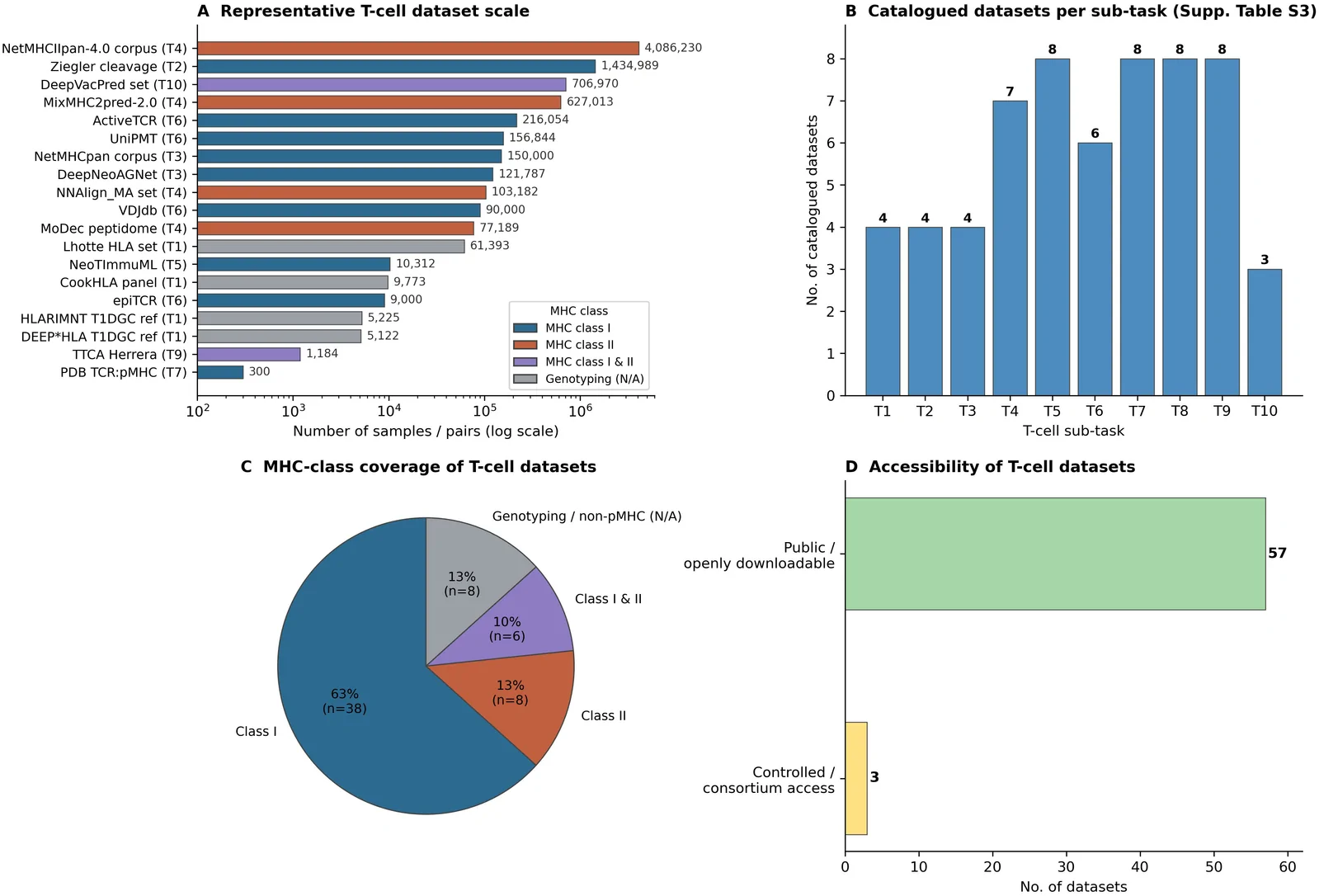
Dataset landscape across the ten T-cell prediction sub-tasks (T1–T10). **(A)** Approximate scale of representative datasets and corpora on a logarithmic axis, coloured by predominant MHC class. **(B)** Number of catalogued benchmark datasets per sub-task (**Supplementary Table 3**). **(C)** MHC-class coverage across the catalogued datasets. **(D)** Public versus controlled-access distribution. Sizes are approximate and as reported by the source studies.

### 7.4 Tumor T-cell antigen (T9): benchmark datasets

We close the T-cell dataset survey with the tumor T-cell antigen (TTCA, T9) sub-task, whose public benchmark sets are the most fully characterised of the T-cell landscape. The corresponding TTCA performance analysis is presented in the performance section below. For the tumor T-cell antigen (TTCA) classification sub-task, eight datasets have been developed from 2019 to 2026 using IEDB, TANTIGEN, and CAPD databases, with seven datasets being publicly available and one remaining private. Among the seven public datasets, five serve as benchmark datasets while two function as small independent test sets containing only experimentally verified tumor T-cell antigen sequences.

The single private dataset is the earliest one, introduced by Lissabet et al. for TTAgP 1.0 (134), which released neither its sequences nor a usable training resource and is therefore shown as private in **Figure 10**. Detailed statistics of the five benchmark datasets across train and test sets as well as 2 independent test sets are shown in **Figure 10**. Two of the five benchmark sets are not new independent collections but re-derivations of the earlier three. The LYnet set (99) was built by merging the train and test partitions of the iTTCA-Hybrid, TAP 1.0, and iTTCA-RF datasets and removing duplicate sequences, which yields the largest resource with 2,169 sequences comprising 1,184 positive and 985 negative peptides. The PepBenchmark set (135) reuses the TAP 1.0 positive peptides but replaces the negatives with a freshly generated, distribution-matched set. The result is 1,064 sequences split evenly into 532 positive and 532 negative peptides. Among the original collections, the Herrera et al. dataset (136) is the largest with 1,184 sequences comprising 592 positive and 592 negative sequences, followed by the Charoenkwan et al. dataset (137) with 985 sequences containing 592 positive and 393 negative sequences, and the Sotirov et al. dataset (138) with 424 sequences comprising 212 positive and 212 negative sequences.

**Figure 10:**
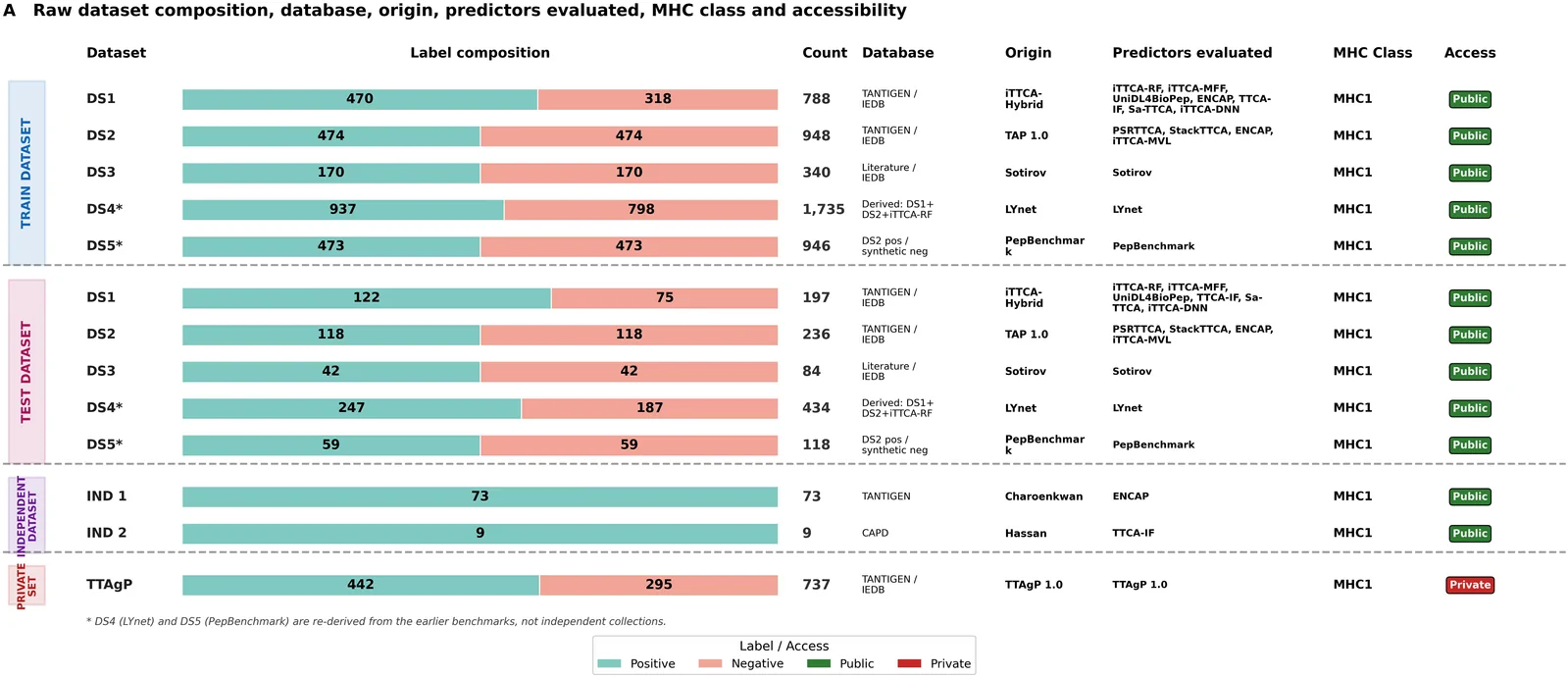
Statistics of the seven publicly available tumor T-cell Antigen (TTCA) Classification datasets together with the private TTAgP 1.0 set, along with their inherent databases, accessibility, and utility across studies. The LYnet and PepBenchmark sets are re-derived from the earlier benchmarks rather than newly collected. (Datasets for the broader T-cell sub-tasks are summarised in **Table 5**).

A closer look at how these datasets are assembled shows that the positive class is constructed consistently across the field, whereas the negative class is not, and this asymmetry is a central comparability problem for the sub-task. Nearly every study draws its positive MHC class I tumor antigens from the same source, namely the TANTIGEN and TANTIGEN 2.0 repositories, as in iTTCA-Hybrid (137), TAP 1.0 (136), TTAgP 1.0 (134), iTTCA-RF (92), ENCAP (97), TTCA-IF (139), and Sa-TTCA (140). The single exception is Sotirov et al. (138), who curated 212 experimentally validated immunogenic human tumor peptides from the literature and IEDB under the stricter requirement of both a positive MHC-binding assay and a positive in-human T-cell assay. The negative class, by contrast, is defined in four distinct ways. The dominant approach treats T-cell antigens from IEDB that carry no disease association as presumed non-antigens, which is the strategy of iTTCA-Hybrid, TAP 1.0 (after keyword filtering that excludes cancer, carcinoma, and metastasis terms), TTAgP 1.0, iTTCA-RF, ENCAP, TTCA-IF, and Sa-TTCA. The second approach, used by Sotirov et al., builds a true negative set of peptides that bind MHC but are experimentally non-immunogenic. The third approach, introduced by PepBenchmark (135), generates synthetic negatives whose amino-acid composition and length distributions are matched to the positives so that the two classes are not separable by shallow features. The fourth case, LYnet (99), simply inherits the negatives of the three datasets it merges. PSRTTCA (95) explicitly flagged that the negatives of earlier studies were of unclear provenance and possibly incorrect, which motivated its propensity-score reweighting. The practical consequence is that headline accuracies are not directly comparable across studies, because a peptide labelled negative in one benchmark may be a confirmed non-immunogen, a presumed non-antigen, or a distribution-matched decoy, and the choice of negative set is itself a confounding variable.

Dataset utilization patterns reveal significant variation in adoption rates across the research community. The Charoenkwan et al. dataset (137) demonstrates the highest usage frequency and appears in 6 studies (92–97), while the Herrera et al. dataset (136) appears in 5 studies (94– 97, 139). The Sotirov et al. benchmark dataset (138) shows limited adoption with usage in only 1 study. Two small independent test sets supplement the benchmark resources, including the Charoenkwan positive dataset (137) containing 71 tumor T-cell antigen sequences and the Hassan et al. positive dataset (139) with 9 tumor T-cell antigen sequences, each utilized by 1 study. Studies have consistently employed 80% sequences for training and 20% for testing across all benchmark datasets, supplemented with 10-fold cross-validation. Hassan et al. (139) conducted evaluation on both the Charoenkwan et al. benchmark dataset and their independent test set of 9 sequences, while Yu et al. (97) performed the most comprehensive evaluation across 2 benchmark datasets and 1 independent test set. Despite the availability of seven public datasets, no study has performed evaluation across all available resources, and this indicates fragmented evaluation practices in tumor T-cell antigen prediction research. The Charoenkwan et al. benchmark remains the most widely reused resource, and beyond the studies above it also served as the evaluation set for the deep neural network iTTCA-DNN (98) and for the protein language model framework UniDL4BioPep (141), while LYnet (99) and PepBenchmark (135) evaluated on the re-derived sets they introduced rather than on the original benchmarks.

## 8 Architectural components of AI-driven predictive pipelines

A comprehensive analysis of 155 studies indicates that across linear B-cell, conformational Bcell, and T-cell epitope prediction tasks, AI-driven approaches largely conform to a two-stage architectural framework consisting of representation learning based on sequence or structureaware encodings, followed by classification algorithms. This section systematically categorizes these components and examines their methodological commonalities across epitope prediction paradigms.

### 8.1 Sequence representation learning approaches

The computational identification of B- and T-cell epitopes relies on transforming biological sequences into numerical representations that capture patterns underlying immune recognition. Our systematic analysis of the current literature reveals 272 unique encoding methods distributed across linear B-cell epitope prediction with 65 approaches, conformational B-cell epitope prediction with 40 approaches, and T-cell epitope prediction with 167 approaches that span the full set of ten T-cell sub-tasks (T1–T10) and cover MHC class I and class II binding, antigen processing, immunogenicity, TCR–peptide specificity, neoantigen, structural, HLA-typing, and vaccine-design encodings (see Section 5), organized into 13 distinct categories (**Figure 11**).

**Figure 11:**
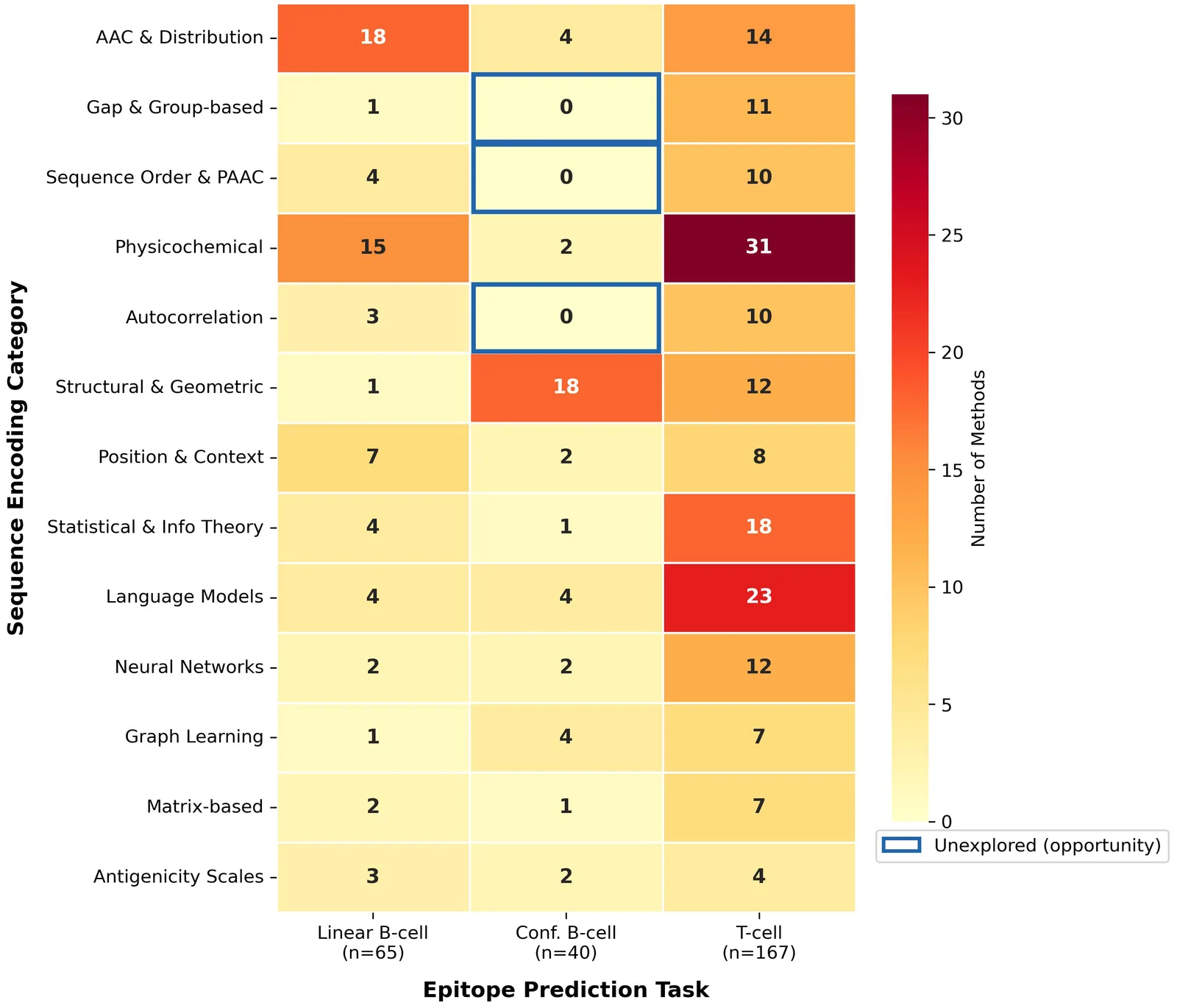
Distribution of representation learning approaches across 13 encoding categories and the linear B-cell, conformational B-cell, and T-cell prediction tasks (*n* = 272 total; Linear B-cell *n* = 65, Conf. B-cell *n* = 40, T-cell *n* = 167 across the full ten-sub-task T-cell landscape T1–T10). Color intensity indicates utilization frequency. Blue-bordered cells denote categories with zero usage in the respective task. Across the T-cell sub-tasks, language models (*n* = 23), graph learning (*n* = 7) and position/context encodings (*n* = 8) are all well represented. The task-specific patterns are that structural features dominate conformational B-cell prediction (45%), physicochemical properties lead T-cell prediction (approximately 19%), and composition-based methods lead linear B-cell prediction (28%).

Foundational sequence encoders based on amino acid composition and distribution, including amino acid composition, dipeptide composition, and k-mer variants, capture residue frequency patterns within epitope sequences and are extensively used in linear B-cell prediction, where 18 methods exploit their effectiveness in identifying amino acid preferences characteristic of immunogenic regions. Gap and group-based encoders such as the composition of k-spaced amino acid pairs introduce spatial relationships between residues and prove particularly valuable for T-cell prediction, where eleven approaches reflect the importance of amino acid spacing in MHC binding. Sequence order and pseudo-composition descriptors, including pseudo amino acid composition, amphiphilic pseudo amino acid composition, and the CTD composition–transition–distribution family, further capture sequential organization patterns that are critical for binding specificity. Physicochemical property encoders encompassing hydrophobicity indices, electrostatic charge, molecular weight, and flexibility constitute the most extensively utilized category overall and dominate Tcell epitope prediction, and they account for 31 approaches, or roughly 19 percent, of all T-cell methods. This dominance underscores the central role of amino acid chemical characteristics in peptide–MHC interactions and T-cell receptor recognition. Autocorrelation and covariance methods such as Moran and Geary autocorrelation functions extend this perspective by capturing spatial correlation patterns in physicochemical properties and appear in ten T-cell approaches. Within the neoantigen-identification sub-task (T8), this physicochemical and engineered-feature family takes a distinctive form: rather than encoding the peptide sequence alone, T8 pipelines combine predicted binding affinity with neoantigen-specific descriptors such as the differential agretopicity index (DAI), variant allele frequency, clonality, expression level, and foreignness or similarity-to-self, which quantify how a mutant peptide differs from its wild-type and self counterparts (85, 86, 142). Two T8 studies depart from this hand-crafted template. NeoGuider transforms each raw feature into an immunogenicity log-odds value through adaptive kernel-density estimation followed by isotonic and centered-isotonic regression before a logistic-regression readout (86), and CNNeoPP encodes peptide and HLA sequences with natural-language-processing representations, namely term-frequency-inverse-document-frequency vectors and fine-tuned BioBERT embeddings, and fuses these with one-hot categorical features (88). In contrast, conformational B-cell epitope prediction is strongly centered on structural and geometric features, with 18 of 40 approaches relying on descriptors such as relative solvent accessibility, accessible surface area, half-sphere exposure, and protrusion indices that are essential for modeling three-dimensional antigenic surfaces. Position- and context-based methods, including binary profile patterns and position-specific information, are preferentially employed in linear B-cell prediction, where seven approaches emphasize the influence of precise amino acid positioning on antibody recognition. Statistical and informationtheoretic approaches, including term frequency–inverse document frequency, Shannon entropy, and variational inference methods, are extensively adopted in T-cell prediction, with 18 approaches aimed at capturing complex sequence patterns and quantifying positional conservation.

More recent advances are represented by language models and transformer-based encoders such as ESM, ProtBERT, and EpiBERTope, which are applied extensively across both B-cell prediction (eight approaches) and the T-cell sub-tasks, where the *n* = 23 language-model encodings now span MHC binding, TCR specificity, and antigen processing, and these protein-languagemodel approaches are analysed together with their representative tools in Section 9. These models learn rich contextual representations from large protein databases, and they therefore capture longrange dependencies that are inaccessible to traditional encoders. Graph and geometric learning methods, including graph convolutional and heterogeneous-graph networks, appear in conformational B-cell prediction (four approaches) and, increasingly, in T-cell prediction (*n* = 7), where HeteroTCR (143), UniPMT (76), ImmunoStruct (66), and NetTCRstruc (81) model TCR–pMHC connectivity and structure. Neural network architectures such as LSTM variants, ProtVec embeddings, and attention mechanisms exhibit balanced usage across all tasks, while matrix-based substitution approaches leverage evolutionary information through position-specific scoring matrices and BLOSUM matrices, and antigenicity scale-based methods employ empirically derived propensity scores to quantify antigenic potential. Collectively, **Figure 11** highlights methodological patterns across the B-cell and T-cell branches. Across the ten T-cell sub-tasks, language models (*n* = 23), graph learning (*n* = 7), and position/context encodings (*n* = 8) are all well represented in T-cell prediction. The genuine gaps are task-specific. Autocorrelation and statistical encodings are absent from conformational B-cell prediction, and graph and structure-aware encodings remain comparatively under-used in linear B-cell prediction. These residual gaps represent concrete opportunities for cross-task knowledge transfer. These gaps, together with the pronounced task-specific preferences observed, such as physicochemical dominance in T-cell prediction and structural feature concentration in conformational B-cell prediction, point to substantial opportunities for improving epitope prediction performance through the strategic transfer and adaptation of successful representation learning approaches across tasks.

### 8.2 Classification approaches

The computational identification of B and T cell epitopes has employed diverse machine learning paradigms that range from traditional statistical models to advanced deep learning architectures. Our comprehensive analysis of 148 predictive models reveals a sophisticated ecosystem of classification approaches distributed across 32 linear B-cell, 13 conformational B-cell, and 103 T-cell epitope prediction studies (the latter spanning the ten sub-tasks T1–T10). These classifiers can be systematically organized into five categories as illustrated in **Figure 12**, and each category represents different computational philosophies for pattern recognition in immune target prediction.

**Figure 12:**
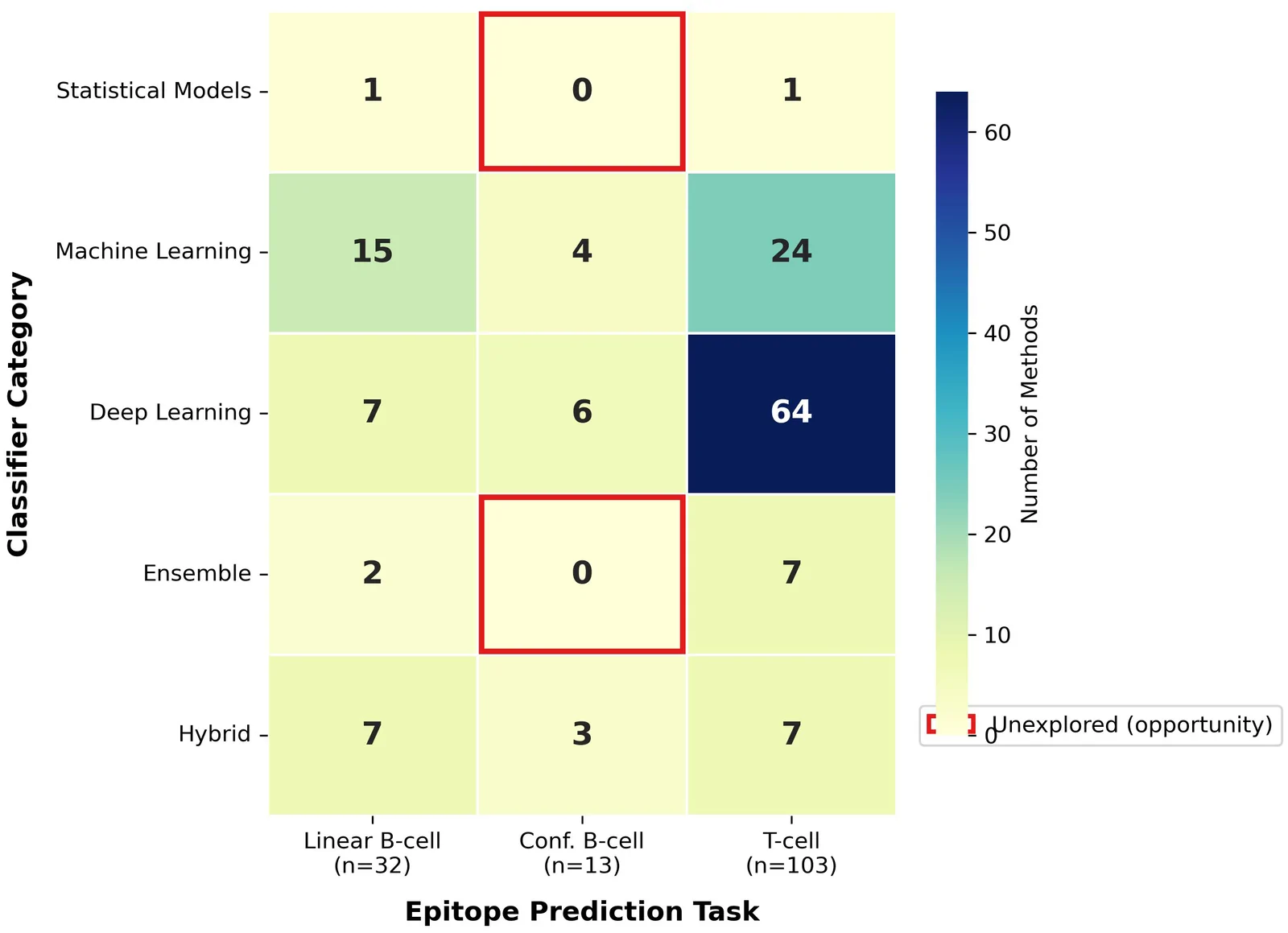
Distribution of classification approaches across five classifier categories and the linear B-cell, conformational B-cell, and T-cell prediction tasks, with the T-cell column spanning the full ten-sub-task landscape (T1–T10, *n* = 103). Deep learning (blue) is the dominant paradigm in T-cell prediction (64 of 103, 62%), and this dominance is driven by the CNN, LSTM, attention, transformer, graph, and protein-languagemodel architectures used in MHC binding, immunogenicity, and TCR specificity. Deep learning also leads conformational B-cell prediction (46%), while machine learning (green) dominates linear B-cell prediction (47%). Ensemble and statistical methods remain absent in conformational B-cell prediction, and this absence represents an open opportunity for cross-task adaptation.

Statistical Models represent the foundational computational approaches in epitope prediction and employ probabilistic principles to distinguish epitope regions from non-epitope sequences. These methods maintain a minimal but persistent presence, with one approach in linear B-cell prediction and two across the ten T-cell sub-tasks (T1–T10), while remaining absent in conformational B-cell prediction. Quadratic Discriminant Analysis (QDA) serves as the primary statistical classifier and estimates class-conditional probability distributions to determine optimal decision boundaries through analysis of the covariance structure of different epitope classes. Genetic Algorithm represents an evolutionary computation approach that mimics natural selection to optimize classifier parameters through iterative evolution of solution populations via selection, crossover, and mutation operations. The limited adoption of purely statistical approaches reflects the increasing complexity of epitope prediction problems that demand more sophisticated pattern recognition capabilities, although their interpretability and theoretical grounding continue to provide value in specific applications.

Traditional Machine Learning algorithms constitute a major category of classifiers with 15 of 32 approaches in linear B-cell prediction, 4 of 13 in conformational B-cell prediction, and 24 of 103 across the ten T-cell sub-tasks, where they remain especially prominent in tumor antigen classification (T9), immunogenicity (T5), and efficient TCR predictors such as epiTCR and TCR-H (T6). Tree-based methods form a significant subset that includes Decision Trees, Random Forest (RF), Extra Trees (ET), and gradient boosting variants such as XGBoost, LightGBM, Gradient Boosting Classifier (GBC), and CatBoost. Random Forest has gained widespread adoption in T-cell epitope prediction due to its ability to handle high-dimensional feature spaces while providing inherent feature importance rankings, and its ensemble nature makes it robust to overfitting while capturing complex non-linear relationships between amino acid properties and epitope activity. Recent conformational B-cell epitope predictors including CLBTope (2) and CBTOPE2 (39) have employed comprehensive machine learning pipelines that integrate multiple algorithms such as RF, Gradient Boosting, Naive Bayes, Logistic Regression, LGBM, and KNN to optimize prediction performance. Support Vector Machines (SVM) appear across the B-cell and T-cell prediction tasks and excel at finding optimal decision boundaries in high-dimensional spaces through the kernel trick, which enables capture of non-linear relationships without explicit computation of complex feature transformations. Least Squares SVM (LSSVM) provides a computationally efficient alternative to traditional SVM formulations and has been successfully applied to T-cell epitope prediction. Machine learning accounts for essentially all classifiers in the tumor T-cell antigen sub-task (T9), but across the full ten-sub-task T-cell landscape it represents roughly 23% of methods, because deep learning is the dominant paradigm in MHC binding, immunogenicity, and TCR specificity.

Deep Learning architectures have transformed epitope prediction through automatic learning of hierarchical feature representations from raw sequence data, and this capability eliminates the need for manual feature engineering. **Figure 12** reveals a striking specialization pattern where deep learning approaches constitute the dominant methodology in conformational B-cell epitope prediction with 6 of 13 methods (46%), maintain significant presence in linear B-cell epitope prediction with 7 of 32 approaches (22%), and become the *dominant* paradigm in T-cell prediction with 64 of 103 methods (62%). The pMHC-binding predictors [NetMHCpan-4.1 (49), MHCflurry 2.0 (50), BVLSTM-MHC (53)], immunogenicity models [DeepImmuno (64), ImmunoStruct (66)], and TCR–peptide specificity methods [TITAN (71), NetTCR-2.0 (68), ERGO-II (70), pMTnet (130), epiTCR (72)] make extensive use of CNN, LSTM, bimodal attention, graph neural network, transformer, and protein-languagemodel architectures, as detailed in Section 5, with the protein-language-model predictors among them analysed in Section 9. Multi-Layer Perceptrons (MLP) serve as the foundational deep learning architecture and consist of multiple layers of interconnected neurons with non-linear activation functions that demonstrate particular success in conformational B-cell epitope prediction for capturing complex spatial relationships between amino acid residues. Convolutional Neural Networks (CNN) and their variants including dilated 1-D CNNs excel at identifying sequential motifs and local dependencies within amino acid sequences, and the parameter sharing inherent in CNN architectures provides translation invariance that enables detection of epitope patterns regardless of their position within the protein sequence. Recurrent architectures including LSTM and Bidirectional LSTM have been specifically designed to capture sequential dependencies by maintaining memory of previous sequence elements to model long-range dependencies crucial for epitope recognition. Specialized architectures include Temporal Convolutional Networks that combine benefits of CNNs and RNNs for sequence modeling, ResNet-based approaches that employ skip connections for training very deep networks, and Bayesian Neural Networks that quantify prediction uncertainty for downstream applications. Beyond architecture, the training paradigm can itself be adapted to the data: in antigen processing (T2), positive-unlabeled (PU) learning has been used to train deep classifiers when confirmed negatives are unavailable, and it treats unlabeled peptides as a mixture of positives and true negatives rather than as confirmed non-cleavage sites (47).

Ensemble methods combine predictions from multiple base classifiers to achieve superior performance compared to individual models and show application with 2 approaches in linear B-cell epitope prediction and 7 across the ten T-cell sub-tasks, while remaining absent in conformational B-cell epitope prediction. Within T-cell prediction, ensembles concentrate in tumor antigen classification (T9), where stacked tree-based voting predominates, and in neoantigen prioritisation (T8). The T-cell ensemble approach integrates Random Forest, XGBoost, LightGBM, CatBoost, and Extra Trees Classifier through voting mechanisms to leverage diverse inductive biases of different tree-based algorithms. The ensemble paradigm follows two primary strategies that involve homogeneous ensembles combining multiple instances of the same algorithm trained on different data subsets, and heterogeneous ensembles integrating diverse algorithms with complementary strengths. Voting mechanisms employ either hard voting via majority class prediction or soft voting using average prediction probabilities, and soft voting generally provides superior performance when base classifiers output well-calibrated probabilities. The effectiveness of ensemble approaches stems from their ability to reduce both bias and variance components of prediction error, and this leads to more robust and generalizable models where diversity among ensemble members ensures that different aspects of the epitope prediction problem are captured.

Hybrid methodologies represent the fastest-growing category and combine multiple algorithmic paradigms to leverage complementary strengths across all prediction tasks with 7 approaches in linear B-cell, 3 in conformational B-cell, and 7 across the ten T-cell sub-tasks. Deep learning hybrids dominate this category with combinations such as CNN+MLP, CNN+BiLSTM+MLP, and CNN+TCN+MLP that typically employ CNNs for local feature extraction followed by fully connected layers for final classification or integrate recurrent components to capture sequential dependencies. The CNN+BiLSTM+MLP combination has proven particularly effective for linear B-cell epitope prediction where both local motifs and sequential context contribute to recognition. Machine learning hybrids in T-cell prediction combine traditional algorithms such as SVM+RF+XGBoost through different algorithms for different pipeline stages or voting mechanisms for output combination. CLBTope exemplifies cross-paradigm integration by combining dipeptide composition features with BLAST similarity searches to achieve hybrid approaches that outperform single-method solutions (2). Multi-stage architectures such as ResNet-1D+MLP employ sophisticated deep learning for feature extraction followed by simpler classifiers for final prediction, and this approach enables capture of complex patterns while maintaining computational efficiency.

The distribution of classifier categories across tasks reveals distinct methodological preferences that reflect underlying biological and computational considerations as shown in **Figure 12**. Conformational B-cell epitope prediction demonstrates strong preference for deep learning approaches at 46% of methods, and this reflects the complex spatial relationships that require sophisticated pattern recognition capabilities for three-dimensional structural analysis. Linear B-cell epitope prediction shows the most diverse classifier landscape with machine learning methods representing 47% and hybrid approaches representing 22%, and this distribution indicates that many linear epitope patterns can be effectively captured using established algorithms with appropriate feature engineering while the field actively explores more sophisticated methodologies. T-cell epitope prediction, taken across all ten sub-tasks, is dominated by deep learning (62%), with machine learning (23%) concentrated in the tumor T-cell antigen sub-task (T9) and efficient TCR predictors. Across the binding, immunogenicity, and TCR-specificity sub-tasks, CNN, LSTM, attention, transformer, graph-neural-network, and protein-language-model architectures predominate. Conversely, deep learning has only recently entered TTCA classification (T9) through iTTCA-DNN and LYnet, and its still-nascent adoption there, together with the absence of any dedicated protein language model predictor, remains a concrete opportunity for methodological advancement. Similarly, ensemble methods show zero adoption in conformational B-cell epitope prediction where their ability to combine diverse structural and sequence-based features could provide substantial benefits. Statistical models remain absent in conformational B-cell prediction, and this gap suggests potential for probabilistic approaches that could quantify uncertainty in structure-based predictions. The hybrid approach category demonstrates balanced cross-task adoption, and this suggests that integration of multiple algorithmic paradigms provides robust solutions applicable across different epitope prediction challenges.

To complement this paradigm-level view, the representative predictors deployed across the ten T-cell sub-tasks can be enumerated in cascade order, and this per-sub-task catalogue makes explicit that every sub-task is served by dedicated models. HLA typing and imputation (T1) is served on the machine-learning side by three imputation models, namely the adaptive hidden Markov model CookHLA (40), the multitask convolutional network DEEP-HLA (41), and the Transformer HLARIMNT (42), whereas the complementary read-based typing route remains predominantly statistical or alignment-based and is represented by HLA-HD (43), HLAminer (144), HISAT-genotype (44), and the maximum-likelihood imputation of HaploSFHI (145). Antigen processing (T2) adds the open-source C-terminal cleavage predictor NetCleave, which spans both class I and class II processing (46), together with antigen-processing-likelihood scoring extended to class-II epitopes [APLSuite (48)]. MHC class I binding (T3) ranges from CNN encoders [DeepMHC (146), ConvMHC (147)] and classical multitask support-vector machines (148) to per-position structure-based scoring [3pHLA (55)], variable-length composition models [DPCMHC (54)], and the global–local heterogeneous bidirectional-LSTM regression model DeepNeoAGNet (149). It also includes the anchor-motif deconvolution predictor MixMHCpred (56) and the deep-learning presentation and immunodominance model MUNIS, which is trained on more than 650,000 HLA class I ligands and surpasses NetMHCpan-4.1 and MHCflurry 2.0 in average precision (57). MHC class II binding (T4) progresses from trans-allelic (150) and pan-specific binding models (151, 152) to LSTM-attention networks [DeepSeqPanII (60)], multi-task transfer learning [MTL4MHC2 (61)], structure-aware transformers (153), and the MixMHC2pred predictors that build on probabilistic motif deconvolution (59, 154). Immunogenicity (T5) covers logistic-regression contact models [PRIME (63)] and contact-potential tree ensembles [Repitope (65)], RNN and attention networks [DeepHLApan (67)], CNN and generative models [DeepImmuno (64)], stacked-ensemble predictors [NeoTImmuML (155)], CNNs with molecular-dynamics features (156), structure-based and multimodal frameworks [the model of Riley et al. (157), ImmunoStruct (66), and the clonality-aware NeoPrecis (158)], and transformer transferlearning (17) and protein-therapeutic risk models (159), the protein-language-model-based CD8^+^ epitope workflow of Lee et al. (21), and the physicochemical immunogenicity baseline of Calis et al. that underpins the IEDB immunogenicity tool (104), with the reported performance ceiling analysed by Carri et al. (160). TCR-peptide-MHC specificity (T6) further adds NetTCR-2.1 (69), TcellMatch (161), ATM-TCR (162), meta-learning [PanPep (163)], active learning [ActiveTCR (164)], explainable machine learning [TCR-H (165)], explain-by-design protein-language-model layers [TCR-EML (166)], dictionary-based models (167), the unified cross-attention transformer UnifyImmun that jointly scores peptide-HLA and peptide-TCR binding (77), and the cross-attention triad sequencefusion transformer of Ma et al. (168). Structural TCR modelling (T7) is served by four architecture families: the specialised AlphaFold2 docking pipelines TCRdock (80), which constrains the docking orientation with hybrid structural templates and runs without multiple-sequence-alignment input, and TCRmodel2 (20), which uses TCR- and MHC-focused alignments with custom peptide-MHC template parameterisation; the ESM-IF1-augmented geometric-vector-perceptron graph neural network NetTCRstruc (81), which scores docking-pose quality; and the coordinate-level CNN-plus-LSTM refinement followed by a three-dimensional CNN binding classifier in TCRcost (82). Beyond these four, the structural toolkit for this sub-task also includes dedicated rapid immune-receptor structure predictors such as ImmuneBuilder and its TCRBuilder2 module (83), together with the general structural foundation model AlphaFold3 that now sets the state of the art for biomolecular complex prediction (84). Neoantigen identification (T8) spans the three framings introduced in Section 5. Its prediction-and-prioritisation framing combines classical machine learning, namely the random forest of pTuneos (85) and the logistic-regression model NeoGuider (86) with its adaptive kerneldensity and isotonic-regression feature transforms, together with deep neural pipelines, namely the mass-spectrometry-trained fully connected network TruNeo (87), the multimodal language-model pipeline CNNeoPP (88), the alternative-splicing pipeline ASNP (169) that reuses the DeepHLApan recurrent network, and the recognition-potential pipeline NeoPredPipe (89). Its annotation-andvisualisation framing contributes the feature-annotation toolbox NeoFox (90) and the interactive prioritisation interface pVACview (91), neither of which trains a predictor of its own. Its reactive-T-cell framing is represented by the gradient-boosting (XGBoost) classifier of Shi et al. (142) that identifies neoantigen-reactive CD8^+^ T cells from single-cell transcriptomes. These tools are evaluated on validated neoantigen sets such as that of Verdegaal et al. (170). A complementary framing scores neoantigen quality through immune-fitness models that estimate TCR-recognition potential and dissimilarity from self, an approach introduced by Luksza et al. (171) and Balachandran et al. (172) that now underlies the recognition-potential pipelines named above. Multi-epitope vaccine design (T10) is dominated by classical approaches and divides into two framings: reverse-vaccinology whole-protein antigen discovery, served by the gradient-boosting predictors Vaxign-ML (101) for viral proteomes and SHASI-ML (102) for bacterial antigens, together with the protein-language and geometric deep learning antigen predictor PLGDL (103), and epitope-construct assembly, served by the deep neural network DeepVacPred (100) that couples a convolutional and a dense sub-network to score and concatenate B-cell and T-cell epitope vaccine subunits. Tumor T-cell antigen classification (T9), by contrast, relies on the classical machine-learning and stacked-ensemble predictors already discussed above. The full per-study mapping of these predictors to their representation learning, classifier family, performance, and code availability is provided in **Supplementary Table 4**.

## 9 Protein language models and large language models in epitope prediction

Protein language models (PLMs) and large language models (LLMs) constitute a cross-cutting paradigm in epitope prediction, because a single transformer can serve at once as a sequence encoder and as a classifier and therefore spans both stages of the predictive pipeline analysed in the previous section. This dual role warrants a dedicated treatment that follows the languagemodel thread across all twelve tasks rather than dividing it between the representation and classifier categories. Transformer-based protein language models are now established across both the B-cell and T-cell branches, although the depth of adoption varies markedly from one task to the next.

In B-cell epitope prediction, the ESM family of protein language models has become the dominant modern encoder. Conformational predictors are the most advanced adopters: BepiPred-3.0 (38), SEMA and SEMA 2.0 (15, 120), EpiCluster (6), GraphBepi (37), DiscoTope-3.0 (36), and CBTOPE2 (39) build on ESM-2, ESM-1v, ESM-IF1, SaProt, or AlphaFold2 structural embeddings, and they frequently couple them with graph neural networks or inverse-folding representations. In linear B-cell prediction, BERT and ELMo embeddings drive LBCE-BERT and LBCE-XGB (8, 10) and EpiDope (34), and single-sequence ESM embeddings appear in recent predictors (173). EpiBERTope, a sequence-based pre-trained BERT model, captures long-distance protein interactions and improves both linear and conformational epitope prediction (35). In **Figure 11**, the language-model category accounts for eight encodings across the two B-cell tasks. Several further conformational predictors that the figure classifies as structural or graph methods also build on ESM embeddings, so the overall reach of protein language models in B-cell prediction is wider than this single category suggests.

In T-cell prediction, the adoption of protein language models is more extensive than in either B-cell task. Across the ten T-cell sub-tasks, the language-model category of **Figure 11** reaches twenty-three encodings, which is nearly three times the B-cell total. MHC class I binding (T3) draws on pretrained ESM and BERT embeddings as well as bespoke transformer encoders, which appear in BigMHC (52), the interpretable BERT model of Gasser et al. (174), the transformer-based TransPHLA (51), the ESM2-3B domain-adapted encoder of Hashemi et al. (175), the ESM1baugmented presentation model HLApollo (176), and the multimodal CMHS model that combines LoRA-tuned ESM2 with ESM3 structural embeddings (177). MHC class II binding (T4) adopts ESM2-based multi-scale models (178). The class-II predictor BERTMHC fine-tunes a pretrained transformer with multiple-instance learning, and its attention weights expose the anchor positions of the binding core (62). Antigen processing (T2) has only begun to probe these models, because a benchmark of ESM2 and ProtT5-XL embeddings for proteasomal cleavage found that a simpler bidirectional LSTM matched or exceeded them (129). Immunogenicity prediction (T5) applies transformer architectures with cross-source transfer learning (17). The richest concentration is in TCR–peptide-MHC specificity (T6), where TCR-ESM (74), TULIP (73), STAPLER (78), TCR-BERT (22), TEPCAM (24), the repertoire-pretrained context-aware embedding model catELMo (79), and the triad sequence-fusion transformer of Ma et al. (168) use ESM, BERT, ELMo, or bespoke transformer language models. HLA imputation (T1) has begun to adopt the transformer architecture, where HLARIMNT applies multi-head self-attention with positional encoding to the chunked SNP sequence (42), although it is trained from scratch on genotype data rather than built on a pretrained protein language model. AlphaFold2 functions as a structural foundation model that underpins both conformational B-cell prediction and structural TCR modelling (T7) (20, 80), and within T7 the inverse-folding language model ESM-IF1 supplies structural node embeddings to the graph-based docking-quality scorer NetTCRstruc (81).

Several distinct modes of PLM utility recur across the tasks, and they range from the simplest reuse of pretrained features to dedicated pretraining on immune-receptor data. The first mode uses frozen PLM embeddings as input features to a downstream classifier. This is the most common pattern, and it is exemplified by the ESM-based conformational B-cell predictors, by TCR-ESM, and by the frozen ProteinBERT, ESM-1b, and ESM-2 features that the explain-by-design head of TCR-EML consumes (166). The second mode fine-tunes or pre-trains a transformer end-to-end on epitope or receptor data, as in TULIP, STAPLER, the BERT-based linear B-cell models, and the class-II predictor BERTMHC (62). The third mode adapts a large pretrained model through parameter-efficient fine-tuning rather than full retraining, as in CMHS, which couples low-rank adaptation of ESM2 with ESM3 structural embeddings (177). The fourth mode supplies structural or inverse-folding representations rather than sequence embeddings alone, and it is exemplified by the ESM-IF1 node features of NetTCRstruc (81) and by AlphaFold2 and AlphaFold3, which serve as structural foundation models for conformational B-cell prediction and structural TCR modelling (20, 80, 84). The fifth mode performs self-supervised pretraining directly on large unlabelled T-cell-receptor repertoires, as in catELMo, which learns context-aware amino-acid embeddings from more than four million receptor sequences and improves downstream binding prediction over earlier encodings (79). The sixth mode exploits PLM attention for interpretability, as in the interpretable MHC-binding model of Gasser et al. (174), and this mode remains the least developed despite its clear value for mechanistic understanding and clinical acceptance. The principal remaining gaps are threefold. First, PLM adoption is still uneven across the cascade. No dedicated predictor for tumor T-cell antigen classification (T9) yet uses a protein language model, although general peptide frameworks have already applied ESM-2 embeddings to the TTCA benchmark, as in UniDL4BioPep (141), so the gap there is one of purpose-built adoption rather than feasibility. Multi-epitope vaccine design (T10) has only very recently seen its first protein-language-model application, namely the PLGDL framework that fuses a protein language model with geometric deep learning to rank protective vaccine antigens, so its use of these models is emerging rather than established (103). Neoantigen identification (T8) is only beginning to incorporate them, and CNNeoPP fine-tunes a BioBERT language model on peptide and HLA sequences as an early exception to the otherwise feature-engineered tools of that sub-task (88). Second, the computational cost of large PLMs creates accessibility barriers for smaller groups. Third, systematic and fair comparison of PLM-based against traditional encodings under identical evaluation protocols is still rare, and where such comparison has been carried out, as in a recent benchmark of nineteen T-cell-receptor embedding methods, hand-crafted encodings sometimes matched or exceeded data-driven language-model embeddings (127). These gaps define the genuine frontier for language-model methods in epitope prediction.

## 10 AI-empowered predictive pipelines performance on benchmark datasets

This section synthesizes performance patterns achieved by epitope predictors across benchmark datasets for linear B-cell, conformational B-cell, and T-cell epitope prediction. The accompanying tables and figures provide detailed numerical results, and therefore this section focuses on critical patterns, evaluation gaps, and optimization strategies that emerge from our comprehensive analysis of the 148 predictive models surveyed (with per-sub-task T-cell performance consolidated in **Table 2**).

### 10.1 Linear B-cell epitope predictors performance landscape

The performance landscape for linear B-cell epitope prediction reveals fundamental inconsistencies that challenge the field’s maturity claims. **Supplementary Table 2** documents AUC scores spanning from 0.527 to 0.990, and this extreme variability reflects not only methodological differences but also critical evaluation fragmentation. Most studies evaluate on only one or two datasets despite the availability of multiple public benchmarks, and this selective approach prevents assessment of generalization capability. Liu et al. (10) represents a notable exception through testing across six datasets, and their results expose the danger of single-dataset evaluations: the same BERTbased method achieved 99.0% AUC on the Chen dataset but dropped to 54.7% AUC on LBtope. Such dramatic cross-dataset variation demonstrates that reported peak performance on individual benchmarks provides misleading estimates of practical prediction capability. The benchmarking culture exhibits systematic deficiencies that undermine comparative assessment. Eight out of 32 studies provide no comparative evaluation with existing methods, and others compare against only 2–3 baseline approaches. Kumar et al. (2) conducted the most comprehensive benchmarking effort with comparisons against 11 methods, yet such rigor remains exceptional rather than standard practice. The comparison deficit perpetuates claims of superiority based on insufficient evidence and prevents the field from establishing reliable performance baselines.

Representation learning effectiveness analysis identifies clear performance patterns across different feature engineering approaches. BERT-based representations demonstrate superior performance when rigorously evaluated, with Liu et al. (8, 10) achieving consistently high AUC values above 0.83 across multiple datasets. Traditional physicochemical property approaches show variable results, with Amaya et al. (179) study reaching 98.5% AUC using hydrophobicity and electrostatic features, while similar approaches in other studies achieve modest performance around 0.75 AUC. Sequence-based methods display the widest performance variance, from Lim et al. (106) achieving 99.1% accuracy with k-mer composition to other sequence approaches struggling below 0.8 AUC. Graph-based representations employed by Da et al. (9) achieved strong performance with 92.0% AUC on the BCPred dataset, which suggests that structural feature encoding captures relevant epitope properties that are often missed by sequence-only approaches. Classifier performance analysis reveals that ensemble methods and Random Forest consistently outperform single classifiers across multiple studies. Random Forest appears in 8 studies with generally strong results, including Da et al. (9) achieving 92.0% AUC and Sahu et al. (115) reaching 91.35% AUC. XGBoost demonstrates effectiveness in combination with BERT features, as shown by Liu et al. (10) study exceptional performance. Support Vector Machine performance varies dramatically depending on feature quality, from excellent results in some studies to poor performance in others. On the other hand, deep learning approaches show inconsistent results that depend heavily on feature quality and dataset characteristics (34, 115).

For instance, BERT-based methods achieve great performance while other neural network implementations like Collatz et al. (2020) (34) achieved only 62.5-67.0% AUC. Independent validation analysis exposes systematic overfitting concerns across the field. Studies employing both cross-validation and independent testing consistently show performance degradation on held-out data. Qi et al. (7) demonstrated drops from 77% cross-validation accuracy to 60-64% on independent datasets, and only 15 out of 32 studies conducted proper independent validation. The prevalence of private datasets further constrains reproducibility, with 12 out of 32 studies using private data exclusively. Studies on private datasets often report substantially higher performance metrics, and this pattern suggests potential bias toward favorable data that may not represent realworld prediction challenges (116, 179). Temporal analysis from 2018 to 2024 reveals concerning stagnation in fundamental performance improvements. Despite methodological innovations including transformer models and advanced feature engineering, average performance metrics show no clear upward trajectory. Studies from 2024 do not consistently outperform those from 2020-2021, and this observation indicates that current approaches may have reached performance plateaus without addressing core biological complexity. Critical methodological gaps further constrain practical application. No studies provide uncertainty quantification methods essential for clinical decision support, feature interpretability analysis remains minimal, and correlation with experimental binding data or clinical outcomes remains extremely limited.

### 10.2 Conformational B-cell epitope predictors performance landscape

The evaluation of conformational B-cell epitope prediction presents even greater complexity than linear b-cell epitope predictors. This complexity is characterized by significant heterogeneity in metrics utilization and dramatic dataset-dependent performance variability. The treemap visualization in **Figure 13** reveals that AUC emerges as the dominant evaluation metric in 12 out of 13 studies, followed by MCC in 8 studies and F1 score in 7 studies. The comparison thoroughness panel in **Figure 13** reveals substantial variation in baseline comparison rigor across studies. Singh et al. (14) conducted the most extensive comparison against 14 baseline methods including DiscoTope, BEpro, ElliPro, COBEpro, SEPPA 3.0, EPITOPIA, EPCES, BEST, Bpredictor, EPMLR, EpiDope, iBCE-EL, and fuzzy SVM variants. Whereas, Wang et al. (123) compared against 11 methods across 8 datasets. However, the most critical benchmarking study performed by Cia et al. (119) established a sobering baseline by evaluating nine state-of-the-art webservers on 268 representative antibody-antigen structures. They found that all methods achieved very low performance with AUC-ROC and balanced accuracy values below 0.6 and MCC values below 0.1, and some methods performed no better than randomly generated patches of surface residues. Furthermore, performance distribution across 27 unique datasets from 13 studies shown in Bar graph of **Figure 13** reveals striking variability ranging from 0.213 to 0.989 when considering mixed evaluation metrics. The highest reported performance of 0.989 AUC was achieved by DL-TCNN (4) on the Ansari and Raghava 2010 benchmark dataset, yet these results were obtained after SMOTE balancing on a relatively small dataset of 57 antigen chains. The lowest performance was observed on DiscoTope3 datasets evaluated by Wang et al. (123), with AUC-PR values of 0.213, 0.217, and 0.193 for solved, Foldx-optimized, and AlphaFold-generated structures respectively. This variation exceeds what would be expected from method differences alone and suggests that prediction difficulty may be fundamentally dataset-dependent rather than method-dependent.

**Figure 13:**
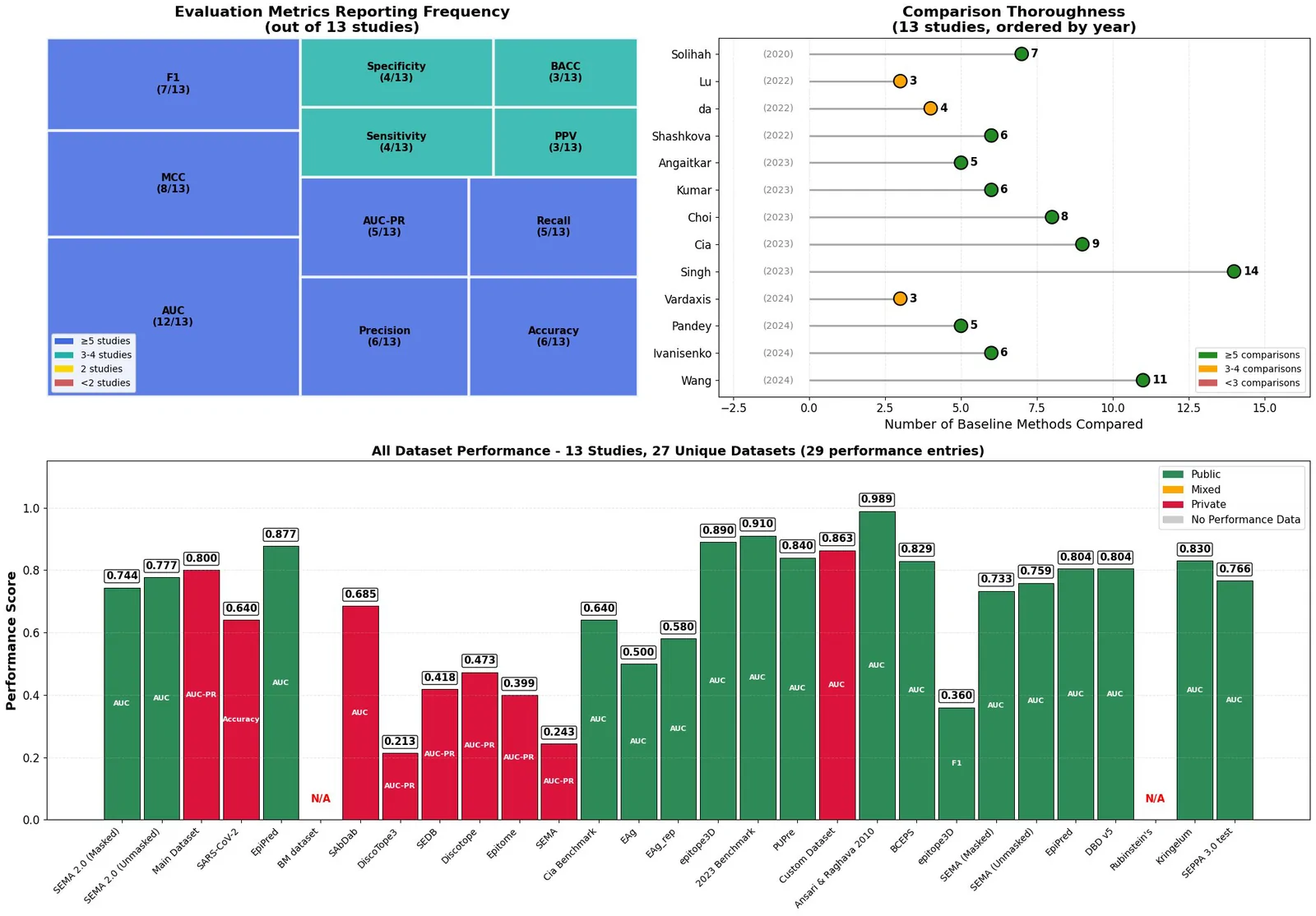
A summary of conformational B cell epitope prediction performance landscape. Performance metrics shown derive from heterogeneous datasets and evaluation protocols across individual studies and are not directly comparable across methods.

The influence of data preparation strategies on reported performance is evident in multiple studies. Ivanisenko et al. (15) designed masked test set to focus on protein regions that are in close proximity to experimentally determined antibody contacts while excluding uncharacterized regions where annotations might be incomplete. This design enables more rigorous evaluation of the model’s ability to delineate epitope boundaries. The superior performance of Ivanisenko et al. (15) SEMA 2.0 predictor on the masked test set demonstrates the capability of identifying conformational epitope boundaries rather than simply distinguishing epitopes from distant regions. In a related advance, the EpiCluster method (6) achieved state-of-the-art results by explicitly modeling the clustering property of epitopes. Their ablation study revealed that the explicit enforcement of epitope clustering through end-to-end training provides substantial performance gains beyond simple feature concatenation. It is important to highlight that multi-metric evaluation reveals concerning patterns where high AUC-ROC values mask significant practical limitations. For example, Wang et al. (123) conducted the most comprehensive multi-dataset evaluation by testing their WUREN method across eight different benchmark datasets, and their results showed AUC-ROC of 0.877 but substantially lower precision of 0.359 on the EpiPred benchmark. Overall, the observation from Cia et al. (119) that each method predicts some proteins quite well and others very poorly, with methods generally not agreeing on which proteins are easy or difficult, suggests that individual antigen characteristics may dominate over algorithmic differences.

### 10.3 Performance across the broader T-cell sub-tasks (T1–T10)

Turning from the B-cell tasks to the broader T-cell landscape, the performance of the ten T-cell subtasks (T1–T10) is summarised per sub-task in **Figure 14**, with the underlying per-study figures in **Supplementary Table 4** and the consolidated view in **Table 2**. As with the B-cell tasks, these values derive from heterogeneous datasets, splits, and evaluation protocols and are therefore not directly comparable across sub-tasks. Several robust patterns nonetheless emerge. MHC class I binding (T3) and HLA typing (T1) are the most mature, with best-reported AUC and concordance values frequently above 0.9, whereas MHC class II binding (T4) trails class I (approximately 0.8–0.9) because of DQ/DP data scarcity and binding-core ambiguity. TCR–peptide-MHC specificity (T6) spans the widest range (approximately 0.6–0.98), and the high end is achieved only on *seen* epitopes, since the IMMREP22 benchmark and NetTCR-2.1 both document a steep collapse on unseen epitopes. Immunogenicity prediction (T5) shows the lowest ceiling, with AUC values that plateau near 0.6– 0.8 and that propagate as the principal bottleneck into neoantigen identification (T8). Neoantigen pipelines (T8) themselves report high prioritisation AUCs, up to approximately 0.99 when validated neoantigens are contrasted with random unmutated or unexpressed background peptides, but the operationally decisive quantity is the immunogenic fraction among the top-ranked candidates, and this fraction stays low because it inherits the immunogenicity ceiling of T5, so a typical pipeline recovers only a handful of immunogenic peptides within its top ten to fifty ranked candidates. Structural TCR modelling (T7) reaches high recognition AUC-ROC, up to approximately 0.97, on well-characterised epitopes and precise experimental structures, but this figure falls to roughly 0.76 on realistic AlphaFold-derived structures and lower still on unseen peptides, and AlphaFoldbased modelling attains medium-or-high docking accuracy under the CAPRI criteria in only about half of benchmark cases, so the small number of experimentally solved complexes remains the central constraint. Multi-epitope vaccine design (T10) reports high in-silico accuracies that reflect construct-level simulation rather than wet-lab efficacy. The recurring lesson across sub-tasks is that headline metrics obtained on seen, in-distribution data substantially overstate the performance that is achievable under realistic, unseen-epitope or cross-population conditions.

**Figure 14:**
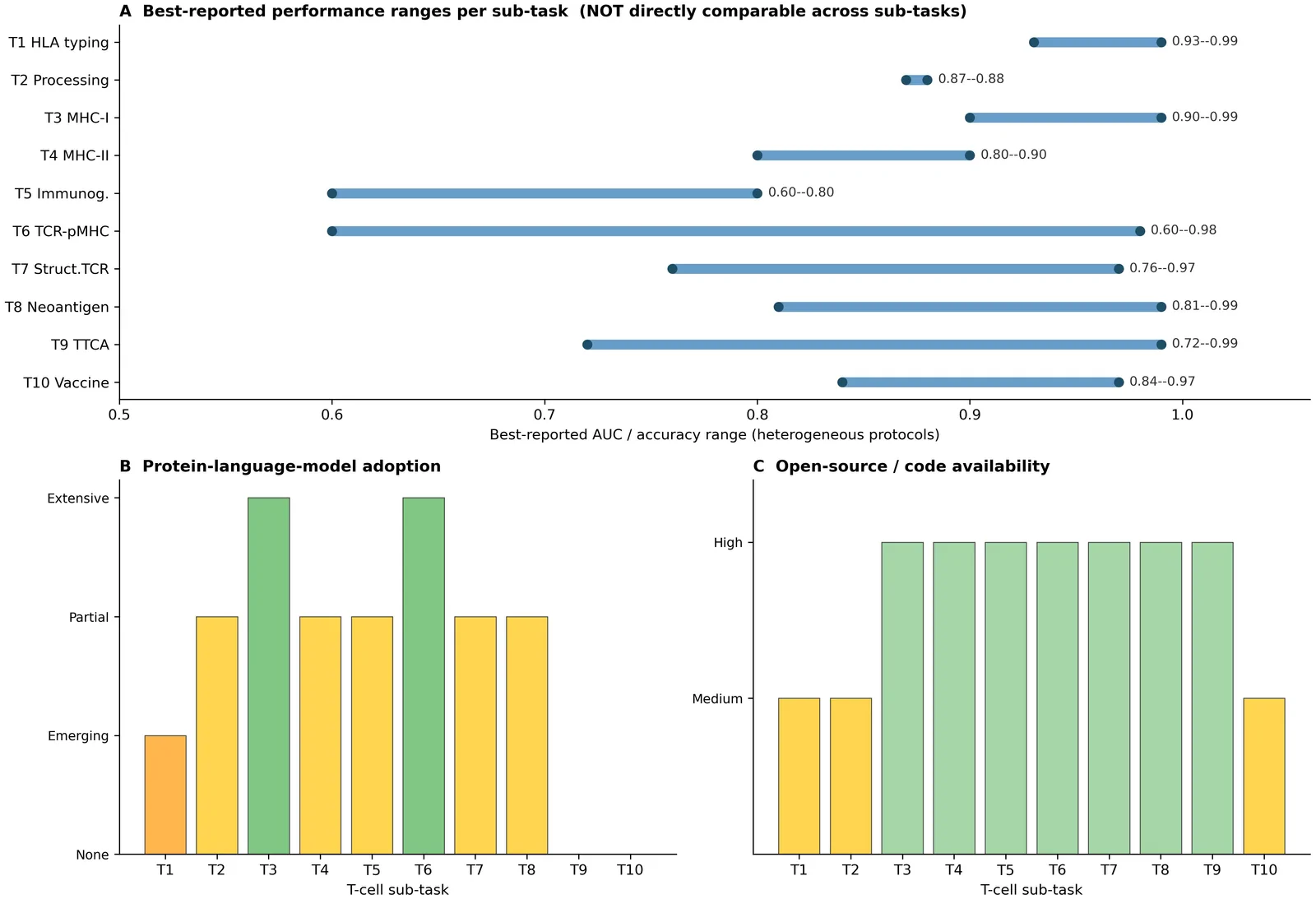
Performance, protein-language-model adoption, and code availability across the ten T-cell prediction sub-tasks (T1–T10). **(A)** Bestreported AUC/accuracy ranges per sub-task. These values derive from heterogeneous datasets and evaluation protocols and are *not* directly comparable across sub-tasks. **(B)** Protein-language-model adoption level. **(C)** Open-source or web-server code availability. Underlying perstudy values are listed in **Supplementary Table 4**.

### 10.4 Tumor T-cell antigen (T9): performance landscape

Among the ten T-cell sub-tasks, tumor T-cell antigen classification occupies a distinctive position. It is a downstream translational application in the same family as multi-epitope vaccine design, because its predictions feed directly into cancer-vaccine and immunotherapy design, and it is at the same time the sub-task whose public benchmarks are the most completely characterised. This pairing of high clinical impact with full data transparency makes it the review’s running case study, first introduced in Section 5, and an ideal setting for examining how far sequence-only prediction can be pushed. The picture that emerges is a mature data landscape set against an immature methodological one. Performance has plateaued under classical machine learning, deep learning has only recently appeared through iTTCA-DNN (98) and LYnet (99), and protein language models have so far been tested only indirectly through general peptide frameworks. The sub-task therefore carries substantial unrealised headroom for diverse protein language models and modern representation learning.

A comprehensive tumor T-cell antigen (TTCA) performance analysis across fourteen predictors, thirteen of which have public benchmark results shown in **Figure 15** (the private TTAgP 1.0 is excluded), reveals concentrated evaluation efforts on limited datasets and significant methodological variations (the performance of the broader T-cell sub-tasks is summarised above and consolidated in **Table 2**). **Figure 15** presents seven key analytical perspectives that collectively illustrate the current state of tumor T-cell antigen prediction research and highlight critical limitations constraining clinical translation. Dataset coverage analysis demonstrates that the Charoenkwan dataset remains the most widely used and appears in eight studies once the deep-learning entrants are included, followed by the Herrera dataset in six studies, while the Sotirov and Hassan datasets are used by one study each (136–139). The two deep-learning predictors iTTCA-DNN and LYnet are summarised in the dedicated deep-learning panel, because iTTCA-DNN is evaluated on the Charoenkwan benchmark whereas LYnet is evaluated on a merged set re-derived from the Charoenkwan, TAP 1.0, and iTTCA-RF data. Overall, no study conducted systematic evaluation across all three core public datasets. This fragmented evaluation prevents comparative assessment of predictor generalization and robustness. Cross-validation performance consistently exceeds independent test results across all TTCA datasets with systematic improvements of 5-15%, and this pattern indicates potential overfitting tendencies but maintained reasonable generalization capability. The Charoenkwan dataset demonstrates more variable performance with wider accuracy ranges of 72-95%, while the Herrera dataset shows more consistent high performance levels across different methodological approaches at 74-93%. External validation analysis reveals extremely limited evaluation beyond core benchmark datasets, with only two studies performing systematic external validation. This constraint severely limits assessment of practical clinical translation potential and real-world application effectiveness.

**Figure 15:**
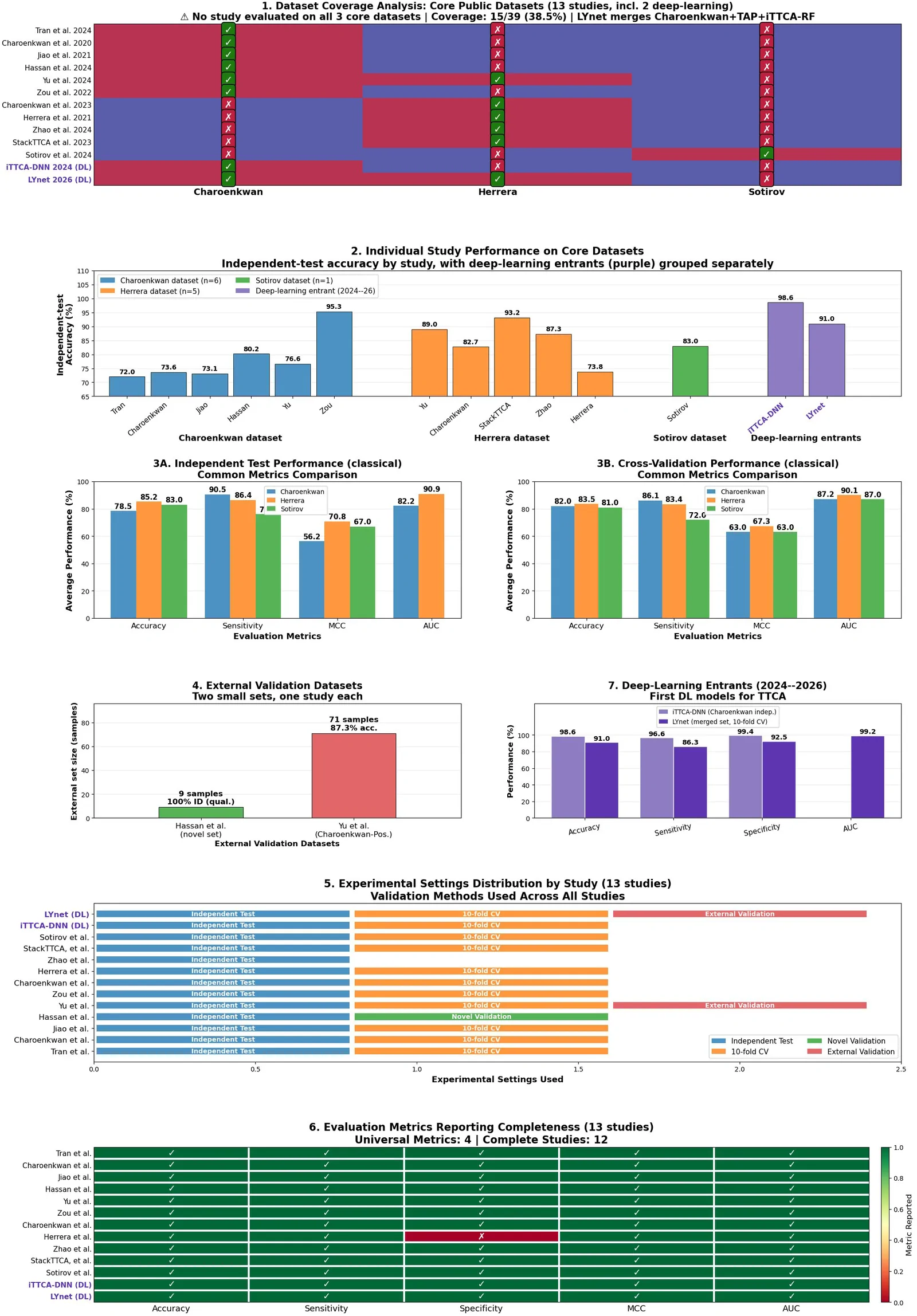
A summary of the tumor T-cell antigen (TTCA) prediction performance landscape. Performance metrics shown derive from heterogeneous datasets and evaluation protocols across individual studies and are not directly comparable across methods. Performance of the broader T-cell sub-tasks is summarised per sub-task in **Table 2**.

Within this classical machine learning landscape, StackTTCA (96) reports the strongest overall accuracy, robustness, and usability among the ensemble predictors. The first deep-learning entries have now appeared and shift this picture. The deep neural network iTTCA-DNN (98) reports 99.02 percent accuracy on cross-validation and 98.62 percent on the independent test of the Charoenkwan benchmark, and the hybrid convolutional and bidirectional LSTM model LYnet (99) reports a cross-validation AUC of 0.992 together with independent AUCs of 0.949 on the TAP 1.0 set and 0.836 on the iTTCA-RF set, which exceed the strongest prior method by 2.4 to 6.9 percentage points of AUC. These figures should be read against the caveats above, because both models inherit the benchmark and negative-set limitations that affect the whole sub-task. Protein language models have not yet been adopted by any dedicated TTCA predictor, but they have already been applied to TTCA benchmark data through two general peptide frameworks that are not TTCAspecific tools. UniDL4BioPep (141) fits ESM-2 embeddings with a convolutional head to twenty bioactivity datasets, one of which is the TTCA benchmark, and PepBenchmark (135) evaluates fingerprint, graph, protein-language-model, and chemical-language-model families on a standardised TTCA split. These two frameworks therefore demonstrate that protein language model embeddings can be applied to TTCA data, even though no purpose-built TTCA predictor yet uses them, which marks the clearest methodological opening for the sub-task.

Accessibility and reproducibility remain uneven across the sub-task. Of the predictors that offer a public interface, only a minority were ever released as web servers, and several of these are no longer reachable, so that the iTTCA-RF, PSRTTCA, and StackTTCA servers remain the dependable points of access while earlier servers such as those of iTTCA-Hybrid and TAP 1.0 have lapsed. The more recent deep-learning and benchmark contributions instead distribute open source code and data through public repositories, as with LYnet, UniDL4BioPep, and PepBenchmark, which is a more durable basis for reuse and independent verification. This shift from short-lived web servers toward versioned open repositories is a practical prerequisite for the cumulative progress that the sub-task still lacks. These deployment patterns are audited in full, and set against the broader accessibility landscape of the twelve tasks, in Section 11.

Critical methodological gaps emerge from this comprehensive analysis that constrain practical application potential. No studies provide confidence estimation methods that would enable uncertainty quantification essential for clinical decision support applications. Feature interpretability analysis remains minimal across all investigations. This limits mechanistic understanding of computational model decision patterns and reduces clinical acceptance potential. Systematic crossdataset evaluation approaches are completely absent, with only Yu et al. (97) testing across multiple datasets but achieving lower performance on the more challenging Charoenkwan dataset (137) compared to Herrera dataset (136) evaluations. Most critically, correlation with experimental immunogenicity data or clinical outcomes remains extremely limited. This leaves practical relevance for therapeutic applications unvalidated and constrains translation from computational accuracy to clinical utility. These limitations indicate that tumor T-cell antigen prediction requires substantial methodological advancement in uncertainty quantification, interpretability analysis, and experimental validation correlation before achieving clinical translation readiness for therapeutic epitope discovery applications.

### 10.5 Evaluation and optimization strategies

Researchers have employed twenty-five distinct evaluation measures to assess the performance of predictive pipelines developed across the B-cell and T-cell prediction tasks, and these measures fall into four families. The first is a core set of twelve classification measures, namely Accuracy (ACC), Matthews Correlation Coefficient (MCC), Area Under the Receiver Operating Characteristic curve (AUC-ROC), Area Under the Precision-Recall curve (AUC-PR), Sensitivity (Sn), Specificity (Sp), F1score, Balanced Accuracy (BACC), Precision, Geometric Mean (Gmean), Brier score, and Kappa coefficient. The other three families extend this core, because several T-cell sub-tasks are not posed as simple binary classification: five regression and agreement measures, namely the Pearson and Spearman correlation coefficients, root mean square error, the coefficient of determination, and allele-resolution concordance; four ranking and enrichment measures, namely the early-enrichment partial measure AUC0.1, percentile rank, FRANK, and top-k recall and precision; and four structuralquality and confidence measures, namely DockQ, the CAPRI success categories, interface and backbone root mean square deviation, and the model-confidence scores given by the predicted aligned error and the predicted TM-score.

The B-cell tasks and the classical T-cell tasks operate mainly within the core classification family. Linear B-cell prediction emphasizes AUC-PR, F1-score, and MCC because of severe class imbalance, and conformational B-cell prediction emphasizes AUC-PR and MCC for structural imbalance, and Gmean appears in specific methods such as CluSMOTE. The cascade tasks then draw on the extended families according to their problem formulation. HLA typing and imputation (T1) reports concordance and accuracy at four-digit allele resolution together with sensitivity, positive predictive value, and the coefficient of determination of imputed allelic dosage, and it stratifies these by allele frequency so that the difficulty of rare alleles becomes explicit. Antigen processing (T2) prefers threshold-independent ranking because its negative labels are unreliable, so PUUPL reports a positive-unlabeled AUC-ROC with AUC-PR and NetCTLpan reports the high-specificity partial measure AUC0.1. MHC class I binding (T3) and MHC class II binding (T4) separate affinity regression, which is assessed with Pearson and Spearman correlation and root mean square error, from eluted-ligand presentation, which is assessed with AUC-ROC, AUC0.1, positive predictive value, percentile rank, and FRANK, and the class II methods add register-specific evaluation against crystal binding cores and stratify by locus to expose HLA-DQ and HLA-DP scarcity. Immunogenicity prediction (T5) reports AUC-ROC almost universally but treats AUC-PR and top-ranked precision as decisive because immunogenic peptides are rare, and it adopts leakage-resistant schemes such as leave-one-study-out, leave-one-allele-out, and leave-one-drug-out validation. TCR-peptide-MHC specificity prediction (T6) is judged chiefly by generalization to unseen receptors and unseen epitopes through random, TCR-hard, epitope-hard, and strict splits, and several methods treat MCC as more reliable than AUC-ROC on these imbalanced data. Structural TCR-pMHC prediction (T7) relies on DockQ, the CAPRI success categories, and interface and backbone root mean square deviation, and it uses the predicted aligned error and the predicted TM-score both to rank candidate models and to separate accurate from inaccurate complexes. Neoantigen identification (T8) is a prioritization problem, so top-k recall and precision and experimental validation rates dominate alongside AUC-PR, and the high values obtained on easy random-mutation negatives are distinguished from the low real-world immunogenic yield. Tumor T-cell antigen classification (T9) distinctively employs BACC and the Brier score for probabilistic assessment, and the Brier score appears mainly in Sa-TTCA. Multi-epitope vaccine design (T10) frames antigen selection as binary classification, so DeepVacPred reports AUC-ROC with accuracy, sensitivity, and specificity on balanced data, whereas the reverse-vaccinology classifier Vaxign-ML adopts the imbalance-aware weighted F1-score and MCC under nested cross-validation. The recurring lesson is that the evaluation vocabulary widens as the cascade moves from classification toward ranking and structural quality, and that across every family the decisive question is whether a measure is reported on seen, in-distribution data or under the unseen, out-of-distribution conditions that govern real-world utility.

Performance enhancement efforts target both feature selection and hyperparameter optimization of predictive models through approaches tailored to each task. Sixteen feature-selection strategies have been implemented across the tasks, namely Information Gain, Maximum Relevance Maximum Distance (MRMD), Incremental Feature Selection (IFS), Least Absolute Shrinkage and Selection Operator (LASSO), Pearson correlation, the Chi-Square test, the Boruta package, stepwise greedy selection, maximum relevance minimum redundancy (mRMR), Shapley Additive Explanation (SHAP), heuristic algorithms, ablation studies, graph-based ranking, multimodal feature fusion, recursive feature elimination, and tree-based importance ranking.

The defining pattern is a split between the deep-learning predictors that learn representations end to end and the classical pipelines that retain explicit feature engineering. Linear B-cell prediction frequently employs mRMR and stepwise greedy selection over sequence-derived and conservation features, and conformational B-cell prediction uses graph-based ranking, ablation studies, and multimodal feature fusion over three-dimensional structural properties. Along the cascade, HLA typing (T1), MHC class I binding (T3), MHC class II binding (T4), TCR-peptide-MHC specificity (T6), and structural TCR-pMHC prediction (T7) perform little or no explicit selection, because DEEP-HLA and HLARIMNT consume the MHC-region single-nucleotide polymorphisms within a fixed window, the binding and specificity predictors such as NetMHCpan-4.1 and NetTCR learn directly from BLOSUM encodings or pretrained embeddings, and the structural methods operate on AlphaFoldderived inputs and geometric graph features. Where SHAP, LIME, or attention maps appear in these tasks they support interpretation rather than selection, and ActiveTCR selects informative training pairs through active learning rather than input features. Genuine selection persists in a few methods, namely the trans-allelic class II model of Degoot and colleagues, which induces sparsity with an L1 penalty, and 3pHLA-score, which retains the informative peptide positions by ablation. Explicit engineering is most central to the classical sub-tasks. Immunogenicity prediction (T5) provides the clearest example in Repitope, which reduces hundreds of contact-potential features through correlation filtering and consensus random-forest importance ranking, whereas NeoPrecis applies recursive feature elimination. Neoantigen identification (T8) spans the same spectrum, because pTuneos relies on five curated immunogenicity features, NeoGuider admits a tested-candidate-count feature only after correlation testing, and the model of Shi and colleagues reduces the transcriptome to a twenty-six-gene signature by gradient-boosting importance. Tumor T-cell antigen classification (T9) depends heavily on Information Gain, MRMD with IFS, LASSO, the Boruta package, and SHAP over physicochemical descriptors. Multi-epitope vaccine design (T10) performs no classical selection, because DeepVacPred encodes peptides into a fixed vector and learns end to end, whereas Vaxign-ML and SHASI-ML let gradient-boosted trees rank the features internally. The practical implication is that explicit feature selection now characterizes the classical sequence-descriptor tasks, whereas the representation-learning tasks have displaced it into the model itself.

Thirteen hyperparameter-optimization strategies have been deployed across the tasks, namely Grid Search, Randomized Search Cross-Validation (rsCV), Bayesian Optimization, Genetic Algorithms (GA), the Tree-structured Parzen Estimator (TPE), the Optuna framework, nested crossvalidation, threshold optimization through MCC maximization, ensemble model construction, finetuning of pre-trained models, Hyperband with asynchronous successive halving, model-agnostic meta-learning, and automated machine learning.

These range from explicit automated search to deliberately fixed settings. Linear B-cell prediction adopts randomized search cross-validation and grid search, and conformational B-cell prediction relies on fine-tuning of structure-aware language models and ensemble construction. Along the cascade, HLA typing (T1) tunes its convolutional and transformer hyperparameters with Optuna, and antigen processing (T2) uses Hyperband with asynchronous successive halving, where that search doubles as a model comparison and shows that a compact bidirectional long short-term memory network matches the far larger ESM2 and ProtT5-XL language models. MHC class I binding (T3) and MHC class II binding (T4) rely mainly on grid and randomized search with per-allele cross-validation, together with the random-seed and hidden-layer ensembling of the NNAlign lineage that underlies NetMHCIIpan. Immunogenicity prediction (T5) frequently keeps hyperparameters fixed to avoid overfitting on scarce data, so its dominant lever is transfer learning through pretraining and fine-tuning, as in ImmunoStruct and NeoPrecis. TCR-peptide-MHC specificity prediction (T6) optimizes unevenly, because ATM-TCR and ERGO-II use grid search with early stopping, TCR-H uses default settings, and PanPep replaces conventional tuning with model-agnostic meta-learning. Structural TCR-pMHC prediction (T7) adapts pretrained networks, because TCRdock fine-tunes AlphaFold for a small number of epochs and NetTCRstruc trains a cross-validated graph neural network ensemble, whereas TCRmodel2 performs no fine-tuning. Neoantigen identification (T8) tunes its tree-based classifiers in detail, because pTuneos selects random forest over gradient boosting on held-out AUC and the model of Shi and colleagues screens algorithms through automated machine learning. Tumor T-cell antigen classification (T9) implements Bayesian optimization through Optuna with TPE, genetic-algorithm variants, and nested cross-validation. Multi-epitope vaccine design (T10) reports explicit search in every case, with random search in DeepVacPred, grid search in SHASI-ML, and nested cross-validation in Vaxign-ML. The unifying trend is that the modern representation-learning tasks have shifted optimization away from external hyperparameter search and toward transfer learning and the fine-tuning of pretrained models, whereas the classical tasks continue to depend on explicit search.

## 11 Accessibility, reproducibility, and policy-driven barriers in epitope prediction research

The translation of computational epitope prediction advances into vaccine development applications requires accessible and validated tools. Our systematic analysis spans all twelve prediction tasks and reveals significant reproducibility barriers that stem from both deployment practices and institutional publication policies. We organise the analysis in two parts. First, a detailed deployment audit covers the linear B-cell, conformational B-cell, and applied T-cell predictors, where the applied T-cell sub-tasks are neoantigen identification (T8), tumor T-cell antigen classification (T9), and multi-epitope vaccine design (T10), all catalogued in **Table 6**. Second, we summarise code-availability patterns across the upstream T-cell sub-tasks from HLA typing to structural TCR modelling (T1–T7), drawn from **Supplementary Table 4** and **Table 2**. The headline deploymentpractice statistics reported below, namely accessibility rates, dual provision, publisher policy, and temporal decay, are computed on the deeply audited linear B-cell, conformational B-cell, and tumor T-cell antigen studies, for which full journal, publisher, and web-server-longevity information was available. **Table 6** compiles the accessible tools across these tasks, and within the deeply audited set 40 methods offer publicly available code repositories or web server access, and this compilation enables direct assessment of accessibility patterns across the field. The table additionally lists, under separate headings and outside these statistics, the applied neoantigen identification (T8) and multi-epitope vaccine design (T10) tools. Code repository availability varies substantially across prediction tasks. Overall, 70% of studies (40/57) provide at least one accessible resource (code repository or web server), while 30% (17/57) provide neither. Dual accessibility through both code repositories and web servers remains uncommon, observed in only 12.5% (5/40) of accessible methods. Conformational B-cell epitope prediction demonstrates the strongest commitment to open science practices, with 44% of studies (4/9) providing both resources simultaneously. This pattern is exemplified by recent structure-aware methods including GraphBepi (37), SEMA (15, 120), and CBTope2 (39), which integrate protein language model embeddings with graph neural network or inverse folding architectures and maintain active GitHub repositories alongside functional web interfaces. Notably, this observation reveals a counterintuitive but consistent trend: methods employing the most sophisticated architectures like protein language models (ESM-2, ESM-IF1), structure prediction (AlphaFold2), and graph neural networks demonstrate the highest dual accessibility rates. The complexity of three-dimensional structural analysis in conformational epitope prediction appears to necessitate transparent methodological validation, and this requirement likely incentivizes authors to release both executable code and user-facing tools. Linear B-cell epitope prediction exhibits more modest accessibility despite representing the largest study population in **Table 6** with 19 methods. Strikingly, no linear B-cell method (0/19) provides both code and web server access simultaneously, and this is the lowest dual accessibility rate across all prediction categories. Methods are divided between code-only approaches (58%, 11/19) including LBCEBERT (10), NetBCE (108), and EpitopeVec (180), and web server-only tools (42%, 8/19) such as DeepLBCEPred (7), LBCEPred (181), and BepiBlast (117). This binary distribution pattern suggests a divergence in development priorities between computationally-oriented research groups favoring code repositories and application-focused teams prioritizing user-friendly interfaces.

**Table 6:** Summary of B-cell and T-cell epitope prediction methods with available source code or web servers.

Author, year	Representation learning	Classifier	Code link	Web server	Journal	Publisher
Linear B-cell epitope prediction						
Liu et al., 2024 (10)	BERT + AAP + AAC + AAT	XGBoost	https://github.com/Lfang111/LBCE-BERT	–	Sci. Rep.	Nature
Yuan et al., 2023 (13)	BLOSUM62 + BiLSTM + Transformer	MLP	https://github.com/yuanx749/bcell	–	ECML PKDD	Springer
Liu et al. (8), 2023	BERT + AAP + AAC + AAT	XGBoost	https://github.com/liuyf-a/LBCE-XGB	–	Interdiscip. Sci.	Springer
Qi et al., 2023 (7)	Integer Encoding	BiLSTM + CNN + MLP	–	http://www.biolscience.cn/DeepLBCEPred/	Front. Microbiol.	Frontiers
Da et al., 2023 (9)	Graph-based signatures	RF	–	https://biosig.lab.uq.edu.au/epitope1d/	Brief. Bioinform.	Oxford
Attique et al., 2023 (5)	LAFV + RLAFV + FDV + APOV	CNN + MLP	–	http://deeplbcepred.pythonanywhere.com	Comput. Biol. Chem.	Elsevier
Liu et al., 2023 (182)	Smith-Waterman + BLOSUM62	Nadaraya-Watson regression	https://github.com/RanLIUaca/Family-specific-B-cell-epitope-prediction	–	IEEE/ACM TCBB	IEEE
Xu et al., 2022 (108)	Sequence + PCP + Structural features	CNN + BiLSTM + MLP	https://github.com/bsml320/NetBCE	–	Genomics Proteomics Bioinform.	Oxford
Ras-Carmona et al., 2022 (117)	Sequence similarity (BLAST)	Existing B cell epitope Predictors	–	http://imath.med.ucm.es/bepiblast/	Sci. Rep.	Nature
Yin et al., 2022 (112)	ProtVec + QR + AAindex + CTD	XGBoost	https://github.com/Rayin-saber/Epitope-prediction-of-human-adapted-viruses	–	Brief. Bioinform.	Oxford
Alghamdi et al., 2022 (181)	PRIM + RPRIM + Frequency + AAPIM	RF	–	https://lbcepred.pythonanywhere.com/	Brief. Bioinform.	Oxford
Ozger et al., 2022 (110)	AAC + DPC	Fuzzy Ensemble	https://github.com/ZBaOz/Epitope-Identification	–	Appl. Soft Comput.	Elsevier
Shuai et al., 2022 (12)	GCN+Att-BiLSTM	MLP	https://github.com/biolushuai/GCNs-and-Att-BLSTM-for-BCEs-prediction	–	Front. immunolo.	Frontiers
Ras-Carmona et al., 2021 (183)	AAC + DPC + Combined composition	SVM	–	http://imath.med.ucm.es/bceps/	Cells	MDPI
Ashford et al., 2021 (109)	AAC + DPC + Conjoint triad	RF	https://github.com/fcampelo/OrgSpec-paper	–	Bioinformatics	Oxford
Bahai et al., 2021 (180)	AAC + DPC + AAP + ProtVec	SVM	https://github.com/hzi-bifo/epitope-prediction-paper	–	Bioinformatics	Oxford
Collatz et al., 2021 (34)	ELMo	LSTM	https://github.com/mcollatz/EpiDope	–	Bioinformatics	Oxford
Hasan et al., 2020 (184)	AIP + AFC + PSSM + PKAF	RF + LR	–	http://kurata14.bio.kyutech.ac.jp/iLBE/	Genomics Proteomics Bioinform.	Oxford
Liu et al., 2020 (107)	DPC	Ensemble DNN	–	https://bio.tools/dlbepitope	BioData Min.	Springer
Conformational B-cell epitope prediction						
Pandey et al., 2025 (39)	Composition + Binary profiles	SVM + RF	https://github.com/raghavagps/cbtope2	https://webs.iiitd.edu.in/raghava/cbtope2/	arXiv preprint	arXiv
Ivanisenko et al., 2024 (15)	ESM-2 + SaProt	MLP	https://github.com/AIRI-Institute/SEMAi	https://sema.airi.net/	Nucleic Acids Res.	Oxford
Choi et al., 2023 (6)	ESM-2 + PCP + GNN	MLP + kNN	https://github.com/sj584/EpiCluster	–	bioRxiv	CSHL
Zeng et al., 2023 (37)	AlphaFold2 + ESM-2 + EGNN	MLP	https://github.com/biomed-AI/GraphBepi	http://bio-web1.nscc-gz.cn/app/graphbepi	Bioinformatics	Oxford
Shashkova et al., 2022 (120)	ESM-1v + ESM-IF1	MLP	https://github.com/AIRI-Institute/SEMAi	https://sema.airi.net/	Front. Immunol.	Frontiers
Da Silva et al., 2022 (3)	Graph-based signatures	AdaBoost	–	https://biosig.lab.uq.edu.au/epitope3d/	Brief. Bioinform.	Oxford
Lu et al. (12), 2022	GCN + Att-BiLSTM	MLP	https://github.com/biolushuai/GCNs-and-Att-BLSTM-for-BCEs-prediction	–	Front. Immunol.	Frontiers
Hou et al., 2021 (185)	Sequence-based interface features	RF	–	https://www.ibi.vu.nl/programs/serendipwww/	Bioinformatics	Oxford
Solihah et al., 2020 (121)	ASA + RSA + PI + CN + HSE	Decision Tree	https://github.com/BSolihah/conformational-epitope-predictor	–	PeerJ Comput. Sci.	PeerJ
Linear & conformational B-cell epitope prediction						
Kumar et al., 2024 (2)	BLAST + DPC	RF	–	https://webs.iiitd.edu.in/raghava/clbtope	Comput. Biol. Med.	Elsevier
Høie et al. (36), 2024	ESM-IF1 + AlphaFold	XGBoost	–	https://services.healthtech.dtu.dk/services/DiscoTope-3.0/	Front. Immunol.	Frontiers
Israeli et al., 2024 (173)	Kidera factors + ESM-2/ESM-IF1	BiLSTM + MLP	https://github.com/louzounlab/epitope_b_cells_predictor	https://caliber.math.biu.ac.il/	Brief. Bioinform.	Oxford
Clifford et al., 2022 (38)	ESM-2 + BLOSUM62	MLP	–	https://services.healthtech.dtu.dk/services/BepiPred-3.0/	Protein Sci.	Wiley
Neoantigen identification (T8)						
Cai et al., 2025 (88)	TF-IDF + BioBERT embeddings	FCNN (BioBERT fusion)	–	http://www.cnneodb.cn/	medRxiv	CSHL
Zhao et al., 2024 (86)	aKDE + isotonic regression	Logistic Regression	https://github.com/XuegongLab/neoguider	–	Research Square	Research Square
Zhou et al., 2019 (85)	BLOSUM62 + hydrophobicity + %rank	Random Forest	https://github.com/bm2-lab/pTuneos	–	Genome Med.	Springer
Tumor T-cell antigen prediction (T9)						
Hassan et al., 2024 (139)	10 descriptors (ACC, PAAC, APAAC, DPC, CTD, GPSD, etc.)	Ensemble (RF+XGB+ LGBM+CBC+ETC)	https://github.com/MirTanveer/TTCA-IF	–	BioSystems	Elsevier
Yu et al., 2024 (97)	57 descriptors (CKSAAP, DDE, DPC, CTriad, CTD, physicochemical, etc.)	GBC + ET	https://github.com/YnnJ456/ENCAP	–	PLoS ONE	PLoS
Charoenkwan et al., 2023 (95)	DPS + GDPS	RF	–	https://pmlabstack.pythonanywhere.com/PSRTTCA	Comput. Biol. Med.	Elsevier
Charoenkwan et al., 2023 (96)	12 descriptors (AAC, AAI, APAAC, CTD, DPC, PCP, PAAC, etc.)	ET	–	https://pmlabstack.pythonanywhere.com/StackTTCA	BMC Bioinform.	Springer
Zou et al., 2022 (93)	AC + CC + PCC + PCC(HON-1/2)	SVM	https://figshare.com/articles/online_resource/iTTCA-MFF/17636120	–	Immunogenetics	Springer
Herrera-Bravo et al., 2021 (136)	AAIndex properties	QDA	https://github.com/jfbldevs/AIDApy	–	Comput. Biol. Chem.	Elsevier
Jiao et al., 2021 (92)	GPSD+GAAPC+PAAC	RF	–	http://lab.malab.cn/~acy/iTTCA/	J. trans. med.	Springer
Charoenkwan et al., 2020 (137)	PseAAC + CTDD	RF	https://github.com/Shoombuatong/Dataset-Code	–	Anal. Biochem.	Elsevier
Multi-epitope vaccine design (T10)						
Yang et al. (100), 2021	Z-descriptors + auto-cross-covariance	DNN (CNN + dense)	https://github.com/zikunyang/DCVST	–	Sci. Rep.	Nature
Ong et al., 2020 (101)	Reverse-vaccinology features	XGBoost	–	http://www.violinet.org/vaxign2/	Front. Immunol.	Frontiers

Studies are organized by task type (Linear B-cell, Conformational B-cell, Linear & Conformational B-cell, T-cell) and from recent to oldest within each category. The accessibility statistics reported in the running text are computed on the deeply audited linear B-cell, conformational B-cell, and tumor T-cell antigen (TTCA, T9) studies. The separately headed Neoantigen Identification (T8) and Multi-epitope Vaccine Design (T10) blocks list representative applied T-cell tools for completeness, and they are not included in those statistics. The broader code-availability landscape across the upstream T-cell sub-tasks (T1–T7) is given in **Supplementary Table 4** and **Table 2**.

T-cell epitope prediction (tumor T-cell antigen studies, T9) exhibits notable accessibility limitations among all prediction domains (92–97, 134, 136–140). Our analysis reveals that 33% (4/12) of these T-cell prediction studies provide neither accessible code nor a functional web server, and this is a higher inaccessibility rate than in other epitope prediction categories. Among the 8 accessible T-cell methods in **Table 6**, no predictor provides both code repository and web server access simultaneously, which yields a dual accessibility rate of 0% (0/8). The accessible methods are divided between code-only approaches (62.5%, 5/8) including ENCAP (97), iTTCA-IF (139), iTTCA-MFF (93), TAP (136), and iTTCA-Hybrid (137), and web server-only tools (37.5%, 3/8) such as PSRTTCA (95), StackTTCA (96), and iTTCA-RF (92). This fragmented accessibility pattern contrasts sharply with conformational B-cell methods, where dual resource provision has become increasingly standard. The T-cell epitope prediction domain also exhibits a striking methodological conservatism that distinguishes it from B-cell prediction approaches. While conformational B-cell methods have rapidly adopted protein language models (ESM-2, ESM-IF1, SaProt) and structure prediction tools (AlphaFold2), all 12 audited tumor T-cell antigen predictors published between 2019 and 2024 rely exclusively on traditional hand-crafted sequence descriptors including amino acid composition (AAC), dipeptide composition (DPC), pseudo amino acid composition (PseAAC), and physicochemical property-based encodings from AAIndex (95–97, 137, 139). This representational gap suggests that tumor T-cell antigen prediction may be poised for substantial methodological advancement through protein language model integration. Furthermore, Random Forest remains the dominant classifier in tumor T-cell antigen prediction, employed in approximately half of studies (92, 95, 137), whereas conformational B-cell methods have transitioned predominantly to neural network architectures including multi-layer perceptrons. This classifier conservatism, combined with the absence of learned representations, indicates that tumor T-cell antigen prediction lags approximately 3–4 years behind the methodological frontier established by B-cell prediction methods.

Beyond the tumor T-cell antigen sub-task, the applied and upstream T-cell sub-tasks present a markedly more open accessibility profile. Among the applied sub-tasks, the neoantigen identification methods are predominantly open source, because the machine-learning and deep-learning predictors pTuneos (85), NeoGuider (86), and CNNeoPP (88), together with the widely used pVACtools (133) and NeoPredPipe (89) annotation pipelines, all provide maintained public repositories or web servers. The multi-epitope vaccine-design tools combine open code in DeepVacPred (100) with the Vaxign-ML web server (101), although SHASI-ML releases its code only on request (102). The upstream sub-tasks (T1–T7) are more open still. The MHC binding predictors NetMHCpan-4.1, MHCflurry 2.0, and TransPHLA, the TCR specificity predictors NetTCR, ERGO-II, TULIP, and pMTnet, the structural modelling tools TCRdock and TCRmodel2, and the HLA typing tool CookHLA are released as open-source repositories or maintained web servers, as catalogued per sub-task in **Supplementary Table 4** and **Table 2**. The reproducibility deficit documented above is therefore concentrated in the tumor T-cell antigen sub-task rather than in T-cell prediction as a whole.

Temporal analysis further indicates that accessibility degrades with publication age: studies published prior to 2021 are disproportionately represented among web-only tools and non-functional servers, whereas recent studies, particularly among the conformational B-cell predictors, more frequently provide maintained code repositories. This temporal decay disproportionately affects web server-only deployments, as evidenced by the observed instability of cloud-based hosting solutions including PythonAnywhere free-tier deployments and Heroku-hosted applications. The vulnerability of these platforms for long-term scientific resource maintenance underscores the importance of code repository provision as a complement to web server deployment.

Publication venue selection demonstrates measurable impact on research accessibility that extends beyond individual researcher choices. Three major publishers account for approximately two-thirds of the 57 audited predictor studies (38/57, 67%), and their divergent data sharing policies create systematic differences in code availability. Oxford University Press venues mandate code and data deposition for computational studies, and these journals achieve higher code availability across their epitope prediction publications. This policy effect is evident in the concentration of well-documented methods in journals such as Bioinformatics and Briefings in Bioinformatics, where tools including EpiDope (34), Epitope1D (9), and organism-specific predictors (109) provide comprehensive GitHub repositories. In contrast, Elsevier journals maintain optional **Supplementary Material** policies, and these venues show comparatively lower code availability. The concentration of T-cell epitope prediction studies in Elsevier venues such as Computers in Biology and Medicine (95), Computational Biology and Chemistry (136), Analytical Biochemistry (137), and BioSystems (139) coincides with this domain’s mixed code sharing practices. GitHub serves as the dominant platform for code distribution across all prediction domains, and this reflects its integration with modern collaborative development workflows and version control capabilities. Alternative platforms including Figshare provide specialized roles for dataset and code distribution, as exemplified by iTTCA-MFF (93). Web server hosting shows greater diversity, with platforms ranging from institutional deployments at academic servers to cloud-based solutions such as PythonAnywhere that host multiple T-cell prediction tools (95, 96).

The absence of standardized deployment practices creates additional barriers beyond simple availability. Web servers frequently become non-functional within years of publication, and code repositories may lack documentation, dependency specifications, or example datasets necessary for independent validation. Methods that provide both resources offer the most robust accessibility, as code repositories enable methodological transparency while web servers facilitate broader community adoption without requiring local installation. Collectively, these findings establish a clear relationship between publisher policy, code availability, reproducibility, and ultimately translational readiness. The field would benefit from mandatory code and data deposition policies, and such requirements could increase overall accessibility rates. Additionally, establishing minimum validation requirements that include independent test set evaluation, comparison with at least three existing methods, and public code deposition would further harmonize quality standards while preserving venue diversity and accelerating clinical translation of epitope prediction methods.

## 12 Discussion

The computational identification of B-cell and T-cell epitopes represents a critical frontier in immunoinformatics with far-reaching implications for vaccine development, immunotherapy design, and precision medicine applications (32, 33). Our comprehensive analysis of 155 studies across the unified predictive pipeline reveals challenges that transcend individual epitope types and collectively constrain the field’s advancement toward clinically relevant prediction tools. At the foundational data acquisition level, the field confronts a critical paradox where the scarcity of high-quality experimental data simultaneously limits model development and validation efforts. The reliance on costly experimental methods including X-ray crystallography, NMR spectroscopy, and traditional epitope mapping techniques creates fundamental bottlenecks in data generation (28, 30). Our analysis reveals that 29% of epitope databases remain significantly underutilized, with databases such as Bcipep showing declining usage patterns and critical T-cell resources including SYFPEITHI, MHCBN 4.0, and SysteMHC Atlas being systematically overlooked despite containing valuable immunological information. This underutilization reflects deeper issues in database accessibility and classification that prevent researchers from effectively navigating the complex ecosystem of available data sources. Across the full T-cell taxonomy, this picture sharpens further. The canonical receptor and genotyping resources that anchor modern T-cell prediction, namely VDJdb and McPAS-TCR for TCR–peptide-MHC specificity, IPD-IMGT/HLA for HLA typing, and STCRDab for structural modelling, are individually rich yet remain siloed by sub-task, and the immunopeptidomics corpora that underpin MHC binding (the NetMHCpan and NetMHCIIpan eluted-ligand data and SysteMHC Atlas) are concentrated in a few laboratories. The consequence is not a shortage of data so much as fragmentation across resources that are rarely integrated within a single predictive pipeline.

Dataset quality issues compound accessibility challenges through pervasive problems with ambiguous epitope definitions, contradictory experimental results, and inadequate metadata documentation. The benchmark datasets compiled from the Immune Epitope Database demonstrate significant metadata gaps, with only 13 of 57 linear B-cell epitope datasets containing essential antigenic source information. The absence of complete protein sequence information for antigens prevents sophisticated feature engineering approaches and limits biological context available for model training (11). The persistent challenge of class imbalance pervades all epitope prediction domains, and the systematic reliance on non-experimentally validated sequences from general protein databases introduces potential label noise that can mislead machine learning algorithms toward learning spurious patterns rather than genuine biological signatures. The representation learning landscape demonstrates both progress and persistent gaps. BERT-based protein language models show superior performance when rigorously evaluated (8, 10), yet their adoption remains limited by computational resource requirements that create accessibility barriers across research groups. The challenge of epitope length variability constrains representation learning effectiveness, as most encoding schemes assume fixed-length inputs while biological epitopes demonstrate significant natural variation. Current approaches to handle variable-length sequences through padding or truncation introduce artificial patterns that may mislead learning algorithms. For conformational B-cell epitope prediction, the clustering property of epitopes has been largely overlooked in existing representation schemes despite its demonstrated biological significance (6), and the absence of post-translational modifications in most representations constitutes a significant limitation given their substantial influence on protein surface properties.

Classification approaches across epitope prediction domains demonstrate diversity in algorithmic choices but suffer from consistent generalizability challenges. Random Forest and Support Vector Machine algorithms maintain popularity due to their robust performance characteristics and relative interpretability (31), yet systematic evaluation across multiple independent datasets reveals concerning inconsistencies that suggest overfitting to specific training distributions. The computational costs associated with advanced approaches create accessibility barriers that limit adoption and potentially create disparities in research capability. Graph Convolutional Networks show promise for conformational epitope prediction by capturing spatial relationships among amino acid residues (12), yet their application remains constrained by limited structural data availability and computational intensity. Evaluation practices reveal systematic deficiencies that compromise the reliability of reported performance metrics. The lack of consistent benchmarking standards creates fundamental challenges for comparative evaluation, as different studies employ varying datasets, evaluation protocols, and performance metrics. The observation that performance on specific benchmarks does not translate to performance on alternative datasets (10, 123) indicates that the field may be collectively optimizing for benchmark-specific patterns rather than generalizable biological signals. Cross-validation approaches often fail to account for sequence similarity between training and testing examples, and the limited adoption of temporal validation approaches represents a missed opportunity for assessing model stability as biological knowledge evolves.

Across the ten-sub-task T-cell landscape (Section 5), deep learning and protein language models are the dominant paradigm in MHC class I and class II binding, immunogenicity, and TCR– peptide-MHC specificity. Deep learning accounts for 64 of 103 T-cell models, and ESM-, BERT-, and transformer-based encoders are used extensively in MHC binding (NetMHCpan-4.1, BigMHC, TransPHLA) and most heavily of all in TCR specificity (TCR-ESM, TULIP, STAPLER). The principal T-cell challenges are specific and recurring failure modes. First, generalisation to unseen epitopes collapses in TCR-specificity prediction, a pattern that the IMMREP22 benchmark and NetTCR-2.1 confirm and that is compounded by undefined negative sampling and the over-representation of HLA-A*02:01. Second, immunogenicity prediction (T5) plateaus at AUC values of roughly 0.6–0.8 because negatives are ill-defined and clinical correlation is rarely tested, which directly limits the neoantigen pipelines (T8) that depend on it. Third, MHC class II prediction lags class I, with DQ and DP alleles severely under-represented relative to DR, and structural TCR modelling (T7) is constrained by only a few hundred experimental complexes and heavy compute cost. Fourth, HLA typing and imputation (T1), the genotyping foundation of every downstream task, under-represents non-European populations and resolves class II alleles poorly, which is an equity concern as well as a technical one. Finally, the tumor T-cell antigen sub-task (T9) has only recently seen its first deeplearning entrants, iTTCA-DNN (98) and LYnet (99), and no dedicated predictor there yet adopts a protein language model, so it remains the clearest within-field opportunity for the protein language models that have already transformed the upstream sub-tasks.

Deployment infrastructure presents substantial barriers to practical application. Among methods included in **Table 6**, 42.1% of linear B-cell epitope predictors (8/19), 66.7% of conformational B-cell epitope predictors (6/9), and 37.5% of T-cell epitope predictors (3/8) provide accessible web server interfaces. Notably, dual accessibility through both code repositories and web servers remains rare: 0% for linear B-cell, 44.4% for conformational B-cell, and 0% for T-cell methods. These figures pertain to the tumor T-cell antigen predictors (T9). Across the broader T-cell sub-tasks the picture is considerably more favourable, because the modern MHC-binding, immunogenicity, TCR-specificity, neoantigen, and structural tools are released predominantly as maintained open-source repositories and web services (**Supplementary Table 4**; **Table 2**), with NetMHCpan, MHCflurry, NetTCR, ERGO-II, TULIP, TCRdock, pVACtools, and CookHLA among the openly available examples. The reproducibility deficit is therefore concentrated in the TTCA sub-task rather than in T-cell prediction as a whole. The limited availability of source code repositories creates significant barriers to reproducibility verification and collaborative methodological advancement. API availability remains extremely limited, and this absence prevents integration with automated analysis pipelines and workflow management systems. The sustainability of deployed tools represents an ongoing concern, as many web servers suffer from inconsistent maintenance that can result in service interruptions or degraded performance over time. The interconnected nature of these challenges creates compounding effects that extend beyond individual limitations. Inadequate datasets lead to overfitted models, fragmented evaluation prevents identification of genuine advances, and limited deployment constrains community validation. Breaking this cycle requires coordinated intervention across multiple pipeline stages rather than isolated improvements to individual components. The observation from Villanueva-Flores et al. (32) that models sometimes inadvertently overfit public benchmarks underscores the need for blind, prospective evaluations and experimental validation to truly test model generality.

## 13 Conclusion and future directions

Our comprehensive analysis of 155 studies spanning 48 databases, 144 benchmark datasets, 272 representation learning approaches, and 148 classification methodologies reveals a field characterized by remarkable methodological diversity yet constrained by interconnected limitations that collectively impede clinical translation. The systematic examination demonstrates both significant progress achieved and substantial challenges that persist across linear B-cell, conformational Bcell, and T-cell epitope prediction domains. The path toward clinically relevant epitope prediction tools requires strategic prioritization of several research directions. First, comprehensive database integration efforts must establish unified classification frameworks that enable effective navigation of available resources while addressing quality issues through rigorous curation protocols and metadata enhancement initiatives. The development of standardized negative control definitions based on rigorous experimental validation represents a fundamental conceptual priority, as the biological definition of what constitutes a definitive non-epitope remains unclear and context-dependent across immunological applications.

Second, advanced representation learning approaches offer substantial potential that remains incompletely realized. Protein language models and transformer-based architectures require systematic exploration and optimization for epitope-specific applications, and the integration of multi-scale feature representations combining sequence, structural, and evolutionary information through sophisticated fusion architectures represents a promising direction. Transfer learning and domain adaptation approaches should be extensively explored to address limited data availability constraints that particularly affect T-cell epitope prediction and conformational B-cell epitope identification. Within the T-cell taxonomy, the clearest near-term gains lie in extending the protein language models that already dominate MHC binding and TCR specificity to the sub-tasks that still rely on hand-crafted descriptors, most notably tumor T-cell antigen classification (T9), and in maturing the structure-aware and graph-based approaches that remain nascent for MHC class II and structural TCR modelling. Third, evaluation standardization demands transformation toward community-maintained benchmark collections with rigorous curation standards and temporal validation frameworks. Cross-task evaluation approaches that assess model generalization across different epitope types represent an underexplored opportunity for identifying truly robust predictive frameworks. The establishment of standardized reporting guidelines would enhance reproducibility and enable more effective comparative analysis of methodological advances. For Tcell prediction specifically, the single most important evaluation reform is the universal adoption of unseen-epitope and unseen-TCR splits, because the steep performance drop documented by the IMMREP22 community benchmark shows that conventional random splits substantially overstate real-world TCR-specificity performance. Immunogenicity prediction similarly requires standardised, experimentally grounded negative sets to escape its current AUC plateau. Equitable HLA-typing imputation across non-European populations is a further priority, since this genotyping step underpins every downstream T-cell prediction and currently propagates population bias into the entire pipeline.

Fourth, the integration of multi-omics data offers transformative potential for developing holistic models of immune recognition. Immunopeptidomics data from mass spectrometry experiments provides direct evidence of naturally processed and presented epitopes that could substantially enhance training data quality and biological relevance. The incorporation of host genetic variation, pathogen evolution patterns, and immune system status information could enable personalized epitope prediction approaches. Fifth, deployment infrastructure requires comprehensive modernization through standardized APIs, workflow integration capabilities, and cloud-based architectures that address computational resource limitations. Community-supported infrastructure for hosting and maintaining prediction services could address sustainability concerns while ensuring consistent availability across tools. The ultimate goal extends beyond algorithmic sophistication toward reliable, interpretable, and accessible tools that meaningfully support vaccine design, immunotherapy development, and diagnostic applications in real-world settings. The foundation for this transformation exists within the current body of research, yet realizing its potential requires sustained collaboration between computational and experimental communities working toward shared goals of advancing human health through improved understanding of immune recognition processes.

## Footnotes

1 https://www.biorender.com/.

## Supplementary material

Four supplementary reference tables accompany this review. **Supplementary Table 1** linear B-cell epitope prediction datasets and **Supplementary Table 2** comprehensive performance analysis of linear B-cell epitope predictors present detailed material that complements the main text. Two further tables support the ten-sub-task T-cell survey: **Supplementary Table 3** key benchmark datasets and corpora across the ten T-cell sub-tasks T1–T10, with approximate scale, MHC class, and accessibility and **Supplementary Table 4** representative per-study methods across the T-cell sub-tasks, with their representation learning, classifier, headline performance, and code availability. All four are cited at the relevant points in the main text.
