# Supplementary material for: Computational identification of B- and T-cell epitopes: a unified task taxonomy and review of databases, datasets, predictive pipelines, and gaps
Source: Front Immunol. 2026 Sep 17;17:1873135. doi: 10.3389/fimmu.2026.1873135 (PMC13628299; doi:10.3389/fimmu.2026.1873135)
Supplement: Supplementary file 1 [file DataSheet1.pdf]

Supplementary Materials

Computational Identification of B- and T-cell Epitopes: A Unified Task Taxonomy and Review of Databases, Datasets, Predictive Pipelines, and Gaps

Overview

This supplementary document contains the large reference tables that accompany the main manuscript, together with the detailed per-sub-task tables that support the ten-sub-task T-cell survey. Among the ten T-cell sub-tasks, T1–T7 apply to the epitopes of any antigen. Neoantigen identification (T8) and tumor T-cell antigen (TTCA) classification (T9) are specific to the oncology setting, whereas multi-epitope vaccine design (T10) applies across infectious-disease and cancer targets. **Supplementary Table S1** lists the linear B-cell epitope prediction datasets together with their composition, species/notes, experimental settings, source databases, and accessibility status. **Supplementary Table S2** provides the comprehensive performance analysis of linear B-cell epitope predictors. **Supplementary Table S3** catalogues the key benchmark datasets and corpora used across the ten T-cell prediction sub-tasks (T1–T10), with scale, MHC class, and accessibility. **Supplementary Table S4** summarises representative T-cell methods per sub-task with their representation learning, classifier, headline performance, and code availability. All tables are referred to from the main manuscript. Performance values in Supplementary Tables S2 and S4 are reported as originally published by each study. They derive from heterogeneous datasets, splits, class-balance ratios, and evaluation protocols and are *not* directly comparable across studies or sub-tasks.

Table S1: Linear B-Cell Epitope Prediction Datasets.

No.	Dataset Name	Epitopes	Non-Epitopes	Total	Species/Notes	Experimental Settings	Database	Public/In-House	Accessible
1	ABCPred [1]	700	700	1,400	-	5-fold Cross Validation, Independent Test Set	IEDB, BciPep	Public	Yes
2	ABCPred-1 [2]	700	700	1,400	-	Train=80%, Test=20%, 10-fold CV	IEDB	Public	Yes
3	ABCPred-2 [2]	187	200	387	-	Train=80%, Test=20%, 10-fold CV	IEDB	Public	Yes
4	ABCPred16 (v1) [3]	109	201	310	-	Train=80%, Test=20%, 10-fold CV	IEDB	Public	Yes
5	ABCPred16 (v2) [4]	700	700	1,400	-	5-fold cross-validation	IEDB	Public	Yes
6	ABCPred16 (v3) [5]	107	196	303	-	Not mentioned	IEDB, NCBI	Public	Yes
7	Amaya et al. dataset [6]	2,117	4,769	6,886	-	Train=80%, Test=20%, 10-fold CV	HLA Eplet Registry	In-House	No
8	Angaitkar et al. dataset [3]	-	-	48,000	-	Train=80%, Test=20%, 10-fold CV	IEDB	Public	Yes
9	Bahai et al. Viral Dataset [4]	4,432	8,460	12,892	Viral dataset	5-fold cross-validation	IEDB	Public	Yes
10	BCETD555 (Ras-Carmona et al.) [7, 8]	555	555	1,110	-	Train=80%, Test=20%, 10-fold CV	IEDB; abY-bank/AbDb	Public	Yes
11	BCPred [2, 9, 4]	701	701	1,402	-	Train=80%, Test=20%, 10-fold CV; 5-fold cross-validation	IEDB	Public	Yes
12	BCPreds [1]	701	701	1,402	-	5-fold Cross Validation, Independent Test Set	IEDB, BciPep	Public	Yes
13	BepiPred-2.0 [10, 11, 12]	11,834	18,722	30,556	-	5-fold Cross Validation; Train=70%, Test=30%, 10-fold CV, LOOCV	IEDB	Public	Yes
14	BepiPred-2.0, EpiDope [13]	11,834	18,722	30,556	-	Train=70%, Test=30%, 10-fold CV, LOOCV	IEDB	Public	Yes
15	Blind387 [1, 4]	187	200	387	-	5-fold Cross Validation, Independent Test Set	IEDB, BciPep	Public	Yes
16	Carmona et al. Datasets [14]	62,730	-	62,730	-	Not specified	IEDB	Public	Yes
17	Chen [1, 2, 3]	872	872	1,744	-	Train=80%, Test=20%, 10-fold CV; 5-fold Cross Validation, Independent Test Set	IEDB, BciPep	Public	Yes
18	Chen et al. Dataset [4, 15]	872	872	1,744	-	5-fold cross-validation; Not specified	IEDB	Public	Yes
19	D100 [16]	15,000	15,000	30,000	-	Train=80%, Test=20%, 5-fold CV	NA	Public	Yes
20	D40 [16]	2,000	2,000	4,000	-	Train=80%, Test=20%, 5-fold CV	NA	Public	Yes

Continued on next page

Table S1 – *Continued from previous page*

No.	Dataset Name	Epitopes	Non-Epitopes	Total	Species/Notes	Experimental Settings	Database	Public/In-House	Accessible
21	Dengue virus dataset [17]	5,008	2,012	7,020	Dengue virus	Not mentioned	IEDB	In-House	No
22	EpiDope [13]	-	-	-	-	5-fold CV	IEDB	Public	Yes
23	epitope1D Core [2]	20,638	103,281	123,919	Multi-taxonomy (20 groups)	Train=80%, Test=20%, 10-fold CV	IEDB	Public	Yes
24	epitope1D IND [2]	5,264	25,716	30,980	Multi-taxonomy (20 groups)	Train=80%, Test=20%, 10-fold CV	IEDB	Public	Yes
25	Epstein-Barr [18]	3,727	2,675	6,402	Epstein-Barr virus	Train=75%, Test=25%	IEDB, UniProt, NCBI	Public	Yes
26	Hep. C virus [18]	2,845	2,758	5,603	Hepatitis C virus	Train=75%, Test=25%	IEDB, UniProt, NCBI	Public	Yes
27	iBCE-EL Core [1, 19, 4, 20]	4,440	5,485	9,925	-	Independent Test Set; Train=80%, Test=20%, 5-fold CV	IEDB, BciPep	Public	Yes
28	iBCE-EL IND [1, 19, 4, 20]	1,110	1,408	2,518	-	Independent Test Set; Train=80%, Test=20%, 5-fold CV	IEDB, BciPep	Public	Yes
29	IDED1246 dataset [7, 8]	1,246	1,246	2,492	-	Train=80%, Test=20%, 10-fold CV	IEDB; abY-bank/AbDb	Public	Yes
30	IEDB16 [5]	-	-	-	-	Not mentioned	IEDB, NCBI	Public	Yes
31	IEDB18 [5]	-	-	-	-	Not mentioned	IEDB, NCBI	Public	Yes
32	IEDB20 [5]	-	-	-	-	Not mentioned	IEDB, NCBI	Public	Yes
33	IEDB22 [5]	-	-	-	-	Not mentioned	IEDB, NCBI	Public	Yes
34	IEDB31 [5]	-	-	-	-	Not mentioned	IEDB, NCBI	Public	Yes
35	IEDB38 [5]	22,012	201,563	223,575	-	Not mentioned	IEDB, NCBI	Public	Yes
36	ILED2195 dataset [7, 8]	2,195	2,195	4,390	-	Train=80%, Test=20%, 10-fold CV	IEDB; abY-bank/AbDb	Public	Yes
37	IND: BepiPred-2.1 [21]	11,834	18,722	30,556	-	5-fold Cross Validation	IEDB	In-House	No
38	Kozlova et al. dataset [22]	40	-	40	Murine antibodies	10-fold Cross Validation	IEDB	Public	Yes
39	Kumar et al. Dataset [23]	3,875	3,996	7,871	-	5-fold cross-validation	IEDB	Public	Yes
40	La et al. dataset [21]	9,968	10,766	20,734	-	5-fold Cross Validation	IEDB	In-House	No
41	LBtope (15K) [1, 2, 4]	7,824	7,853	15,677	-	5-fold CV, Independent Test Train=80%, Test=20%, 10-fold CV	IEDB, BciPep	Public	Yes
42	LBtope (45K) [24]	21,999	23,321	45,320	-	Train=80%, Test=20%, 10-fold CV	IEDB	Public	Yes
43	LBtope_Confirm [16]	1,042	1,795	2,837	-	Train=80%, Test=20%, 5-fold CV	NA	Public	Yes
44	Lbtope_Fixed [5]	8,661	16,492	25,153	-	Not mentioned	IEDB, NCBI	Public	Yes
45	Lbtope_Fixed non_redundant [25]	7,000	7,000	14,000	-	5-fold Cross Validation	NA	In-House	No

Continued on next page

Table S1 – *Continued from previous page*

No.	Dataset Name	Epitopes	Non-Epitopes	Total	Species/Notes	Experimental Settings	Database	Public/In-House	Accessible
46	LBTope-Fixed [3]	7,825	7,852	15,677	-	Train=80%, Test=20%, 10-fold CV	IEDB	Public	Yes
47	Lim et al. dataset [26]	18,404	203,088	301,915	Multi-species (Bacteria, Fungi, Viruses, Multicellular, Unicellular)	5-fold Cross Validation	IEDB	In-House	No
48	NetBCE [27, 28]	27,095	97,784	124,879	-	Train-test split; Train=90%, Test=10%, 5-fold CV	IEDB; NCBI, UniProt	Public	Yes
49	O. volvulus [18]	5,075	5,300	10,375	O. volvulus (Nematoda)	Train=75%, Test=25%	IEDB, UniProt, NCBI	Public	Yes
50	SARS-CoV epitopes [29]	140	380	520	SARS-CoV	Repeated 6 times	IEDB, UniProt	Public	Yes
51	SARS-CoV-2 peptides [29]	20,312	-	20,312	SARS-CoV-2	Repeated 6 times	IEDB, UniProt	Public	Yes
52	Tung et al. Dataset [30]	52,159	127,010	179,169	-	80% Train, 20% Test	IEDB	In-House	No
53	Velpula et al. Dataset [31]	14,362	-	14,387	SARS-CoV-2, 757 proteins	LOOCV	IEDB, UniProt	Public	Yes
54	Yang et al. Dataset [15]	872	872	1,744	-	Not specified	IEDB	Public	Yes
55	Yin et al. dataset [32]	4,975	4,956	9,931	Human-adapted viruses (17 species)	Train=90%, Test=10%, 5-fold CV	IEDB	Public	Yes
56	Yuan et al. dataset [27]	64,940	64,019	128,959	-	Train-test split	IEDB	Public	Yes
57	Zika virus dataset [17]	1,261	480	1,741	Zika virus	Not mentioned	IEDB	In-House	No

Table S2: A Comprehensive Performance Analysis of Linear B cell Epitope Predictors. **Note:** Performance values in the "Performance Results" column are reported as originally published by each study. They derive from heterogeneous datasets, data splits, class-balance ratios, and evaluation protocols; they are *not* directly comparable across studies. Readers should consult the "Dataset & Evaluation" column for context before drawing cross-study inferences.

**Author, Year**	**Method Details**	**Dataset & Evaluation**	**Performance Results**	**Comparisons**
**Liu et al., 2024 [1]**	**Rep. Learning:** BERT + AAP + AAC + AAT<br>**Classifier:** XGBoost	**Datasets:** 1. ABCPred, 2. Chen, 3. BCPreds, 4. Blind387, 5. LBtope, 6. iBCE-EL (Core, IND)<br>**Type:** Public<br>**Validation:** 5-fold CV, Independent Test	**1. ABCPred:** LBCE-BERT (BCPreds): Precision=0.873, Sn=0.886, F1=0.879, MCC=0.757, Acc=0.879, AUC=0.934; LBCE-BERT (LBtope): Precision=0.637, Sn=0.454, F1=0.530, MCC=0.204, Acc=0.598, AUC=0.617; LBCE-BERT (iECB-EL): Precision=0.619, Sn=0.520, F1=0.565, MCC=0.203, Acc=0.600, AUC=0.625<br>**2. Chen:** LBCE-BERT (BCPreds): Precision=0.911, Sn=0.977, F1=0.943, MCC=0.884, Acc=0.941, AUC=0.990; LBCE-BERT (LBtope): Precision=0.650, Sn=0.460, F1=0.539, MCC=0.222, Acc=0.606, AUC=0.639; LBCE-BERT (iECB-EL): Precision=0.598, Sn=0.525, F1=0.559, MCC=0.173, Acc=0.586, AUC=0.616<br>**3. BCPreds:** LBCE-BERT (BCPreds): Precision=0.872, Sn=0.820, F1=0.844, MCC=0.701, Acc=0.850, AUC=0.924; LBCE-BERT (LBtope): Precision=0.664, Sn=0.482, F1=0.559, MCC=0.248, Acc=0.619, AUC=0.641; LBCE-BERT (iECB-EL): Precision=0.619, Sn=0.534, F1=0.573, MCC=0.207, Acc=0.603, AUC=0.643<br>**4. Blind387:** LBCE-BERT (BCPreds): Precision=0.759, Sn=0.572, F1=0.652, MCC=0.418, Acc=0.705, AUC=0.757; LBCE-BERT (LBtope): Precision=0.618, Sn=0.519, F1=0.564, MCC=0.223, Acc=0.612, AUC=0.656; LBCE-BERT (iECB-EL): Precision=0.583, Sn=0.524, F1=0.552, MCC=0.175, Acc=0.589, AUC=0.626<br>**5. LBtope:** LBCE-BERT (BCPreds): Precision=0.555, Sn=0.208, F1=0.208, MCC=0.054, Acc=0.522, AUC=0.547; LBCE-BERT (LBtope): Precision=0.665, Sn=0.686, F1=0.675, MCC=0.342, Acc=0.671, AUC=0.733; LBCE-BERT (iECB-EL): Precision=0.589, Sn=0.624, F1=0.624, MCC=0.624, Acc=0.595, AUC=0.633<br>**6. iBCE-EL:** LBCE-BERT (BCPreds): Precision=0.507, Sn=0.240, F1=0.325, MCC=0.062, Acc=0.556, AUC=0.540; LBCE-BERT (LBtope): Precision=0.586, Sn=0.563, F1=0.575, MCC=0.243, Acc=0.627, AUC=0.667; LBCE-BERT (iECB-EL): Precision=0.716, Sn=0.710, F1=0.713, MCC=0.482, Acc=0.744, AUC=0.820<br>**Independent Test Set:** LBCE-BERT (BCPreds): Precision=0.514, Sn=0.234, F1=0.322, MCC=0.074, Acc=0.565, AUC=0.545; LBCE-BERT (LBtope): Precision=0.599, Sn=0.595, F1=0.597, MCC=0.282, Acc=0.646, AUC=0.706; LBCE-BERT (iECB-EL): Precision=0.722, Sn=0.731, F1=0.726, MCC=0.508, Acc=0.757, AUC=0.828	N/A
**Deshmukh et al., 2024 [17]**	**Rep. Learning:** ACC + DC + MBA + MA + GA<br>**Classifier:** Dilated 1-D CNN	**Datasets:** 1. Zika virus, 2. Dengue virus<br>**Type:** Private<br>**Validation:** Not mentioned	**Zika:** Acc=0.83, Precision=0.87, Recall=0.83, F1=0.84<br>**Dengue:** Acc=0.85, Precision=0.87, Recall=0.85, F1=0.86	Liu F. et al. (2024), Ras-Carmona A et al. (2022), Khanna D. et al. (2020), Collatz et al. (2020), Qi Y. et al. (2023)
**Kumar et al., 2024 [23]**	**Rep. Learning:** BLAST + Dipeptide composition (DPC)<br>**Classifier:** Random Forest	**Datasets:** Kumar et al. Dataset<br>**Type:** Public<br>**Validation:** 5-fold CV	**Results:** AUC = 0.829	iBCEEL, ABCPred, LBTope, CBTope, BCEPred, BepiPred3.0, iLBE, EpiDope, BepiBlast, SEMA, BCEPS

Table S2: A Comprehensive Performance Analysis of Linear B cell Epitope Predictors. **Note:** Performance values in the "Performance Results" column are reported as originally published by each study. They derive from heterogeneous datasets, data splits, class-balance ratios, and evaluation protocols; they are *not* directly comparable across studies. Readers should consult the "Dataset & Evaluation" column for context before drawing cross-study inferences.

Author, Year	Method Details	Dataset & Evaluation	Performance Results	Comparisons
**Yuan et al., 2023 [27]**	**Rep. Learning:** BLOSUM62 + BiLSTM + Transformer<br>**Classifier:** MLP	**Datasets:** 1. Yuan et al., 2. NetBCE<br>**Type:** Public<br>**Validation:** Train-test split	**Yuan dataset:** Acc=0.7729, AUC=0.8580, Acc(Ig)=0.7221<br>**NetBCE:** Acc=0.7481, AUC=0.8610	LBtope, IgPred, iBCE-EL, DLBEpitope, EpiDope, AbCPE, NetBCE
**Liu et al., 2023 [19]**	**Rep. Learning:** BERT + AAP + AAC + AAT<br>**Classifier:** XGBoost	**Datasets:** iBCE-EL (Core, IND)<br>**Type:** Public<br>**Validation:** 80% Train, 20% Test, 5-fold CV	**Independent Test:** Acc=0.769, Sp=0.771, AUC=0.838, MCC=0.519<br>**5-fold CV:** Acc=0.769, Sp=0.787, AUC=0.845, MCC=0.534	EpitopeVec, iLBE, LBtope, iBCE-EL
**Qi et al., 2023 [7]**	**Rep. Learning:** Integer Encoding<br>**Classifier:** BiLSTM + CNN + MLP	**Datasets:** Core: BCETD555 (Ras-Carmona), IND: ILED2195, IDED1246<br>**Type:** Public<br>**Validation:** 80% Train, 20% Test, 10-fold CV	**IND Test 1:** Acc=0.64, Sn=0.56, Sp=0.73, MCC=0.29<br>**IND Test 2:** Acc=0.60, Sn=0.75, Sp=0.67, MCC=0.35<br>**10-fold CV:** Acc=0.77, Sn=0.78, Sp=0.75, MCC=0.54	IBCE-EL, LBtope, BepiPred, LBCEPred, BCEPS
**Da et al., 2023 [2]**	**Rep. Learning:** Graph-based signature approach<br>**Classifier:** Random Forest	**Datasets:** 1. BCPred, 2. ABCPred-1, 3. ABCPred-2, 4. Chen, 5. LBtope, 6. iBCE-EL, 7. epitope1D<br>**Type:** Public<br>**Validation:** 80% Train, 20% Test, 10-fold CV	**BCPred:** MCC=0.720, AUC=0.920, F1=0.850<br>**ABCPred-1:** MCC=0.667, AUC=0.823, F1=0.798<br>**Chen:** MCC=0.815, AUC=0.907, F1=0.909<br>**epitope1D:** MCC=0.608, AUC=0.935, F1=0.654	EpitopeVec, EpiDope, BepiPred-3.0 [33]
**Attique et al., 2023 [10]**	**Rep. Learning:** LAFV + RLAFV + FDV + APOV + RAPOV<br>**Classifier:** CNN + MLP	**Datasets:** BepiPred-2.0<br>**Type:** Public<br>**Validation:** 70% Train, 30% Test, 10-fold CV, LOOCV	**Train-test:** Acc=0.878, Sp=0.889, Recall=0.857, Precision=0.832, F1=0.871, AUC=0.945, MCC=0.743<br>**10-fold CV:** Acc=0.879, Sp=0.907, Sn=0.835, Precision=0.851, AUC=0.941, MCC=0.745	EpiDope, BepiPred-2.0
**Amaya et al., 2023 [6]**	**Rep. Learning:** Hydrophobicity, electrostatic charges, SASA, side chain flexibility<br>**Classifier:** Extra Trees	**Datasets:** Amaya et al. dataset<br>**Type:** Private<br>**Validation:** 80% Train, 20% Test, 10-fold CV	**Independent:** AUC=0.985, AUC-PR=0.98, MCC=0.82<br>**10-fold CV:** AUC=0.979±0.005, AUC-PR=0.958±0.010, MCC=0.829±0.025	DiscoTope-3.0
**Angaitkar et al., 2023 [3]**	**Rep. Learning:** Unigram + Bigram + Trigram features<br>**Classifier:** KNN + RF + LogR + SVM	**Datasets:** Angaitkar et al. dataset<br>**Type:** Public<br>**Validation:** NA	**Results:** Acc=99.03, Precision=66.65, Recall=65.82, AUC=99.88, F1=66.23	EnsembleDL, iLEB, SVM
**Angaitkar et al., 2023 [9]**	**Rep. Learning:** AAC + CTD + NMBA + Moran + Geary autocorrelation<br>**Classifier:** KNN	**Datasets:** 1. LBTope-Fixed, 2. ABCPred16, 3. BCPred, 4. Chen<br>**Type:** Public<br>**Validation:** NA	**LBTope-Fixed:** Acc=88.31, Precision=0.821, Recall=0.74, F1=0.88, MCC=0.837<br>**ABCPred16:** Acc=87.52, Precision=0.85, Recall=0.80, F1=0.86, MCC=0.834<br>**Chen:** Acc=91.28, Precision=0.89, Recall=0.77, F1=0.89, MCC=0.901	iBCE-EL, BepiPred-2.0, EpiDope, EpitopeVec
**Xu et al., 2022 [28]**	**Rep. Learning:** 14 features (5 sequence-derived, 6 physicochemical clusters, 3 structural)<br>**Classifier:** CNN + BiLSTM + MLP	**Datasets:** NetBCE dataset<br>**Type:** Public<br>**Validation:** 90% Train, 10% Test, 5-fold CV	**Independent:** AUC=0.8400, PR-AUC=0.6062<br>**5-fold CV:** Avg. AUC=0.8455, PR-AUC=0.6165	BepiPred, LBtope, iBCE-EL, EpiDope
**Sahu et al., 2022 [16]**	**Rep. Learning:** APC + AC<br>**Classifier:** Random Forest	**Datasets:** 1. D40, 2. D100, 3. LBtope_Confirm<br>**Type:** Public<br>**Validation:** 80% Train, 20% Test, 10-fold CV	**Independent:** Acc=0.8333, Sn=0.8737, Sp=0.7950, AUC=0.9135, MCC=0.6697	LBtope, iBLE, iBCE-EL, EpiDope, EpitopeVec
**Ras et al., 2022 [14]**	**Rep. Learning:** NA<br>**Classifier:** NA	**Datasets:** Carmona et al. Datasets (1,2,3,4,5,6)<br>**Type:** Public<br>**Validation:** NA	**Results:** NA	NA

Table S2: A Comprehensive Performance Analysis of Linear B cell Epitope Predictors. **Note:** Performance values in the "Performance Results" column are reported as originally published by each study. They derive from heterogeneous datasets, data splits, class-balance ratios, and evaluation protocols; they are *not* directly comparable across studies. Readers should consult the "Dataset & Evaluation" column for context before drawing cross-study inferences.

Author, Year	Method Details	Dataset & Evaluation	Performance Results	Comparisons
**Yin et al., 2022 [32]**	**Rep. Learning:** ProtVec + QR decomposition + AAindex + CTD transformation **Classifier:** XGBoost	**Datasets:** Yin et al. dataset **Type:** Public **Validation:** 90% Train, 10% Test, 5-fold CV	**Independent:** AUC=0.827, AUC-PR=0.831 **5-fold CV:** Acc=0.753, Precision=0.791, Recall=0.695, F1=0.740, AUC=0.754, AUC-PR=0.825	BepiPred2, LBtope, iBCE-EL, EpitopeVec
**Alghamdi et al., 2022 [11]**	**Rep. Learning:** PRIM + RPRIM + Frequency vector + AAPIM + RAAPIM **Classifier:** Random Forest	**Datasets:** BepiPred-2.0 **Type:** Public **Validation:** 70% Train, 30% Test, 10-fold CV, LOOCV	**Independent:** Acc=0.868, Sn=0.862, Sp=0.872, MCC=0.725, AUC=0.934 **10-fold CV:** Acc=0.868, Sn=0.863, Sp=0.870, MCC=0.725, AUC=0.931	EpiDope, BepiPred2
**Gupta et al., 2022 [24]**	**Rep. Learning:** Hydrophobicity, aliphatic index, sequence length, kidera length, molecular weight, peptide charge, neighbors **Classifier:** Ensemble (DT + Multi-LogR + RF + SVM + AdaBoost)	**Datasets:** LBtope **Type:** Public **Validation:** 70% Train, 30% Test, 7-fold CV	**Results:** Acc=0.8206, Gini=0.683, Sn=0.738, Sp=0.92, Precision=0.917, AUC=0.842	N/A
**Kim et al., 2022 [13]**	**Rep. Learning:** Transformer Encoder + AAindex + 14 Physicochemical Properties **Classifier:** ResNet-1D + MLP	**Datasets:** BepiPred-2.0, EpiDope **Type:** Public **Validation:** 5-fold CV	**Evaluation:** AUC=0.613	N/A
**La et al., 2022 [21]**	**Rep. Learning:** 14 physicochemical properties from AAindex + amino acid frequencies **Classifier:** FAM ANN	**Datasets:** 1. La et al. dataset, 2. IND: BepiPred-2.1 **Type:** Private **Validation:** 5-fold CV	**Independent:** Sn=0.8187, Sp=0.7475, Acc=0.7827, MCC=0.56, AUC=0.7831, PR-AUC=0.83 **5-fold CV:** Acc=0.9149, Sn=0.9150, Sp=0.9149, MCC=0.83, AUC=0.9289	EpiDope, BepiPred-2.0, Chou-Fasman, IUPred, Random
**Ozger et al., 2022 [29]**	**Rep. Learning:** NA **Classifier:** GCCL + GBML + CHI + W + SLAVE	**Datasets:** 1. SARS-CoV epitopes, 2. SARS-CoV-2 peptides **Type:** Public **Validation:** Repeated 6 times	**SARS-CoV:** Avg. Acc=0.917, Average error=8.33	N/A
**Yang et al., 2021 [25]**	**Rep. Learning:** 4 physicochemical properties (hydrophilicity, flexibility, antigenicity, polarity) **Classifier:** Random Forest	**Datasets:** Lbtope_Fixed_non_redundant **Type:** Private **Validation:** 5-fold CV	**Results:** Acc=0.985, Sn=0.978, Sp=0.993, AUC=0.971	NA
**Lim et al., 2021 [26]**	**Rep. Learning:** K-mer composition **Classifier:** SVM	**Datasets:** Lim et al. dataset **Type:** Private **Validation:** 5-fold CV	**Overall:** Acc=0.991 **Bacteria:** Acc=0.814, Sn=0.4537, Sp=0.9482, PPV=0.7649, MCC=0.49, AUC=0.84 **Fungi:** Acc=0.817, Sn=0.9619, Sp=0.5971, PPV=0.7840, MCC=0.62, AUC=0.88 **Viruses:** Acc=0.713, Sn=0.6245, Sp=0.7896, PPV=0.7111, MCC=0.18, AUC=0.78	ABCPred, BCEPred, LEPS, SVMtrip, BCPred, LBtope
**Ghoshal et al., 2021 [31]**	**Rep. Learning:** Variational Bayesian Inference (VBI) **Classifier:** Bayesian Neural Network	**Datasets:** Velpula et al. Dataset **Type:** Public **Validation:** LOOCV	**Results:** In images	N/A
**Ras et al., 2021 [8]**	**Rep. Learning:** AAC + Dipeptide Composition + Combined Composition **Classifier:** SVM	**Datasets:** Core: BCETD555, IND: ILED2195, IDED1246 **Type:** Public **Validation:** 10-fold CV	**Core 10-fold CV:** Acc=0.7538±0.502, Sn=0.73±0.06, Sp=0.78±0.06, MCC=0.51±0.10 **IND ILED2195:** Acc=0.6038, Sn=0.50, Sp=0.71, MCC=0.21 **IND IDED1246:** Acc=0.6705, Sn=0.63, Sp=0.71, MCC=0.34	BepiPred, LBtope, IBCE-EL

Table S2: A Comprehensive Performance Analysis of Linear B cell Epitope Predictors. **Note:** Performance values in the "Performance Results" column are reported as originally published by each study. They derive from heterogeneous datasets, data splits, class-balance ratios, and evaluation protocols; they are *not* directly comparable across studies. Readers should consult the "Dataset & Evaluation" column for context before drawing cross-study inferences.

Author, Year	Method Details	Dataset & Evaluation	Performance Results	Comparisons
**Ashford et al., 2021 [18]**	**Rep. Learning:** AAC + dipeptide composition + conjoint triad **Classifier:** Random Forest	**Datasets:** 1. O. volvulus, 2. Epstein-Barr, 3. Hep. C virus **Type:** Public **Validation:** 75% Train, 25% Test	**Epstein-Barr:** Acc=0.72±0.01, AUC=0.74±0.02, MCC=0.32±0.03, PPV=0.73±0.02, NPV=0.67±0.04, Sn=0.92±0.01 **O. volvulus:** Acc=0.75±0.01, AUC=0.83±0.01, MCC=0.51±0.02 **Hep. C:** Acc=0.75±0.02, AUC=0.8±0.02, MCC=0.47±0.04	ABCPred, BepiPred2, LBtope, IBCE-EL, SVMtrip
**Bahai et al., 2021 [4]**	**Rep. Learning:** AAC + DPC + AAP antigenicity + AAT antigenicity + K-mer + ProtVec **Classifier:** SVM	**Datasets:** 1. BCPred, 2. ABCPred16, 3. Chen, 4. Blind387, 5. LBTope_fixed_nr, 6. iBCE-EL-core, 7. iBCE-EL-IND, 8. Bahai Viral **Type:** Public **Validation:** 5-fold CV	**Results:** Not fully detailed	BCPreds, LBTope, AAP
**Tung et al., 2021 [30]**	**Rep. Learning:** Monoamino acid composition, epitope protein sequence length, diamino acid composition, CTD **Classifier:** Genetic Algorithm	**Datasets:** Tung et al. Dataset **Type:** Private **Validation:** 80% Train, 20% Test	**IgA:** Precision=85.4, Recall=85.3, Acc=85.3, MCC=70.7 **IgE:** Precision=83.9, Recall=83.9, Acc=83.9, MCC=67.9 **IgG:** Precision=98.2, Recall=98.2, Acc=98.1, MCC=91.1	BCIgEPred, NIgPred
**Collatz et al., 2020 [12]**	**Rep. Learning:** ELMo **Classifier:** LSTM	**Datasets:** BepiPred-2.0 **Type:** Public **Validation:** 10-fold CV	**Training:** AUC=0.670 **Test:** AUC=0.625	Betaturn, Accessibility, Antigenicity, Hydrophobicity, IUPred, BepiPred2.0, Random
**Yang et al., 2020 [15]**	**Rep. Learning:** Hydrophobicity + Position Entropy of Amino Acids **Classifier:** LSTM	**Datasets:** 1. Yang et al., 2. Chen et al. **Type:** 1. Private, 2. Public **Validation:** 5-fold CV	**Yang dataset:** Sens=96.79, Spec=78.03, Acc=88.94, MCC=77.63, F1=91.05 **Chen dataset:** Sens=97.48, Spec=75.80, Acc=88.40, MCC=76.75, F1=90.71	SVM, ELMAN
**Liu et al., 2020 [5]**	**Rep. Learning:** DPC **Classifier:** Ensemble Deep Neural Network	**Datasets:** 1. IEDB16, 2. IEDB18, 3. IEDB20, 4. IEDB22, 5. IEDB31, 6. IEDB38, 7. Lbtope_Fixed, 8. ABCpred16 **Type:** Public/Private **Validation:** Not mentioned	**AUC Results:** IEDB16=88.80, IEDB18=89.98, IEDB20=90.85, IEDB22=91.58, IEDB31=93.95, IEDB38=95.68, Lbtope_Fixed=73.83, ABCpred16=65.5	DLBEpitope16-test, DLBEpitope18-test, DLBEpitope20-test, APCpred, BepiPred1.0, BepiPred2.0, DLBEpitope
**Hasan et al., 2020 [20]**	**Rep. Learning:** AIP + AFC + PSSM + PKAF **Classifier:** RF + LR	**Datasets:** iBCE-EL (Core, IND) **Type:** Public **Validation:** 80% Train, 20% Test, 10-fold CV	**Independent:** Acc=0.748, Sp=0.745, Sn=0.752, MCC=0.494, AUC=0.813 **10-fold CV:** Acc=0.752, Sp=0.747, Sn=0.759, MCC=0.496, AUC=0.809	LBtope, iBCE-EL
**Kozlova et al., 2018 [22]**	**Rep. Learning:** Biochemical attributes based approaches **Classifier:** Decision Tree	**Datasets:** Kozlova et al. dataset **Type:** Public **Validation:** 10-fold CV	**Results:** AUC=0.6306, Acc=0.7876, Sn=0.4189, FPR=0.1577, Precision=0.2852, Sp=0.8423	N/A

Table S3: Key benchmark datasets and corpora across the ten T-cell epitope prediction sub-tasks (T1–T10). Sizes are approximate and as reported by the source studies. MHC class indicates the predominant restriction (class I, class II, or both). "Public" denotes an openly downloadable resource, whereas consortium/clinical resources may require controlled access. Sub-tasks T1–T7 apply to any antigen; T8 and T9 are specific to the oncology setting; and T10 spans infectious-disease and cancer targets.

**Sub-task**	**Dataset / corpus**	**Scale**	**MHC class**	**Accessibility**
T1	DEEP-HLA reference panels [34]	Japanese n=1,118 + T1DGC n=5,122; applied to BioBank Japan (n=62,387) / UK Biobank (n=354,459)	—	Public
T1	HLARIMNT reference panels [35]	Pan-Asian n=530 + T1DGC n=5,225	—	Public
T1	CookHLA reference panel [36]	9,773 / 918 individuals (T1DGC, 1000G, HapMap, pan-Asian panels)	—	Public
T1	Lhotte NGS imputation set [37]	61,393 typings (local NGS)	—	Public
T2	NetCTLpan pathway data [38]	504 train ligand pairs; 889+806 test ligands; 216 HIV pairs	Class I	Public
T2	Dorigatti cleavage set [39]	1,285,659 N-term (229,163 pos.); 1,277,344 C-term (222,181 pos.)	Class I	Public
T2	Ziegler cleavage benchmark [40]	1,434,989 N-term; 1,419,501 C-term (after dedup.)	Class I	Public
T2	APLSuite benchmark [41]	13 small proteins (63–117 aa); Varicella-Zoster gE antigen	Class II	Public
T3	NetMHCpan-4.1 BA/EL corpus [42]	$>10^5$ ligands, hundreds of alleles	Class I	Public
T3	MHCflurry 2.0 MS ligand set [43]	Pan-allelic (∼14,993 alleles)	Class I	Public
T3	Ranking-CNN dataset [44]	53,253 peptides / 128 alleles	Class I	Public
T3	DeepNeoAGNet [45]	121,787 peptide–HLA pairs (4 alleles)	Class I	Public
T4	NetMHCIIpan-4.0 BA/EL corpus [42]	4,086,230 data points; 116 class-II molecules (DR/DQ/DP)	Class II	Public

Continued on next page

Table S3 – *Continued from previous page*

Sub-task	Dataset / corpus	Scale	MHC class	Accessibility
T4	MixMHC2pred-2.0 immunopeptidome (Racle 2023) [46]	627,013 unique ligands; 346 samples (DP=79, DQ=52)	Class II	Public
T4	MoDec HLA-II peptidome (Racle 2019) [47]	77,189 unique peptides; 23 cell lines/tissues	Class II	Public
T4	NNAlign_MA training set (Alvarez 2019) [48]	55,178 BA + 32,510 SA-EL + 15,494 MA-EL	Class II	Public
T4	Garde et al. EL+BA set [49]	51,269 BA (31 alleles) + 142,267 EL (25 alleles)	Class II	Public
T4	Andreatta et al. pan-specific BA [50]	52,062 BA (train) + 9,860 eval + 51 PDB	Class II	Public
T4	Wan multi-scale corpus (2025) [51]	141K BA + 1.2M EL + 219K antigen pairs	Class II	Public
T5	DeepHLApan IEDB set [52]	327,178 HLA–peptide pairs (32,785 immunogenicity-labelled)	Class I	Public
T5	DeepImmuno IEDB [53]	8,971 (4,059 pos / 4,912 neg); + TESLA, Dengue, SARS-CoV-2 external	Class I	Public
T5	PRIME compiled set [54]	4,958 peptides (pathogen + cancer); + 409 murine neo	Class I	Public
T5	Repitope (Ogishi) [55]	21,162 HLA-I + 31,693 HLA-II peptides; 23M public CDR3β	Class I/II	Public
T5	ImmunoStruct (IEDB + CEDAR) [56]	~27,000 peptide–MHC pairs	Class I	Public
T5	Stadelmaier IEDB set [57]	14,707 pos / 20,904 neg; 100 HLA alleles	Class I/II	Public
T5	NeoTImmuML (TumorAgDB2.0) [58]	10,312 (5,156/5,156); + 1,086 independent	Class I	Public
T5	ITSNdb (immunogenicity test) [59]	Curated validated test set	Class I	Public
T6	VDJdb [60]	>90,000 TCR–epitope pairs	Class I (II rare)	Public
T6	ActiveTCR [61]	216,054 TCR–pMHC pairs	Class I	Public
T6	UniPMT [62]	156,844 pairs	Class I	Public
T6	TCR-H dataset [63]	~200,000 TCR–pMHC entries	Class I	Public
T6	epiTCR dataset [64]	~9,000+ pairs	Class I	Public

Continued on next page

Table S3 – *Continued from previous page*

Sub-task	Dataset / corpus	Scale	MHC class	Accessibility
T6	IMMREP22 / Hi-TpH benchmarks [65, 66]	Community evaluation splits	Class I	Public
T7	PDB TCR:pMHC complexes [67]	∼hundreds of solved structures	Class I (II rare)	Public
T7	STCRDab [68]	∼900+ curated TCR structures	Class I/II	Public
T7	TCR3d (TCRmodel2 templates) [69]	884 Class I + 44 Class II pMHC templates	Class I/II	Public
T7	ATLAS (TCRcost) [70]	121 precise TCR:pMHC structures (+ AF2 1×–5× augmentation)	Class I	Public
T7	NetTCRstruc scoring set [71]	55 targets / ∼16,541 models (train); 25 targets / 3,750 (benchmark)	Class I	Public
T7	NetTCRstruc specificity / IMMREP23 [71]	17,670 (2,945 binders + 14,725 swapped) over 26 peptides; 246 unseen	Class I	Public
T7	TCRdock benchmark [72]	130 ternary structures + decoy set (8 epitopes, 50 TCRs each)	Class I	Public
T7	TCRmodel2 benchmark [73]	48 + 20 structures; 414 Class I / 47 Class II (VDJdb eval)	Class I/II	Public
T8	pTuneos IEDB immunogenicity set [74]	5,018 immunogenic / 8,227 non-immunogenic 9–11-mers; 2,191 curated training peptides	Class I	Public
T8	NeoGuider NCI discovery cohort [75]	57 patients (82 pos. / 4,975 neg.); + TESLA (34/501), HiTIDE, Wu validation	Class I	Public
T8	CNNeoPP training/validation [76]	1,498 validated peptides; TESLA 532 (5 patients); 153 lung-cancer validation	Class I	Public
T8	TruNeo mass-spectrometry set [77]	74-patient MS HLA peptidome (train); 1,599 SNVs / 13 patients (test)	Class I	Public
T8	ASNP splicing cohorts [78]	GSE37765 (12 samples / 6 NSCLC); GSE22260 (prostate)	Class I	Public
T8	Shi scRNA-seq set [79]	30,342 $CD8^+$ T cells / 10 gastrointestinal-cancer donors	—	Public

Continued on next page

Table S3 – *Continued from previous page*

Sub-task	Dataset / corpus	Scale	MHC class	Accessibility
T8	TESLA validated neoantigens [80]	∼10–53 validated peptides/study	Class I	Controlled
T8	TCGA / ICGC / COSMIC inputs [81, 82, 83]	Cohort somatic variants	—	Public
T9	Charoenkwan / Herrera / Sotirov [84, 85, 86]; derived LYnet / PepBenchmark [87, 88]; private TTAgP [89]	985 / 1,184 / 424; 2,169 / 1,064; 737 sequences	I	Public (8 total: 5 benchmark + 2 independent test, all public; TTAgP private)
T10	DeepVacPred [90]	706,970 positive / 706,970 negative peptide subunits (2,000 T-cell × 5,000 B-cell IEDB epitopes)	I & II / B	Public
T10	Vaxign-ML [91]	575 protective antigens (397 bacterial, 178 viral) / 4,979 negatives (Protegen, UniProt)	Whole protein	Public
T10	SHASI-ML [92]	253/253 train, 64/64 test, 1,806 independent (*Salmonella*; NCBI, UniProtKB)	Whole protein	On request

Table S4: Representative T-cell prediction methods per sub-task, with representation learning, classifier, headline performance, and code availability. **Note:** headline performance values are reported as originally published by each study; they derive from heterogeneous datasets, splits, and evaluation protocols and are *not* directly comparable across studies or sub-tasks. Sub-tasks T1–T7 apply to any antigen; T8 and T9 are specific to the oncology setting; and T10 spans infectious-disease and cancer targets.

**Sub-task**	**Method (year)**	**Representation learning**	**Classifier**	**Headline (not comparable)**	**Code**
T1	CookHLA (2021) [36]	Binary exon-embedded allele markers	HMM imputation (Beagle + adaptive map)	93–99% conc. (class-I high)	GitHub
T1	DEEP-HLA (2021) [34]	One-hot phased SNV haplotypes	Multitask CNN	Sens 0.987 / PPV 0.986 (Japanese ref.)	GitHub
T1	HLARIMNT (2022) [35]	Chunked 2D SNP tokens + cls token	Transformer (multi-head attn.)	r^2 0.85 / PPV 0.87 (Pan-Asian); >DEEP-HLA on rare alleles	GitHub
T2	NetCTLpan (2010) [38]	Peptide seq. (8–11-mer) + NetChop/TAP	Weighted feed-forward NN	AUC 0.98 (9-mer test)	Web
T2	Dorigatti et al. (2022) [39]	One-hot (240-dim window)	MLP ensemble (PUUPL, PU-learning)	AUROC 0.87 C-/0.78 N-term	GitHub
T2	Ziegler et al. (2022) [40]	BiLSTM (ESM2/ProtT5 explored)	Bidirectional LSTM	AUC 88.5% C-/79.5% N-term	GitHub
T2	APLSuite (2026) [41]	Structural stability (B-factor, SASA, COREX, entropy)	Likelihood + NetMHC-II	Qualitative (CD4/MHC-II)	GitHub
T3	NetMHCpan-4.1 (2020) [42]	BLOSUM + EL motif	Ensemble NN	AUC >0.9	Web
T3	MHCflurry 2.0 (2020) [43]	One-hot + processing	Ensemble NN	AUC >0.9	GitHub
T3	BigMHC (2023) [93]	ESM PLM embeddings	DL / transfer	High AUPRC	GitHub
T3	3pHLA-score (2022) [94]	Structure energy terms	Random Forest	AUPRC 0.82→0.99	GitHub
T3	DeepMHC (2017) [95]	One-hot 2D multi-channel	Deep CNN	Avg AUC 0.73 (9-mer)	Web
T3	ConvMHC (2017) [96]	Image-like array (34×9, 18 ch)	Deep CNN	5-CV acc. 0.892	Web/GitHub
T3	BVLSTM-MHC (2021) [97]	One-hot + BLOSUM	BiLSTM RNN	ACC 0.955, AUC 0.951	Web

Continued on next page

Table S4 – *Continued from previous page*

Sub-task	Method (year)	Representation learning	Classifier	Headline (not comparable)	Code
T3	DPCMHC (2025) [98]	Mod. one-hot + 34-mer pseudo-seq	Dual-pad CNN + BiLSTM	AUC 0.88	GitHub
T3	DeepNeoAGNet (2025) [45]	AAC/DC + local AAC/DC + windows	Global–local hetero. BiLSTM (regression)	Test R^2 0.29, RMSE 1.65; +21–31% R^2 vs HLAIImaster	GitHub
T3	Hashemi et al. (2023) [99]	ESM2-3B embeddings (NLP framing)	Softmax / GAT	PPV 0.86, AUC 0.98	Web
T3	HLApollo (2024) [100]	Tokenized pep+flanks+MHC, ESM1b feats	Transformer encoder	AP 74.8% (BM1)	Zenodo
T3	CMHS (2026) [101]	BLOSUM50 + ESM2 (LoRA) + ESM3 struct	GAT/GCN/CNN/MLP	AUROC 0.94, PCC 0.85	GitHub
T3	TransBindpMHCI (2026) [102]	AA emb. + MHC pseudo-seq + pos. enc.	Dual-tier transformer	Acc. 0.95 (DMVD)	GitHub+web
T4	NetMHCIIpan-3.1 / Andreatta (2015) [50]	BLOSUM50 + PFR + length	Ensemble feed-forward ANN	45/51 binding cores correct	Web
T4	Trans-allelic / Degoot (2018) [103]	Biophysical Hamiltonians	GLM + L1 (Lasso)	AUC 0.93/0.83/0.80 (DP/DQ/DR)	—
T4	Garde et al. (2019) [49]	BLOSUM50 log-odds + flank/length	NNAlign ANN (dual-output)	+9.6% F-rank vs IIpan-3.1 (EL)	—
T4	NNAlign_MA / Alvarez (2019) [48]	NNAlign BLOSUM encoding	ANN (NNAlign_MA)	$CD4^+$ Frank 0.024	Web
T4	MoDec / MixMHC2pred (2019) [47]	Probabilistic motif deconvolution	PWM specificity model	AUC 0.83 (IEDB $CD4^+$ tetramer)	GitHub
T4	NetMHCIIpan-4.0 (2020) [42]	NNAlign_MA (BLOSUM)	Ensemble feed-forward NN	$CD4^+$ Frank 0.0351	Web
T4	DeepSeqPanII (2022) [104]	One-hot + BLOSUM62	LSTM-CNN + attention	AUC >0.7 on 44/54 alleles	GitHub
T4	MTL4MHC2 (2022) [105]	Word2Vec + ProtVec	Multi-task BiLSTM	AUC 0.82; acc. ∼0.75 (IEDB)	GitHub
T4	Structure-aware / Yu (2024) [106]	Positional encoding + pep. embed.	1-D CNN (DeepMHCII)	AUC 0.857 (BD2016)	GitHub
T4	MixMHC2pred-2.0 / Racle (2023) [46]	PPM + one-hot contacts + BLOSUM62	Feed-forward NN	ROC-AUC > IIpan-4.0 ($CD4^+$)	GitHub+web
T4	Wan et al. (2025) [51]	ESM2 + AlphaFold3 + physicochem.	1D-conv + self/cross-attention	BA-AUC 0.76; EL-AUC 0.85	—

Continued on next page

Table S4 – *Continued from previous page*

Sub-task	Method (year)	Representation learning	Classifier	Headline (not comparable)	Code
T5	DeepHLApan (2019) [52]	One-hot + HLA pseudo-seq (34 aa)	Stacked BiGRU + attention	Best in 6/11 IEDB subsets; +44% TCGA precision	GitHub+web
T5	Repitope (2019) [55]	TCR–peptide contact-potential profiling	Extremely Rand. Trees	Consistent CV (norm. SD 5–8%)	GitHub
T5	Riley et al. (2019) [107]	117 Rosetta structure energy terms	Feed-forward ANN	CV AUC 0.69; neoantigen test 0.60	PDB only
T5	Weber et al. (2020) [108]	One-hot + MD-derived SASA channels	Deep CNN	5-fold AUC 0.89; neoantigen 0.71	—
T5	PRIME (2021) [54]	21-dim: %rank affinity + AA freq. at contact pos.	Logistic regression	> NetMHC-pan/MHCflurry (AUROC/PRAUC)	GitHub+web
T5	DeepImmuno (2021) [53]	AAindex PCA (566 physchem) + GAN aug.	CNN	IEDB AUROC 0.85, AUPR 0.81; TESLA 29/35	GitHub+web
T5	Stadelmaier (2024) [57]	One-hot + learned emb. + pos. enc.	Transformer + adversarial domain adapt.	Human MHC-I AUC 0.62; MHC-II 0.68	GitHub
T5	ImmunoStruct (2025) [56]	AF2 graph + VAE seq + biochem	EGNN + VAE + MLP (multi-modal attn.)	IEDB AUROC 0.882; CEDAR 0.771	GitHub
T5	NeoTImmuML (2025) [58]	78 physicochemical features	Weighted ensemble (LGBM/XGB/RF)	5-fold AUC 0.871; indep. 0.887	Web
T5	Hu et al. (2025) [109]	NetMHCIIpan-4.0 EL cores + clinical factors	Random Forest	CV AUC 0.72 (epitope) → 0.93 (+clinical)	—
T5	NeoPrecis (2026) [110]	Residue emb. + BLOSUM62 + geometric/CRD	NN + logistic reg. (clonality-aware)	Best AUROC on NCI cohort (MHC-I & II)	GitHub
T6	NetTCR-2.0/2.1 (2021/22) [111, 112]	CNN over CDR3	CNN	AUC 0.6–0.85	GitHub
T6	ERGO-II (2021) [113]	Autoencoder embedding	LSTM	Competitive	GitHub
T6	pMTnet (2021) [114]	AE embedding + transfer learning	DL	High (seen)	GitHub
T6	TCR-ESM (2024) [115]	ESM-1v embeddings	Deep learning (MLP)	MCC-based; >NetTCR-2.0/ERGO-II	GitHub
T6	TULIP (2024) [116]	Transformer LM	Transformer	0.7–0.8 (unseen)	GitHub
T6	epiTCR (2023) [64]	Physicochemical	Random Forest	Up to ∼0.9 (seen)	GitHub

Continued on next page

Table S4 – *Continued from previous page*

Sub-task	Method (year)	Representation learning	Classifier	Headline (not comparable)	Code
T6	Ma et al. (2025) [117]	Unified token emb. + sinusoidal pos. enc.	Cross-attention transformer (triad fusion)	Triad ROC-AUC 0.922; unseen 0.733	On publ.
T6	TCR-EML (2025) [118]	Frozen PLM emb. (ProteinBERT/ESM-1b/ESM-2)	Explain-by-design head on PLM	ROC-AUC up to 0.999 (top epitopes)	Web
T6	UnifyImmun (2025) [119]	64-d char emb. + sinusoidal pos. enc.	Cross-attention transformer (HLA+TCR)	pHLA AUROC 0.962; +9% on COVID-19 pTCR	GitHub
T7	TCRdock (2023) [72]	Hybrid TCR:pMHC templates, MSA-free	Fine-tuned AlphaFold2	Decoy AUROC 0.82 (up to 0.97 YLQ/ELA); docking improved in 94%	GitHub
T7	TCRmodel2 (2023) [73]	TCR/MHC-focused MSAs + pMHC templates	Adapted AlphaFold 2.3	Medium/High CAPRI in >50% of 48 cases; >AF2.3 & TCRdock	Web + GitHub
T7	NetTCRstruc (2025) [71]	ESM-IF1 + GVP graph features	GVP-GNN consensus (+AF conf.)	DockQ SCC 0.855; modest on unseen peptides	GitHub
T7	TCRcost (2024) [120]	3D coords + RDKit atom features	CNN+LSTM correction + 3D-CNN	ACC 0.95/AUC 0.97 (precise); 0.76 (realistic)	—
T8	pTuneos (2019) [74]	BLOSUM62 + hydrophobicity + %rank	Random forest (Pre&RecNeo)	Test AUC 0.833; >MuPeXI/Neopepsee (RCS)	GitHub
T8	NeoPredPipe (2019) [121]	NetMHCpan scores + recognition potential	Rule-based (multistate thermodynamic)	RP>1 ranking; intra-tumor heterogeneity	GitHub
T8	TruNeo (2020) [77]	One-hot + HLA embedding (mass spec)	Fully connected NN (256/74)	52.6% recall in top 10 (>MHCflurry/NetMHCpan4)	—
T8	NeoFox (2021) [122]	16 annotated neoantigen features	Annotation toolbox (no ML model)	Feature annotation (not a classifier)	GitHub
T8	ASNP (2021) [78]	DeepHLApan encoding (splicing peptides)	BiGRU + attention (DeepHLApan)	4 / 8 validated epitopes (NSCLC / prostate)	—
T8	Shi et al. (2022) [79]	649 highly variable genes (scRNA-seq)	XGBoost (26-gene signature)	AUC 0.99; AUPRC 0.87 (reactive $CD8^{+}$)	GitHub

Continued on next page

Table S4 – *Continued from previous page*

Sub-task	Method (year)	Representation learning	Classifier	Headline (not comparable)	Code
T8	NeoGuider (2024) [75]	aKDE + isotonic/centered-isotonic reg. (log-odds)	Logistic regression	AUC 0.995 vs DeepHLApan 0.951 (Wu); >25 TESLA teams	GitHub
T8	pVACview (2024) [123]	OpenCRAVAT / Cancer Gene Census annot.	Interactive visualization (no ML model)	Prioritization UI (not a classifier)	GitHub+web
T8	CNNeoPP (2025) [76]	TF-IDF + fine-tuned BioBERT + one-hot	FCNN + BioBERT (multi-modal)	CV AUC 0.81; ELISpot 58% in top 50	Web
T9	iTTCA / PSRTTCA / ENCAP [124, 84, 125]; iTTCA-DNN / LYnet [126, 87]	Hand-crafted descriptors; emerging DL (DNN, CNN–BiLSTM); ESM-2 via general frameworks [127, 88]	ML / ensemble / emerging DL	Acc. 0.72–0.99; LYnet AUC 0.992	Mixed
T10	DeepVacPred (2021) [90]	Z-descriptors + auto-cross-covariance (45-vector)	DNN (multi-layer CNN + dense)	Test AUC 0.97, Acc. 0.95 (in-silico construct)	GitHub
T10	Vaxign-ML (2020) [91]	Reverse-vaccinology biological + physicochemical features	XGBoost	Weighted F1 0.94, MCC 0.66 (5-fold CV)	Web
T10	SHASI-ML (2025) [92]	Global, sequence-derived + structural protein features	XGBoost (800 trees, depth 8)	Acc. 0.85, F1 0.84 (bacterial antigens)	On request

References

[1] Fang Liu, ChengCheng Yuan, Haoqiang Chen, and Fei Yang. Prediction of linear b-cell epitopes based on protein sequence features and bert embeddings. *Scientific Reports*, 14(1):2464, 2024.

[2] Bruna Moreira da Silva, David B Ascher, and Douglas EV Pires. epitope1d: accurate taxonomy-aware b-cell linear epitope prediction. *Briefings in Bioinformatics*, 24(3):bbad114, 2023.

[3] Pratik Angaitkar, Turki Aljrees, Saroj Kumar Pandey, Ankit Kumar, Rekh Ram Janghel, Tirath Prasad Sahu, Kamred Udham Singh, and Teekam Singh. Inferring linear-b cell epitopes using 2-step metaheuristic variant-feature selection using genetic algorithm. *Scientific Reports*, 13(1):14593, 2023.

[4] Akash Bahai, Ehsaneddin Asgari, Mohammad RK Mofrad, Andreas Kloetgen, and Alice C McHardy. Epitopevec: linear epitope prediction using deep protein sequence embeddings. *Bioinformatics*, 37(23):4517–4525, 2021.

[5] Tao Liu, Kaiwen Shi, and Wuju Li. Deep learning methods improve linear b-cell epitope prediction. *BioData mining*, 13:1–13, 2020.

[6] Diego Amaya-Ramirez, Magali Devriese, Romain Lhotte, Cedric Usureau, Malika Smail-Tabbone, Jean-Luc Taupin, and Marie-Dominique Devignes. Hla-epicheck: A b-cell epitope prediction tool for hla proteins using molecular dynamics simulation data. *bioRxiv*, pages 2023–12, 2023.

[7] Yue Qi, Peijie Zheng, and Guohua Huang. Deeplbcepred: A bi-lstm and multi-scale cnn-based deep learning method for predicting linear b-cell epitopes. *Frontiers in Microbiology*, 14:1117027, 2023.

[8] Alvaro Ras-Carmona, Hector F Pelaez-Prestel, Esther M Lafuente, and Pedro A Reche. Bceps: A web server to predict linear b cell epitopes with enhanced immunogenicity and cross-reactivity. *Cells*, 10(10):2744, 2021.

[9] Pratik Angaitkar, Rekh Ram Janghel, and Tirath Prasad Sahu. ghpcso: Gaussian distribution based hybrid particle cat swarm optimization for linear b-cell epitope prediction. *International Journal of Information Technology*, 15(5):2805–2818, 2023.

[10] Muhammad Attique, Tamim Alkhalifah, Fahad Alturise, and Yaser Daanial Khan. Deepbce: evaluation of deep learning models for identification of immunogenic b-cell epitopes. *Computational Biology and Chemistry*, 104:107874, 2023.

[11] Wajdi Alghamdi, Muhammad Attique, Ebraheem Alzahrani, Malik Zaka Ullah, and Yaser Daanial Khan. Lbcepred: a machine learning model to predict linear b-cell epitopes. *Briefings in bioinformatics*, 23(3):bbac035, 2022.

[12] Maximilian Collatz, Florian Mock, Emanuel Barth, Martin Hölzer, Konrad Sachse, and Manja Marz. Epidope: a deep neural network for linear b-cell epitope prediction. *Bioinformatics*, 37(4):448–455, 2021.

[13] Youjin Kim, Jinhee Park, and Junseok Kwon. B-cell linear epitope prediction using transformer encoder. In *2022 13th International Conference on Information and Communication Technology Convergence (ICTC)*, pages 1091–1093. 2022.

[14] Alvaro Ras-Carmona, Alexander A Lehmann, Paul V Lehmann, and Pedro A Reche. Prediction of b cell epitopes in proteins using a novel sequence similarity-based method. *Scientific Reports*, 12(1):13739, 2022.

[15] Hongguang Yang, Bin Cheng, and Lingyun Liu. A novel linear b-cell epitope prediction method based on position entropy of amino acids. In *Proceedings of the 2020 7th International Conference on Biomedical and Bioinformatics Engineering*, pages 149–153. 2020.

[16] Tanmaya Kumar Sahu, Prabina Kumar Meher, Nalini Kanta Choudhury, and Atmakuri Ramakrishna Rao. A comparative analysis of amino acid encoding schemes for the prediction of flexible length linear b-cell epitopes. *Briefings in Bioinformatics*, 23(5):bbac356, 2022.

[17] J.Y. Deshmukh et al. Gaussian-based dilated 1-d cnn for classifying b-cell epitopes in zika and dengue protein sequences. *Journal of Electrical Systems*, 20(1s), 2024.

[18] Jodie Ashford, Joao Reis-Cunha, Igor Lobo, Francisco Lobo, and Felipe Campelo. Organism-specific training improves performance of linear b-cell epitope prediction. *Bioinformatics*, 37(24):4826–4834, 2021.

[19] Yufeng Liu, Yinbo Liu, Shuyu Wang, and Xiaolei Zhu. Lbce-xgb: a xgboost model for predicting linear b-cell epitopes based on bert embeddings. *Interdisciplinary Sciences: Computational Life Sciences*, 15(2):293–305, 2023.

[20] Md Mehedi Hasan, Mst Shamima Khatun, and Hiroyuki Kurata. ilbe for computational identification of linear b-cell epitopes by integrating sequence and evolutionary features. *Genomics, Proteomics & Bioinformatics*, 18(5):593–600, 2020.

[21] Anthony F La Marca, Robson da S Lopes, Anna Diva P Lotufo, Daniella C Bartholomeu, and Carlos R Minussi. Bepfamn: A method for linear b-cell epitope predictions based on fuzzy-artmap artificial neural network. *Sensors*, 22(11):4027, 2022.

[22] Edgar Ernesto Gonzalez Kozlova, Loïc Cerf, Francisco Santos Schneider, Benjamin Thomas Viart, Christophe NGuyen, Bethina Trevisol Steiner, Sabrina de Almeida Lima, Franck Molina, Clara Guerra Duarte, Liza Felicori, et al. Computational b-cell epitope identification and production of neutralizing murine antibodies against atroxlysin-i. *Scientific reports*, 8(1):14904, 2018.

[23] Nishant Kumar, Sadhana Tripathi, Neelam Sharma, Sumeet Patiyal, Naorem Leimarembi Devi, and Gajendra PS Raghava. A method for predicting linear and conformational b-cell epitopes in an antigen from its primary sequence. *Computers in Biology and Medicine*, 170:108083, 2024.

[24] Vishan Kumar Gupta, Avdhesh Gupta, Paras Jain, and Pankaj Kumar. Linear b-cell epitopes prediction using bagging based proposed ensemble model. *International Journal of Information Technology*, 14(7):3517–3526, 2022.

[25] Hongguang Yang, Yunfei Zhou, and Bin Cheng. Prediction of linear b-cell epitopes using manifold adaptive experimental design and random forest algorithm. *2021 IEEE Conference on Telecommunications, Optics and Computer Science (TOCS)*, pages 81–85, 2021.

[26] Li Cen Lim, Yee Ying Lim, and Yee Siew Choong. Data curation to improve the pattern recognition performance of b-cell epitope prediction by support vector machine. *Pure and Applied Chemistry*, 93(5):571–577, 2021.

[27] Xiao Yuan. Beetle: A framework for linear b-cell epitope prediction and classification. *Joint European Conference on Machine Learning and Knowledge Discovery in Databases (ECML PKDD)*, pages 477–494, 2023.

[28] Haodong Xu and Zhongming Zhao. Netbce: an interpretable deep neural network for accurate prediction of linear b-cell epitopes. *Genomics, Proteomics & Bioinformatics*, 20(5):1002–1012, 2022.

[29] Zeynep Banu Ozger and Pınar Cihan. A novel ensemble fuzzy classification model in sars-cov-2 b-cell epitope identification for development of protein-based vaccine. *Applied soft computing*, 116:108280, 2022.

[30] Chi-Hua Tung, Yi-Sheng Chang, Kai-Po Chang, and Yen-Wei Chu. Nigpred: class-specific antibody prediction for linear b-cell epitopes based on heterogeneous features and machine-learning approaches. *Viruses*, 13(8):1531, 2021.

[31] Bhargab Ghoshal, Biraja Ghoshal, Stephen Swift, and Allan Tucker. Uncertainty estimation in sars-cov-2 b-cell epitope prediction for vaccine development. In *Artificial Intelligence in Medicine: 19th International Conference on Artificial Intelligence in Medicine, AIME 2021, Virtual Event, June 15–18, 2021, Proceedings*, pages 361–366. 2021.

[32] Rui Yin, Xianghe Zhu, Min Zeng, Pengfei Wu, Min Li, and Chee Keong Kwoh. A framework for predicting variable-length epitopes of human-adapted viruses using machine learning methods. *Briefings in Bioinformatics*, 23(5):bbac281, 2022.

[33] Joakim Nøddeskov Clifford, Magnus Haraldson Høie, Sebastian Deleuran, Bjoern Peters, Morten Nielsen, and Paolo Marcatili. Bepipred-3.0: Improved b-cell epitope prediction using protein language models. *Protein Science*, 31(12):e4497, 2022.

[34] Tatsuhiko Naito, Ken Suzuki, Jun Hirata, Yoichiro Kamatani, Koichi Matsuda, Tatsushi Toda, and Yukinori Okada. A deep learning method for HLA imputation and trans-ethnic MHC fine-mapping of type 1 diabetes (DEEP-HLA). *Nature Communications*, 12:1639, 2021.

[35] Kaho Tanaka, Kosuke Kato, Naoki Nonaka, and Jun Seita. Efficient HLA imputation from sequential SNPs data by transformer. *Proceedings of Machine Learning for Health (ML4H)*, 2022. Preprint.

[36] Seungho Cook, Wanson Choi, Hyunjoon Lim, Yang Luo, Kwangwoo Kim, Xiaoming Jia, Soumya Raychaudhuri, and Buhm Han. Accurate imputation of human leukocyte antigens with CookHLA. *Nature Communications*, 12:1264, 2021.

[37] Romain Lhotte et al. Improving HLA typing imputation accuracy and eplet identification with local next-generation sequencing. *HLA*, 103(1):e15222, 2024.

[38] Thomas Stranzl, Mette Voldby Larsen, Claus Lundegaard, and Morten Nielsen. NetCTLpan: pan-specific MHC class I pathway epitope predictions. *Immunogenetics*, 62:357–368, 2010.

[39] Emilio Dorigatti, Bernd Bischl, and Benjamin Schubert. Improved proteasomal cleavage prediction with positive-unlabeled learning. *Proceedings of Machine Learning for Health (ML4H)*, 2022. Preprint.

[40] Ingo Ziegler, Bolei Ma, Ercong Nie, Bernd Bischl, David Rügamer, Benjamin Schubert, and Emilio Dorigatti. What cleaves? Is proteasomal cleavage prediction reaching a ceiling? *arXiv*, 2022. Preprint.

[41] Jiarui Li, Marco K Carbullido, Jai Bansal, Samuel J Landry, and Ramgopal R Mettu. APLSuite: an integrated suite for CD4+ T cell epitope prediction via antigen processing likelihood. *arXiv*, 2026. Preprint.

[42] Birkir Reynisson, Bruno Alvarez, Sinu Paul, Bjoern Peters, and Morten Nielsen. NetMHCpan-4.1 and NetMHCIIpan-4.0: improved predictions of MHC antigen presentation by concurrent motif deconvolution and integration of MS MHC eluted ligand data. *Nucleic Acids Research*, 48(W1):W449–W454, 2020.

[43] Timothy J O'Donnell, Alex Rubinsteyn, and Uri Laserson. MHCflurry 2.0: improved pan-allele prediction of MHC class I-presented peptides by incorporating antigen processing. *Cell Systems*, 11(1):42–48, 2020.

[44] Ziqi Chen, Martin Renqiang Min, and Xia Ning. Ranking-based convolutional neural network models for peptide-MHC class I binding prediction. *Frontiers in Molecular Biosciences*, 8:634836, 2021.

[45] Xian-Xian Liu, Jie Yang, et al. DeepNeoAGNet: enhancing cancer immunotherapy with general heterogeneous sequence learning for precise neoantigen prediction and immunogenicity assessment. *Research Square*, 2025. Preprint.

[46] Julien Racle, Philippe Guillaume, Julien Schmidt, Justine Michaux, Amedee Larabi, Kelvin Lau, Marta A S Perez, Giancarlo Croce, et al. Machine learning predictions of MHC-II specificities reveal alternative binding mode of class II epitopes (MixMHC2pred-2.0). *Immunity*, 56(6):1359–1375, 2023.

[47] Julien Racle, Justine Michaux, Georg Alexander Rockinger, Marion Arnaud, Sara Bobisse, et al. Deep motif deconvolution of HLA-II peptidomes for robust class II epitope predictions. *bioRxiv*, 2019. Preprint.

[48] Bruno Alvarez, Birkir Reynisson, Carolina Barra, Søren Buus, Nicola Ternette, Tim Connelley, Massimo Andreatta, and Morten Nielsen. NNAlign_MA; MHC peptidome deconvolution for accurate MHC binding motif characterization and improved T-cell epitope predictions. *Molecular & Cellular Proteomics*, 18(12):2459–2477, 2019.

[49] Christian Garde, Sri H Ramarathinam, Emma C Jappe, Morten Nielsen, Jens V Kringelum, Thomas Trolle, and Anthony W Purcell. Improved peptide-MHC class II interaction prediction through integration of eluted ligand and peptide affinity data. *Immunogenetics*, 71:445–454, 2019.

[50] Massimo Andreatta, Edita Karosiene, Michael Rasmussen, Anette Stryhn, Søren Buus, and Morten Nielsen. Accurate pan-specific prediction of peptide-MHC class II binding affinity with improved binding core identification. *Immunogenetics*, 67:641–650, 2015.

[51] Yue Wan, Jiayi Yuan, Zhiwei Feng, et al. Accelerating MHC-II epitope discovery via multi-scale prediction in antigen presentation. *arXiv*, 2025. Preprint.

[52] Jingcheng Wu, Wenzhe Wang, Jiucheng Zhang, Binbin Zhou, Wenyi Zhao, Zhixi Su, Xun Gu, Jian Wu, Zhan Zhou, and Shuqing Chen. DeepHLApan: a deep learning approach for neoantigen prediction considering both HLA-peptide binding and immunogenicity. *Frontiers in Immunology*, 10:2559, 2019.

[53] Guangyuan Li, Balaji Iyer, V B Surya Prasath, Yizhao Ni, and Nathan Salomonis. DeepImmuno: deep learning-empowered prediction and generation of immunogenic peptides for T-cell immunity. *Briefings in Bioinformatics*, 22(6):bbab160, 2021.

[54] Julien Schmidt, Angela R Smith, Morgane Magnin, Julien Racle, Jason R Devlin, others, Brian M Baker, Alexandre Harari, and David Gfeller. Prediction of neo-epitope immunogenicity reveals TCR recognition determinants and provides insight into immunoediting (PRIME). *Cell Reports Medicine*, 2(2):100194, 2021.

[55] Masato Ogishi and Hiroshi Yotsuyanagi. Quantitative prediction of the landscape of T cell epitope immunogenicity in sequence space (Repitope). *Frontiers in Immunology*, 10:827, 2019.

[56] Kevin Givechian, João Rocha, Edward Yang, Chen Liu, Kerrie Greene, Smita Krishnaswamy, et al. ImmunoStruct: a multimodal neural network framework for immunogenicity prediction from peptide-MHC sequence, structure, and biochemical properties. *Research Square*, 2025. Preprint.

[57] Josua Stadelmaier, Brandon Malone, and Ralf Eggeling. Transfer learning for t-cell response prediction. *arXiv preprint arXiv:2403.12117*, 2024.

[58] Yan Shao, Shuguang Ge, Ruizhe Dong, Wei Ji, Chaoran Qin, and Pengbo Wen. NeoTImmuML: a machine learning-based prediction model for human tumor neoantigen immunogenicity. *Frontiers in Immunology*, 16:1681396, 2025.

[59] Guadalupe Nibeyro, Rocío Flesia, Daniela Orschanski, Agustín Nava, et al. The immunogenic tumor-specific neoantigen database (ITSNdb): A tool for comprehensive performance evaluation of neoantigen immunogenicity predictors. In *Methods in Molecular Biology*, pages 139–156. Springer US, 2025.

[60] Mikhail Shugay, Dmitriy V Bagaev, Ivan V Zvyagin, Renske M Vroomans, et al. VDJdb: a curated database of T-cell receptor sequences with known antigen specificity. *Nucleic Acids Research*, 46(D1):D419–D427, 2018.

[61] Pengfei Zhang, Seojin Bang, and Heewook Lee. Active learning framework for cost-effective TCR-epitope binding affinity prediction (ActiveTCR). *2023 IEEE International Conference on Bioinformatics and Biomedicine (BIBM)*, pages 988–993, 2023.

[62] Yunxiang Zhao, Jijun Yu, Yixin Su, You Shu, Enhao Ma, Jing Wang, et al. A unified deep framework for peptide-major histocompatibility complex-T cell receptor binding prediction (UniPMT). *Nature Machine Intelligence*, 7, 2025.

[63] Rajitha Tatikonda et al. TCR-H: explainable machine learning prediction of T-cell receptor epitope binding on unseen datasets. *Frontiers in Immunology*, 15:1426173, 2024.

[64] My-Diem Nguyen Pham, Thanh-Nhan Nguyen, Le Son Tran, Que-Tran Bui Nguyen, et al. epiTCR: a highly sensitive predictor for TCR–peptide binding. *Bioinformatics*, 39(5):btad284, 2023.

[65] Pieter Meysman, John Barton, Barbara Bravi, et al. Benchmarking solutions to the T-cell receptor epitope prediction problem: IMMREP22 workshop report. *ImmunoInformatics*, 9:100024, 2023.

[66] Fuli Feng, Xinyuan Zhu, Jiadong Lu, Yeqing Lu, Yuyan Zhang, et al. Machine learning dataset and benchmark for accurate T cell receptor-phla binding prediction (Hi-TpH). *Research Square*, 2025. Preprint.

[67] Helen M Berman, John Westbrook, Zukang Feng, Gary Gilliland, T N Bhat, Helge Weissig, Ilya N Shindyalov, and Philip E Bourne. The Protein Data Bank. *Nucleic Acids Research*, 28(1):235–242, 2000.

[68] Jinwoo Leem, Saulo H P de Oliveira, Konrad Krawczyk, and Charlotte M Deane. STCRDab: the structural T-cell receptor database. *Nucleic Acids Research*, 46(D1):D406–D412, 2018.

[69] Ragul Gowthaman and Brian G Pierce. TCR3d: The T cell receptor structural repertoire database. *Bioinformatics*, 35(24):5323–5325, 2019.

[70] Tyler Borrman, Jennifer Cimons, Michael Cosiano, Michael Purcaro, et al. ATLAS: A database linking binding affinities with structures for wild-type and mutant TCR-pMHC complexes. *Proteins: Structure, Function, and Bioinformatics*, 85(5):908–916, 2017.

[71] Sebastian N. Deleuran and Morten Nielsen. NetTCR-struc: a structure-driven approach for prediction of TCR-pmhc interactions. *Frontiers in Immunology*, 16:1616328, 2025.

[72] Philip Bradley. Structure-based prediction of T cell receptor:peptide-MHC interactions. *eLife*, 12:e82813, 2023.

[73] Rui Yin, Helder V Ribeiro-Filho, Valerie Lin, Ragul Gowthaman, Melyssa Cheung, and Brian G Pierce. Tcrmodel2: high-resolution modeling of t cell receptor recognition using deep learning. *Nucleic Acids Research*, 51(W1):W569–W576, 2023.

[74] Chi Zhou, Zhiting Wei, Zhanbo Zhang, Biyang Zhang, Chengyu Zhu, Ke Chen, et al. pTuneos: prioritizing tumor neoantigens from next-generation sequencing data. *Genome Medicine*, 11:67, 2019.

[75] Xiaofei Zhao, Lei Wei, and Xuegong Zhang. NeoGuider: neoepitope prediction using advanced feature engineering. *Research Square*, 2024. Preprint.

[76] Yu Cai, Rui Chen, Mingming Song, Lei Wang, Zirong Huo, others, Yali Cui, and Xi Zhang. CNNeoPP: a deep learning pipeline for personalized neoantigen prediction and liquid biopsy applications. *medRxiv*, 2025. Preprint.

[77] Yi Tang, Yu Wang, Jiangjie Wang, Ming Li, et al. TruNeo: an integrated pipeline improves personalized true tumor neoantigen identification. *BMC Bioinformatics*, 21:532, 2020.

[78] S Mao, J Wen, Y Feng, W Zhao, and X Zhou. ASNP: a personalized alternative splicing neoantigen discovery pipeline. *Annals of Advanced Biomedical Sciences*, 4(2):000170, 2021.

[79] Zhiwen Shi et al. A machine learning model that identifies neoantigen-reactive CD8+ T cells in human gastrointestinal cancer. *Research Square*, 2022. Preprint.

[80] Daniel K Wells, Marit M van Buuren, Kristen K Dang, Vanessa M Hubbard-Lucey, et al. Key parameters of tumor epitope immunogenicity revealed through a consortium approach improve neoantigen prediction. *Cell*, 183(3):818–834, 2020.

[81] John N Weinstein, Eric A Collisson, Gordon B Mills, Kenna R Mills Shaw, Brad A Ozenberger, Kyle Ellrott, Ilya Shmulevich, Chris Sander, and Joshua M Stuart. The Cancer Genome Atlas pan-cancer analysis project. *Nature Genetics*, 45(10):1113–1120, 2013.

[82] Thomas J Hudson, Warwick Anderson, Axel Aretz, et al. International network of cancer genome projects. *Nature*, 464(7291):993–998, 2010.

[83] John G Tate, Sally Bamford, Harry C Jubb, Zbyslaw Sondka, et al. COSMIC: the Catalogue Of Somatic Mutations In Cancer. *Nucleic Acids Research*, 47(D1):D941–D947, 2019.

[84] Phasit Charoenkwan, Chonlatip Pipattanaboon, Chanin Nantasenamat, Md Mehedi Hasan, Mohammad Ali Moni, Watshara Shoombuatong, et al. Psrttca: A new approach for improving the prediction and characterization of tumor t cell antigens using propensity score representation learning. *Computers in Biology and Medicine*, 152:106368, 2023.

[85] Jesús Herrera-Bravo, Lisandra Herrera Belén, Jorge G Farias, and Jorge F Beltrán. Tap 1.0: A robust immunoinformatic tool for the prediction of tumor t-cell antigens based on aaindex properties. *Computational Biology and Chemistry*, 91:107452, 2021.

[86] Stanislav Sotirov and Ivan Dimitrov. Application of machine learning algorithms for prediction of tumor t-cell immunogens. *Applied Sciences*, 14(10):4034, 2024.

[87] Yang Lv, Ting Liu, Chang Liu, Yuchen Ma, Yunfei Liu, Ze Liu, and Yin Li. Lynet: Computational identification of tumor t cell antigens using convolutional and recurrent neural networks. *Computational Biology and Chemistry*, 120:108630, 2026.

[88] Jiahui Zhang, Rouyi Wang, Kuangqi Zhou, Tianshu Xiao, Lingyan Zhu, Yaosen Min, and Yang Wang. Pepbenchmark: A standardized benchmark for peptide machine learning. *International Conference on Learning Representations (ICLR)*, 2026. arXiv:2604.10531.

[89] Jorge Félix Beltrán Lissabet, Lisandra Herrera Belén, and Jorge G Farias. Ttagp 1.0: A computational tool for the specific prediction of tumor t cell antigens. *Computational biology and chemistry*, 83:107103, 2019.

[90] Zikun Yang, Paul Bogdan, and Shahin Nazarian. An in silico deep learning approach to multi-epitope vaccine design: a SARS-CoV-2 case study. *Scientific Reports*, 11:3238, 2021.

[91] Edison Ong, Mei U Wong, Anthony Huffman, and Yongqun He. COVID-19 coronavirus vaccine design using reverse vaccinology and machine learning. *Frontiers in Immunology*, 11:1581, 2020.

[92] Ottavia Spiga, Anna Visibelli, Francesco Pettini, Bianca Roncaglia, and Annalisa Santucci. SHASI-ML: a machine learning-based approach for immunogenicity prediction in salmonella vaccine development. *Frontiers in Cellular and Infection Microbiology*, 15:1536156, 2025.

[93] Benjamin Alexander Albert, Yunxiao Yang, Xiaoyu Li, Rishi Bhimani, Christie Ballantyne, and Rachel Karchin. Deep neural networks predict class I major histocompatibility complex epitope presentation and transfer learn neoepitope immunogenicity. *Nature Machine Intelligence*, 5:696–704, 2023.

[94] Anja Conev, Didier Devaurs, Mauricio Menegatti Rigo, Dinler Amaral Antunes, and Lydia E Kavraki. 3pHLA-score improves structure-based peptide-HLA binding affinity prediction. *Scientific Reports*, 12:10749, 2022.

[95] Jianjun Hu and Zhonghao Liu. DeepMHC: Deep convolutional neural networks for high-performance peptide-MHC binding affinity prediction. *bioRxiv*, 2017. Preprint.

[96] Youngmahn Han and Dongsup Kim. Deep convolutional neural networks for pan-specific peptide-MHC class I binding prediction. *BMC Bioinformatics*, 18:585, 2017.

[97] Limin Jiang, Hui Yu, Jiawei Li, Jijun Tang, Yan Guo, and Fei Guo. Predicting MHC class I binder: existing approaches and a novel recurrent neural network solution (BVLSTM-MHC). *Briefings in Bioinformatics*, 22(6):bbab216, 2021.

[98] Yi Lu, Le Mi, and Shixiong Zhang. DPCMHC: efficient prediction of MHC-peptide binding affinity by deep learning based on dual-padding convolution. *bioRxiv*, 2025. Preprint.

[99] Nasser Hashemi, Boran Hao, Mikhail Ignatov, Ioannis Ch. Paschalidis, Pirooz Vakili, Sandor Vajda, and Dima Kozakov. Improved prediction of MHC-peptide binding using protein language models. *Frontiers in Bioinformatics*, 3:1207380, 2023.

[100] William John Thrift, Nicolas W. Lounsbury, Quade Broadwell, Amy Heidersbach, Emily Freund, Yassan Abdolazimi, Qui T. Phung, Jieming Chen, Aude-Hélène Capietto, Ann-Jay Tong, Christopher M. Rose, Craig Blanchette, Jennie R. Lill, Benjamin Haley, and Lélia Delamarre. Towards designing improved cancer immunotherapy targets with a peptide-MHC-I presentation model, HLApollo. *Nature Communications*, 15:10752, 2024.

[101] Ruimeng Li, Ying Wang, Haozhou Li, Biyi Zhou, and Qinke Peng. Multimodal learning on heterogeneous subgraphs and LLMs representation for MHC-peptide binding affinity prediction. *BMC Bioinformatics*, 27:82, 2026.

[102] Hu Xu, Yuanli Ni, Zixuan Chai, Xuan Cui, Xia Lei, Limei Liu, Juanjuan Shan, and Cheng Qian. TransBindpMHCI: a transformer-based model for pan-specific MHC-I peptide binding prediction. *BMC Bioinformatics*, 27:117, 2026.

[103] A M Degoot, Faraimunashe Chirove, and Wilfred Ndifon. Trans-allelic model for prediction of peptide:MHC-II interactions. *Frontiers in Immunology*, 9:1410, 2018.

[104] Zhonghao Liu, Jing Jin, Yuxin Cui, Zheng Xiong, Alireza Nasiri, Yong Zhao, and Jianjun Hu. DeepSeqPanII: an interpretable recurrent neural network model with attention mechanism for peptide-HLA class II binding prediction. *IEEE/ACM Transactions on Computational Biology and Bioinformatics*, 19(4):2188–2196, 2022.

[105] Kazuhiro Ikkyu et al. MTL4MHC2: MHC class II binding prediction using multi-task learning from small training data. *Research Square*, 2022. Preprint.

[106] Ying Yu, Lipeng Zu, Jiaye Jiang, Yafang Wu, Yinglin Wang, Midie Xu, and Qing Liu. Structure-aware deep model for MHC-II peptide binding affinity prediction. *BMC Genomics*, 25(1):127, 2024.

[107] Timothy P Riley, Grant L J Keller, Angela R Smith, Lauren M Davancaze, Alyssa G Arbuiso, Jason R Devlin, and Brian M Baker. Structure based prediction of neoantigen immunogenicity. *Frontiers in Immunology*, 10:2047, 2019.

[108] Jeffrey Weber, Diego Chowell, Chirag Krishna, Timothy Chan, et al. Predicting HLA-I peptide immunogenicity with deep learning and molecular dynamics. *Research Square*, 2020. Preprint.

[109] Zicheng Hu, Patrick Wu, and Steven J Swanson. Evaluating the immunogenicity risk of protein therapeutics by augmenting T cell epitope prediction with clinical factors. *The AAPS Journal*, 27:33, 2025.

[110] Ko-Han Lee, Timothy J Sears, et al. NeoPrecis: enhancing immunotherapy response prediction through integration of qualified immunogenicity and clonality-aware neoantigen landscapes. *Nature Communications*, 17, 2026.

[111] Alessandro Montemurro, Viktoria Schuster, Helle Rus Povlsen, Amalie Kai Bentzen, Vanessa Jurtz, William D Chronister, Austin Crinklaw, Sine R Hadrup, Ole Winther, Bjoern Peters, Leon Eyrich Jessen, and Morten Nielsen. NetTCR-2.0 enables accurate prediction of TCR-peptide binding by using paired TCRα and β sequence data. *Communications Biology*, 4(1):1060, 2021.

[112] Alessandro Montemurro, Leon Eyrich Jessen, and Morten Nielsen. NetTCR-2.1: lessons and guidance on how to develop models for TCR specificity predictions. *Frontiers in Immunology*, 13:1055151, 2022.

[113] Ido Springer, Nili Tickotsky, and Yoram Louzoun. Contribution of T cell receptor alpha and beta CDR3, MHC typing, V and J genes to peptide binding prediction. *Frontiers in Immunology*, 12:664514, 2021.

[114] Tianshi Lu, Zhiwei Zhang, Jianzhu Zhu, Yan Wang, Peng Jiang, Xuequn Xiao, et al. Deep learning-based prediction of the T cell receptor–antigen binding specificity. *Nature Machine Intelligence*, 3:864–875, 2021.

[115] Jaspreet Kaur Dhanjal et al. TCR-ESM: employing protein language embeddings to predict TCR-peptide-MHC binding. *Computational and Structural Biotechnology Journal*, 23:165–173, 2024.

[116] Barthelemy Meynard-Piganeau, Christoph Feinauer, Martin Weigt, Aleksandra M Walczak, and Thierry Mora. TULIP: a transformer-based unsupervised language model for interacting peptides and T cell receptors that generalizes to unseen epitopes. *Proceedings of the National Academy of Sciences*, 121(24):e2316401121, 2024.

[117] Jiahao Ma, Hongzong Li, Jian-Dong Huang, Ye-Fan Hu, and Yifan Chen. Remodeling peptide-MHC-TCR triad binding as sequence fusion for immunogenicity prediction. *arXiv*, 2025. Preprint.

[118] Jiarui Li, Zixiang Yin, Zhengming Ding, Samuel J Landry, and Ramgopal R Mettu. TCR-EML: explainable model layers for TCR-pmhc prediction. *arXiv*, 2025. Preprint.

[119] Chenpeng Yu, Xing Fang, Shiye Tian, and Hui Liu. A unified cross-attention model for predicting antigen binding specificity to both HLA and TCR molecules (UnifyImmun). *Nature Machine Intelligence*, 7:278–292, 2025.

[120] Fan Li, Xinyang Qian, Xiaoyan Zhu, Xin Lai, Xuanping Zhang, and Jiayin Wang. TCRcost: a deep learning model utilizing TCR 3D structure for enhanced prediction of TCR-peptide-MHC binding. *Frontiers in Genetics*, 15:1346784, 2024.

[121] Ryan O Schenck, Eszter Lakatos, Chandler Gatenbee, Trevor A Graham, and Alexander R A Anderson. NeoPredPipe: high-throughput neoantigen prediction and recognition potential pipeline. *BMC Bioinformatics*, 20:264, 2019.

[122] Franziska Lang, Barbara Schrors, Martin Löwer, Özlem Tureci, and Ugur Sahin. NeoFox: annotating neoantigen candidates with neoantigen features. *Bioinformatics*, 37(22):4246–4247, 2021.

[123] Huiming Xia, My Hoang, Evelyn Schmidt, Susanna Kiwala, Joshua McMichael, Zachary L Skidmore, Bryan Fisk, Jonathan J Song, Jasreet Hundal, Thomas Mooney, Jason R Walker, S Peter Goedegebuure, Christopher A Miller, William E Gillanders, Obi L Griffith, and Malachi Griffith. pVACview: an interactive visualization tool for efficient neoantigen prioritization and selection. *Genome Medicine*, 16(1):132, 2024.

[124] Shihu Jiao, Quan Zou, Huannan Guo, and Lei Shi. ittca-rf: a random forest predictor for tumor t cell antigens. *Journal of translational medicine*, 19(1):449, 2021.

[125] Jen-Chieh Yu, Kuan Ni, and Ching-Tai Chen. Encap: Computational prediction of tumor t cell antigens with ensemble classifiers and diverse sequence features. *Plos one*, 19(7):e0307176, 2024.

[126] Nagina Bibi, Mukhtaj Khan, Salman Khan, Sumaiya Noor, Salman A Alqahtani, Abid Ali, and Nadeem Iqbal. Sequence-based intelligent model for identification of tumor t cell antigens using fusion features. *IEEE Access*, 12:155041–155054, 2024.

[127] Zhenjiao Du, Xingjian Ding, Yixiang Xu, and Yonghui Li. Unidl4biopep: a universal deep-learning architecture for binary classification in peptide bioactivity. *Briefings in Bioinformatics*, 24(3):bbad135, 2023.
